# A window into intracellular events in myositis through subcellular proteomics

**DOI:** 10.1007/s00011-025-01996-8

**Published:** 2025-01-31

**Authors:** Jennifer M. Peterson, Valérie Leclair, Olumide E. Oyebode, Dema M. Herzallah, Andrea L. Nestor-Kalinoski, Jose Morais, René P. Zahedi, Mazen Alamr, John A. Di Battista, Marie Hudson

**Affiliations:** 1 Department of Exercise and Rehabilitative Sciences, The University of Toledo, 2801 W. Bancroft St., MS 119, Toledo, OH 43606 USA; 2 Division of Rheumatology, Department of Medicine, McGill University, Montreal, QC Canada; 3 Department of Surgery, Advanced Microscopy and Imaging Center, University of Toledo, Toledo, OH USA; 4 Division of Geriatric Medicine and Research Institute, McGill University Health Centre, Montreal, QC Canada; 5 Segal Cancer Proteomics Centre, Lady Davis Institute for Medical Research, Montreal, QC Canada; 6 Manitoba Centre for Proteomics and Systems Biology, University of Manitoba, Winnipeg, MB Canada; 7 Department of Internal Medicine, University of Manitoba, Winnipeg, MB Canada; 8 Department of Biochemistry and Medical Genetics, University of Manitoba, Winnipeg, MB Canada; 9 Department of Neurology and Neurosurgery, McGill University, Montreal, QC Canada; 10 Department of Medicine and Experimental Medicine, McGill University Health Centre Research Institute, Montreal, QC Canada

**Keywords:** Myositis, Proteomics, Human, Muscle, Biopsy

## Abstract

**Objective and design:**

Idiopathic inflammatory myopathies (IIM) are a heterogeneous group of inflammatory muscle disorders of unknown etiology. It is postulated that mitochondrial dysfunction and protein aggregation in skeletal muscle contribute to myofiber degeneration. However, molecular pathways that lead to protein aggregation in skeletal muscle are not well defined.

**Subjects:**

Here we have isolated membrane-bound organelles (e.g., nuclei, mitochondria, sarcoplasmic/endoplasmic reticulum, Golgi apparatus, and plasma membrane) from muscle biopsies of normal (n = 3) and muscle disease patients (n = 11). Of the myopathy group, 10 patients displayed mitochondrial abnormalities (IIM (n = 9); mitochondrial myopathy (n = 1)), and one IIM patient did not show mitochondrial abnormalities (polymyositis).

**Methods:**

Global proteomic analysis was performed using an Orbitrap Fusion mass spectrometer. Upon unsupervised clustering, normal and mitochondrial myopathy muscle samples clustered separately from IIM samples.

**Results:**

We have confirmed previously known protein alterations in IIM and identified several new ones. For example, we found differential expression of (i) nuclear proteins that control cell division, transcription, RNA regulation, and stability, (ii) ER and Golgi proteins involved in protein folding, degradation, and protein trafficking in the cytosol, and (iii) mitochondrial proteins involved in energy production/metabolism and alterations in cytoskeletal and contractile machinery of the muscle.

**Conclusions:**

Our data demonstrates that molecular alterations are not limited to protein aggregations in the cytosol (inclusions) and occur in nuclear, mitochondrial, and membrane compartments of IIM skeletal muscle.

**Supplementary Information:**

The online version contains supplementary material available at 10.1007/s00011-025-01996-8.

## Introduction

The causes and pathology of idiopathic inflammatory myopathy (IIM), including dermatomyositis (DM) overlap myositis (OM), sporadic inclusion body myositis (sIBM), polymyositis (PM) and other subsets, are poorly understood. Characteristic features of these diseases include the presence of muscle weakness and poor endurance. Reduced cardiorespiratory fitness in this population suggests potential deficits in mitochondrial energy-generating pathways. Evidence that these deficits can be improved with exercise indicates that understanding the full range of molecular deficits in IIM will provide insight into treating these diseases [1].

Mitochondria are responsible for regulating the metabolic status of skeletal muscle. A ready supply of ATP energy is necessary to support muscular work. Mitochondria readily adapt their volume, structure, and function to chronic exercise, disuse, aging, and disease [2]. This is balanced by the onset of organelle turnover carried out by the mitophagy pathway to ensure a high functioning network of mitochondria for optimal ATP supply, maintaining muscle mass, and reducing apoptotic susceptibility over the longer term [3].

Previous investigations have demonstrated that nonspecific ultrastructural mitochondrial abnormalities (e.g., enlargement, loss of cristae of the inner mitochondrial membrane and inclusions), mutations/deletions, and changes in the proteome and transcription of genes associated with oxidative phosphorylation and mitochondrial function are present in IIM patients [4, 5]. It is postulated that mitochondrial dysfunction and protein aggregation in skeletal muscle lead to myofiber degeneration [6]. However, the pathways that cause these downstream effects are not understood and need to be examined to understand mechanisms of disease and reveal new potential therapeutic targets.

Proteomics on whole muscle lysates is challenging because the most muscle-abundant proteins dominate spectra (e.g., contractile proteins), failing to detect proteins such as intracellular signaling and trafficking proteins that are associated with muscle homeostasis and dysregulation. Previous proteomic analysis studies in IIM were performed on whole muscle lysates. They identified proteins involved in protein quality control and degradation (e.g., endoplasmic reticulum (ER) stress, heat shock proteins, the ubiquitin–proteasome system, autophagy, and vesicle transport) [7, 8]. Here we present insights into pathological pathways involved in IIM based on methods that enrich membrane-bound organelles in skeletal muscle, which includes mitochondria, nuclei, ER and sarcoplasmic reticulum (SR), Golgi apparatus, and plasma membrane) from muscle biopsies [9, 10].

## Materials and methods

### Study design and participants

Patients were selected from the Canadian Inflammatory Myopathy Study (CIMS) registry based on the presence of mitochondrial abnormalities on their muscle biopsies. Mitochondrial abnormalities were considered present if any of the following elements were described on clinical histopathological reports: (1) ragged-red fibers, (2) COX-reduced/negative fibers, and/or (3) subsarcolemmal oxidative activity. Additionally, a patient with HTLV-1-associated myopathy was included, as this entity is known to be associated with mitochondrial abnormalities [11]. This study was approved by the West-Central Montreal CIUSSS research ethics board (2021–2673). All participants provided written informed consent.

### Muscle biopsies

Muscle biopsies were processed at the Montreal Neurological Institute, a neuropathology referral center, and read by an experienced neuropathologist. All stains for primary study participants were performed as recommended by the European Neuromuscular Center (ENMC) for histological assessment of inflammatory muscle biopsies [12] with histochemical studies including hematoxylin–eosin (H&E), modified Gomori trichrome, and combined succinic dehydrogenase/cytochrome oxidase (COX-SDH) performed on 10 μm-thick fresh frozen sections.

Immunofluorescence was performed on fresh frozen sections and washes were performed with TBS-T (Tris-buffered saline, 0.1% Tween 20). RAB7A and MHC-1 staining: Sections were fixed in ice-cold acetone for 1 min, permeabilized in 1% Triton-X for 5 min, blocked in 5% goat serum/TBS-T for 1 h, incubated in primary antibody for 1 h (MHC-1/HLA-ABC: 1:50, Invitrogen, MA511723 clone W6/32; RAB7A: 1:20, (Sigma-Aldrich Cat# HPA006964, RRID:AB_1856015), incubated in secondary antibody (1:2000, Alexa Fluor Plus 488, Invitrogen) for 30 min (MHC-1: goat-anti-mouse; RAB7A: goat-anti-rabbit), and coverslip mounting medium with DAPI was used (DAPI Fluoromount-G, Southern Biotech). For CSN7A/COPS7A and HSP70-1 staining: Sections were fixed in 4% methanol-free formaldehyde for 10 min, then antigen retrieval was performed. Slides were incubated in 10 mM Na-citrate buffer (pH 6.5) warmed to 65 C in a water bath. Upon slide immersion, the water bath temperature was raised to 92 °C (~ 30 min incubation to reach temperature). Once the temperature was reached, slides were incubated at 92 °C for 11 min then the water bath was shut off and slides cooled slowly for 30 min. Procedures were then completed as with MHC-1 and RAB7A as stated above starting with permeabilization step. Primary antibodies: CSN7A/COPS7A: 1:20, (Sigma-Aldrich Cat# HPA026915, RRID:AB_1847156; HSPA1A/HSP70-1: 1:50, (Santa Cruz Biotechnology Cat# sc-32239, RRID:AB_627759). Antibodies for RAB7A, CSN7ACOPS7A, and HSPA1A/HSP70-1 have been validated on normal skeletal muscle tissue and approved for immunostaining through the Human Protein Atlas proteinatlas.org [13].

### Confocal microscopy

Images were acquired on a Leica TCS SP5 laser scanning confocal microscope (Leica Microsystems, Bannockburn, IL) equipped with conventional solid state and a Ti-sapphire tunable multiphoton laser (Coherent, Santa Clara, CA). Images were acquired in the 512 × 512 format in the XYZ plane in 1 µm steps with a 40X oil objective (NA 1.25) in sequential scan mode.

### Protein extraction and preparation of membrane-bound organelles

Total soluble extract from frozen muscle biopsy tissue was used for the preparations [14]. Preparations were prepared as previously described for mitochondria extraction with noted modifications [9, 10]. Note this preparation results in the isolation of membrane-bound organelles rather than only mitochondria. Briefly, muscle tissue was minced and incubated with 10% nagarse in ionic medium (100 mm sucrose, 10 mm EDTA, 100 mm Tris–HCl, 46 mm KCl, pH 7.4) for 5 min, washed with ionic medium containing 0.5% BSA, homogenized, and centrifuged (500×*g*, 10 min, 4 °C). The supernatant was centrifuged (12,000×*g*, 10 min, 4 °C), and the partially purified pellet was washed twice with ionic medium containing 0.5% BSA and the final pellet was stored at − 80 °C in suspension medium (230 mm mannitol, 70 mm sucrose, 0.02 mm EDTA, 20 mm Tris–HCl, 5 mmK_2_HPO_4_, pH 7.4).

### LC MS/MS and database analysis

LC–MS/MS analysis of tryptic peptides was carried out on a Thermo Scientific Orbitrap Fusion Tribrid mass spectrometer with Dionex UltiMate 3000 RSLCnano system as previously described with modifications [10]. Briefly, MS/MS spectra were collected with an Orbitrap hybrid mass spectrometer, using a top-ten method, dynamic exclusion repeat count of 1 and a repeat duration of 30 s. MS spectra were collected in the Orbitrap component of the mass spectrometer and MS/MS spectra were collected in the LTQ. Mascot and X!Tandem search engines with Scaffold Q+ were employed for data integration and visualization. MS/MS spectra were additionally evaluated using TurboSequest in a Proteomics Browser Package. SwissProt (April 2017) and human Uniprot databases (October 2017) were used. Only proteins at a 1% false discovery rate and having at least two unique peptides were included in the analysis. Spectral counts from Scaffold were used to calculate normalized spectral abundance factor (NSAF) values for each sample [15]. The three control samples were run as technical duplicates and the median relative standard deviation over the technical replicates was assessed (28%) and for each control sample, the average NSAF values were determined for all proteins based on the 2 technical replicates. For the initial analysis, all myopathy patients were analyzed as one group (IIM plus mitochondrial myopathy) and compared to controls. Proteins that were not quantified in at least ½ of the (IIM plus mitochondrial myopathy) or 2/3 of the control group samples were not further considered. Then, missing values were imputed using the minimum NSAF determined for the same sample. For the three controls, the average NSAF values (avg CT1, avg CT2, avg CT3) were determined based on the technical replicates and were then used together with the NSAF values of the (IIM plus mitochondrial myopathy) group to determine *p*-values (2-paired T-test), as well as disease/control ratios (median NSAF (IIM plus mitochondrial myopathy)/average NSAF (avg CT1-3)). Proteins with *p*-values < 0.05 (*) and < 0.01 (**) and fold change ≤ or ≥ 3 were considered differentially regulated.

### Informatics and visualization

Perseus [16] was used to create heatmaps based on unsupervised clustering as well as for principal component analysis (PCA) based on NSAF values. ToppFun from the ToppGene Suite [17] was used to identify functional enrichment within the two main protein clusters generated from the heatmap. Features investigated were Gene Ontology (GO): Molecular Function, GO: Biological Process, and GO: Cellular Component. Default statistics were used including Probability density function FDR *p* ≤ 0.05. Proteomaps [18] version 2.0 was the graphical tool used to visualize composition and abundance of detected proteins. NSAF values were provided as input to assess relative expression levels of proteome subsets based on functional categories. Tree and label level 2 (functional categories) were the settings selected to generate maps. For Reactome and Metascape databases, IIM patients were compared to control samples (mitochondrial myopathy sample was omitted). A 2.83-fold change cut-off was used and overexpressed and underexpressed proteins were analyzed separately in each database. The Reactome database [19] (version 80) was used to identify pathways where greater or fewer proteins than would be expected by chance were differentially expressed. FDR *p* ≤ 0.05 cut-off was used for data presentation and exact *p* value is presented in data tables. Visualization of physically interacting proteins (protein complexes) that were differentially expressed was performed using Metascape [20].

## Results

### Clinical and histological characteristics of patients

Fourteen subjects (9 females; 5 males) were included in our analysis; 11 patients with muscle disease and 3 controls. Group and subject characteristics are detailed in Table 1. The average age at the time of biopsy was 59.5 years for muscle disease patients and 67 years for controls. All subjects except the 4 sIBM patients were undiagnosed at the time of biopsy. One patient was subsequently diagnosed with a suspected inherited mitochondrial myopathy (MM), and the 6 remaining patients were diagnosed with IIMs (DM (n = 2), OM (n = 3) and PM (n = 1)). Of the IIM patients, 4 (40%) were taking immunosuppressants at time of biopsy. Two control biopsies were from healthy individuals participating in a study on aging, and one control biopsy was from a patient with fibromyalgia.

**Table 1:** Study groups and subject characteristics

Group/Subject	Age	Sex	Current diagnosis	Autoantibodies	Notes	Treatment at biopsy
CT-1	86	F	Healthy control	NA		None
CT-2	73	F	Healthy control	NA		None
CT-3	42	F	Fibromyalgia	None		None
MM-4	52	F	Suspected mitochondrial myopathy	None		Prednisone 10 mg/day
sIBM-5	46	M	sIBM	Anti-NT5c1A	Large granular lymphocytic leukemia	MTX
sIBM-6	60	F	sIBM	Anti-NT5c1A		None
sIBM-7	67	M	sIBM	None		None
sIBM-8	40	M	sIBM	Anti-NT5c1A	Large granular lymphocytic leukemia	None
DM-9	70	F	DM	Anti-TIF1g		None
DM-10	52	M	DM	Anti-Mi2, Anti-Ro52		Prednisone 100 mg/day for 7 days
PM-11	39	F	PM	None	HTLV1 infection	None
OM-12	76	F	OM	Anti-NT5c1A, Anti-Ro52, RF	Raynaud, head drop, ILD	Prednisone 50 mg/day for 3 days
OM-13	49	M	OM	Anti-CCP, RF, Anti-NT5c1A, Anti-Ro52	Polyarthritis, refractory to immunosuppression	HCQ
OM-14	81	F	OM	Anti-NT5c1A, Anti-Ro52	Raynaud, ILD	None

*CT * control; *MM* mitochondrial myopathy; *sIBM* sporatic inclusion body myositis; *DM* dermatomyositis; *PM* polymyositis; *OM*overlap myositis; *HTLV1* human T lymphotropic virus type 1; *NA* not assessed; *RF* rheumatoid factor; *MTX* methotrexate; *HCQ* hydroxychloroquine

**Fig. 1:**
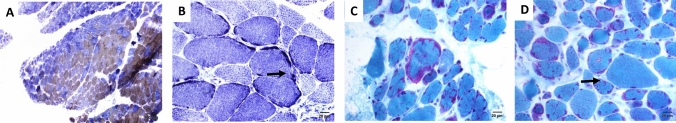
Histopathological hallmarks indicative of mitochondrial abnormalities. **A** Abundant COX-negative (blue) fibers on combined COX-SDH enzyme histochemistry in subject OM-13, **B** Subsarcolemmal oxidative activity on NADH enzyme histochemistry—ragged-blue fibers (arrow) in subject OM-13, **C** Ragged-red fibers seen with Gomori trichrome staining in subject OM-12, **D** Rimmed vacuole (arrow) seen with Gomori trichome staining in subject OM-12. Cytochrome c oxidase (COX), succinate dehydrogenase (SDH), overlap myositis (OM), nicotinadmide adenine dinucleotide + hydrogen (NADH)

Histological staining of muscle biopsies from all patient samples except PM-11 showed evidence of mitochondrial abnormalities by reduction/absence of cytochrome c oxidase (COX)-stained muscle fibers (Table 2). A representative image in Fig. 1A demonstrates a biopsy with reduced/absent COX stained myofibers (brown color) concurrent with an abnormal increase in succinate dehydrogenase (SDH) staining intensity (purple). Both COX and SDH are enzymes expressed in mitochondria as part of the electron transport chain and therefore every muscle fiber should express some of each enzyme, albeit at varying degrees (type 1 fibers stain darker than type II fibers). Mitochondrial abundance, distribution, and proper electron transport chain assembly can be estimated with COX-SDH staining. Reduction and absence of COX stained myofibers can indicate degradation, improper assembly, or mutations in complex IV. SDH is specific to complex II of the electron transport chain and is more generally used to visualize abundance and distribution of mitochondria. Ragged-blue fibers, identified as fibers showing concentrated abnormal sarcolemmal SDH staining, were found in the biopsies of sIBM-7 and OM-13, providing further evidence of mitochondrial abnormalities (Fig. 1B; oxidative abnormalities, scored in Table 2). Abnormal mitochondrial abundance and sarcolemmal aggregation, visualized using Gomori trichrome stain and identified as ragged-red fibers, were found in the biopsies of sIBM-7 and OM-13 (Fig. 1C; Table 2). Three out of four sIBM patients (sIBM-5-7) displayed rimmed vacuoles as did one overlap myositis patient (OM-12) and one dermatomyositis patient (DM-10) (Fig. 1D; Table 2). Additionally, three out of four sIBM patients (sIBM 5, 6, 8) and all 3 OM patients tested seropositive for anti-NT5c1A antibodies (Table 1). Anti-NT5c1A antibodies have been detected in the serum of IIM patients as well as other autoimmune diseases [21]. Within the IIM subtypes these antibodies have been reported at a higher frequency in sIBM patients compared to other IIM subtypes [22–24]. Previous investigations have reported patients seropositive for anti-NT5c1A antibodies are more likely to have a higher number of COX-negative fibers in their biopsy findings, indicating a potential relationship between mitochondrial abnormalities and anti-NT5c1A antibodies [22, 24]. Control biopsies did not show any notable abnormalities (Table 2).

**Table 2:** Scoring of histopathological muscle biopsy features

Group/Subject	Perifascicular atrophy	Necrosis	Inflammation	COX-reduced/negative fibers	Ragged-red fibers	Subsarcolemmal oxidative abnormalities (Ragged-blue fibers)	Rimmed vacuoles
CT-1	0	0	0	0	0	0	0
CT-2	0	0	0	0	0	0	0
CT-3	0	0	0	0	0	0	0
MM-4	0	+	0	++	0	0	0
sIBM-5	0	+	++	++	0	0	++
sIBM-6	0	0	+	+	0	0	+
sIBM-7	0	++	++	++	+	+	++
sIBM-8	0	+	+	++	0	0	0
DM-9	+	++	++	++	0	0	0
DM-10	0	++	+	++	0	0	+
PM-11	0	+	+	0	0	0	0
OM-12	0	++	++	++	++	0	+
OM-13	0	++	++	++	0	++	0
OM-14	0	++	++	++	0	+	0

Present (+), Marked (++), Absent (0)

### Proteomics overview

In total, 736 proteins were quantified (Supplemental Table 1). Of the quantified proteins, 42% (308) could be categorized as mitochondrial. Proteins localizing to other membranous organelles, including sarcoplasmic/endoplasmic reticulum, Golgi apparatus, nuclei, and cell membranes were additionally identified, along with some cytoskeletal proteins (Supplemental Table 1). Test–retest reliability assessments were done using data obtained from technical duplicates of the three control muscle samples. Pearson correlations between each pair were CT 1 = 0.92, CT2 = 0.93, and CT 3 = 0.92. For subsequent analyses data from each pair of control samples were averaged.

### Membrane-bound proteins are distinctly different between IIM and control muscle

**Fig. 2:**
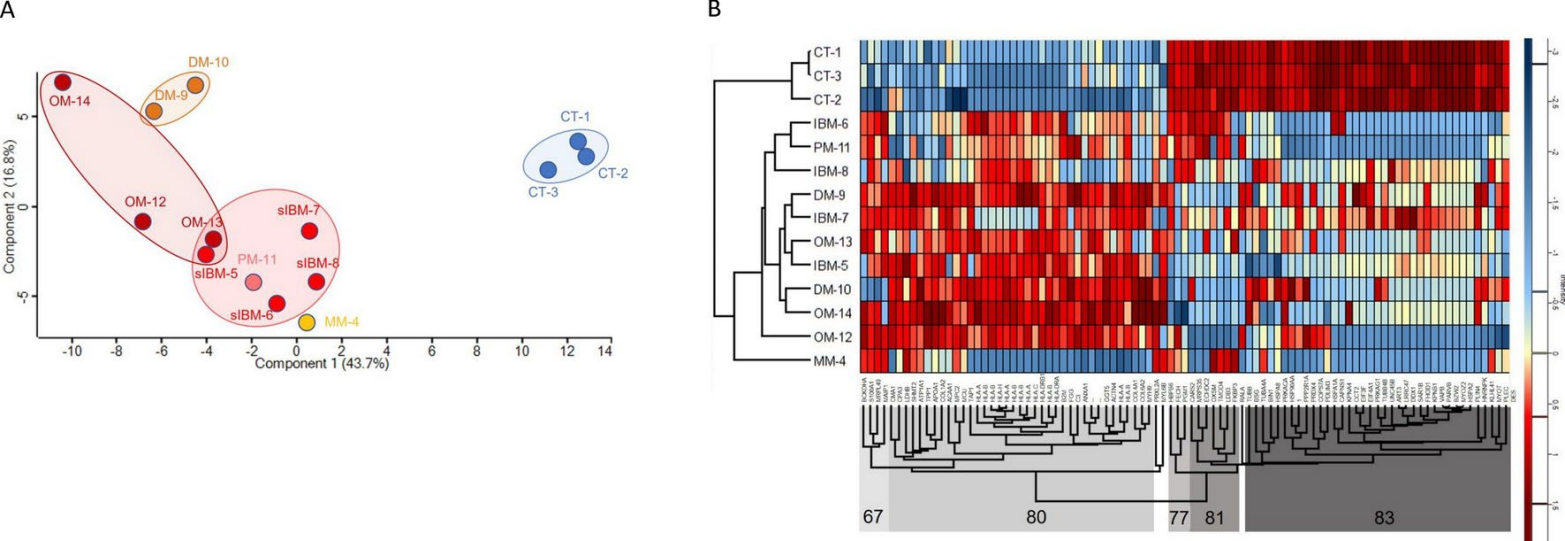
Characteristics of muscle biopsy-derived proteomes. **A** Principal Component Analysis (PCA) showing clustering of control and patient samples. **B** Heatmap representing unsupervised clustering of the relative normalized expression of 91 proteins that were significantly regulated between groups. Both analyses clearly separate controls from muscle disease samples, while concurrently demonstrating similarities between patients with different diagnoses. Control (CT), sporadic inclusion body myositis (sIBM), dermatomyositis (DM), overlap myositis (OM), mitochondrial myopathy (MM)

Principal component analysis (PCA) was used as an initial statistical method to visualize differences between muscle biopsy-derived proteosome samples (Fig. 2A). Normal control samples clustered separately from all muscle disease samples. Within the muscle disease samples, the patient with mitochondrial myopathy clustered separately from the IIM patients, and the DM patients clustered separately from other IIM subtypes. sIBM patients clustered together and included the PM patient (HTLV-1 positive) and one of the OM patients (OM-13). A separate cluster with OM patients could be visualized and included one of the sIBM patients (sIBM-5).

We next wanted to determine if IIM patient muscle could be distinguished from control muscle based on differential protein expression using our approach to combine membrane-bound organelle isolation with mass spectrometry. Ratios and *p*-values were used to determine significantly regulated proteins (*p* < 0.05, fold change > threefold) comparing muscle disease (sIBM, DM, PM, OM, mitochondrial myopathy) to control samples. For the 91 unique proteins that were significantly regulated, we used unsupervised hierarchal clustering and revealed a clear separation between control and muscle disease samples (Fig. 2B). Further, the mitochondrial myopathy patient grouped independently from all the IIM samples. Proteins in each of the highlighted clusters are listed in Supplemental Table 2. These initial results suggest the intriguing possibility there is a potential for proteomic analysis from isolated membrane-bound organelles of human muscle biopsies to identify broad IIM patient groups. Future studies with more patient samples will be needed to explore this possibility.

The largest differentially expressed Gene Ontology clusters contained proteins that function in antigen processing and presentation (cluster 80; overexpressed in myositis samples), protein folding/ER stress and cytoskeletal and myofiber binding (cluster 83; predominantly underexpressed in myositis samples) (Table 3).

**Table 3:**
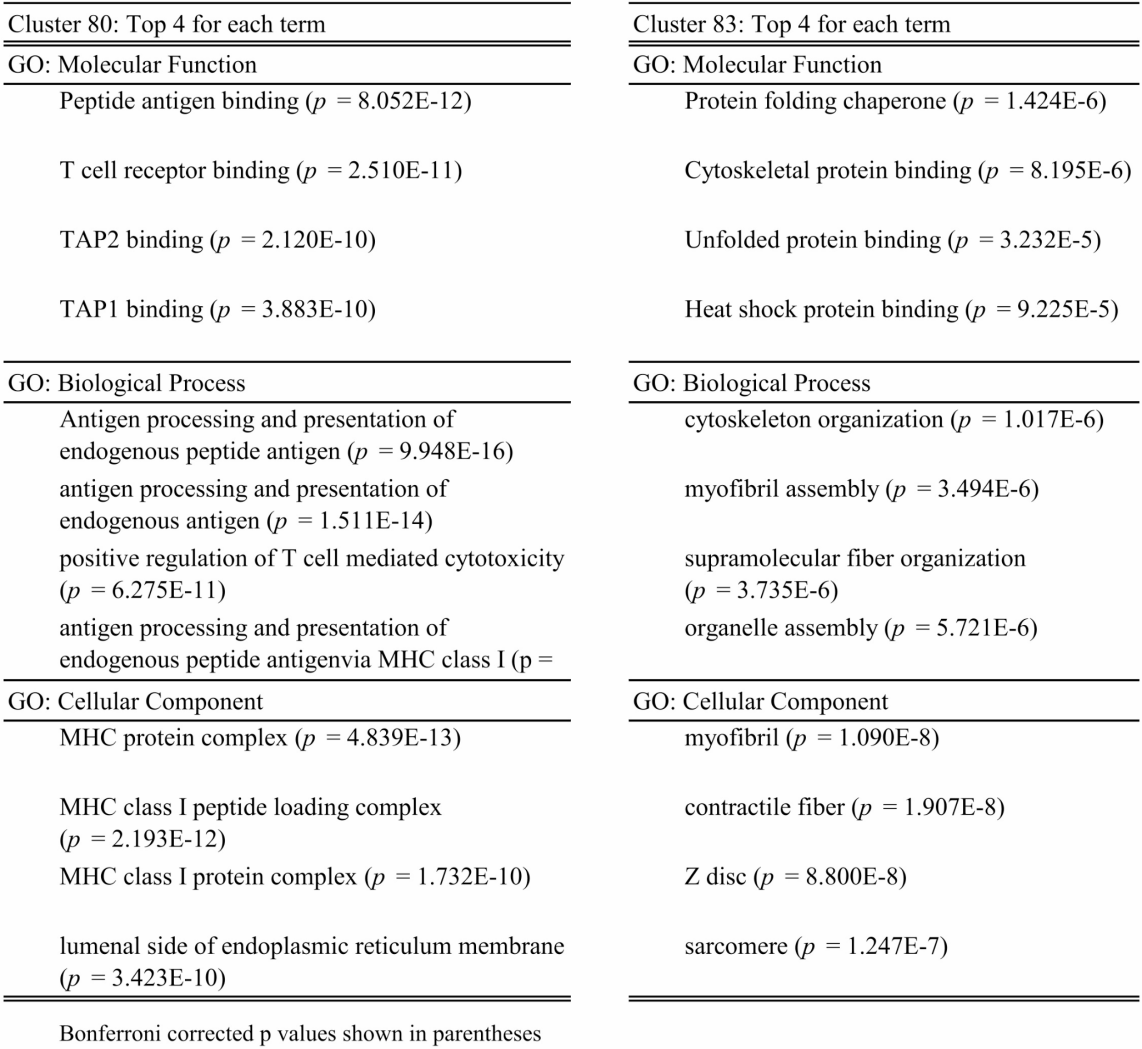
Top gene ontology (GO) terms for the two largest protein clusters in the heatmap

Bonferroni corrected *p* values shown in parentheses

**Fig. 3:**
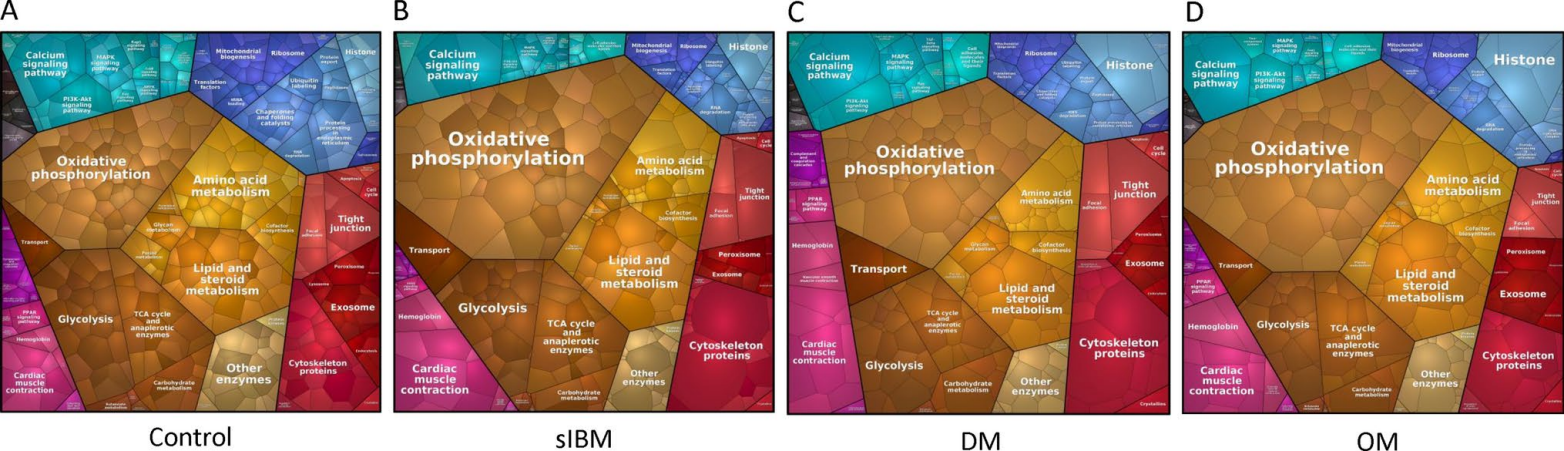
Proteomaps visualizing functional classes of identified proteins. **A** control, **B** sporadic inclusion body myositis (sIBM), **C** dermatomyositis (DM), and **D** overlap myositis (OM) samples

Next, we visualized the relative quantitative composition of analyzed proteomes between groups. These proteomap depictions provide visual images to highlight the proportions of analyzed proteomes that are dedicated to performing specific cellular functions, thus representing how the muscle’s energy focus differs in each IIM subtype from control (Fig. 3A–D). The predominant proportion of all the muscle biopsy-derived proteomes, including control, was involved in metabolism (yellow/brown color), consistent with our methods of isolating membrane-bound organelles. Collectively, even where general proportions did not differ greatly from control, the composition of each functional group (e.g. abundance of particular proteins in a group) was markedly different within and between the IIM subgroups compared to controls. Interestingly, metabolism was overrepresented in sIBM and OM, but not DM compared to controls. Within the category of metabolism, oxidative phosphorylation and transport were overrepresented in all IIM subtypes compared to controls, changing the balance within metabolic pathways. For example, the tricarboxylic acid (TCA) cycle was underrepresented compared to control. Together, these results suggest dysfunction in the balance and process of energy production with myositis muscles.

## Mitochondrial inner membrane protein expression

**Fig. 4:**
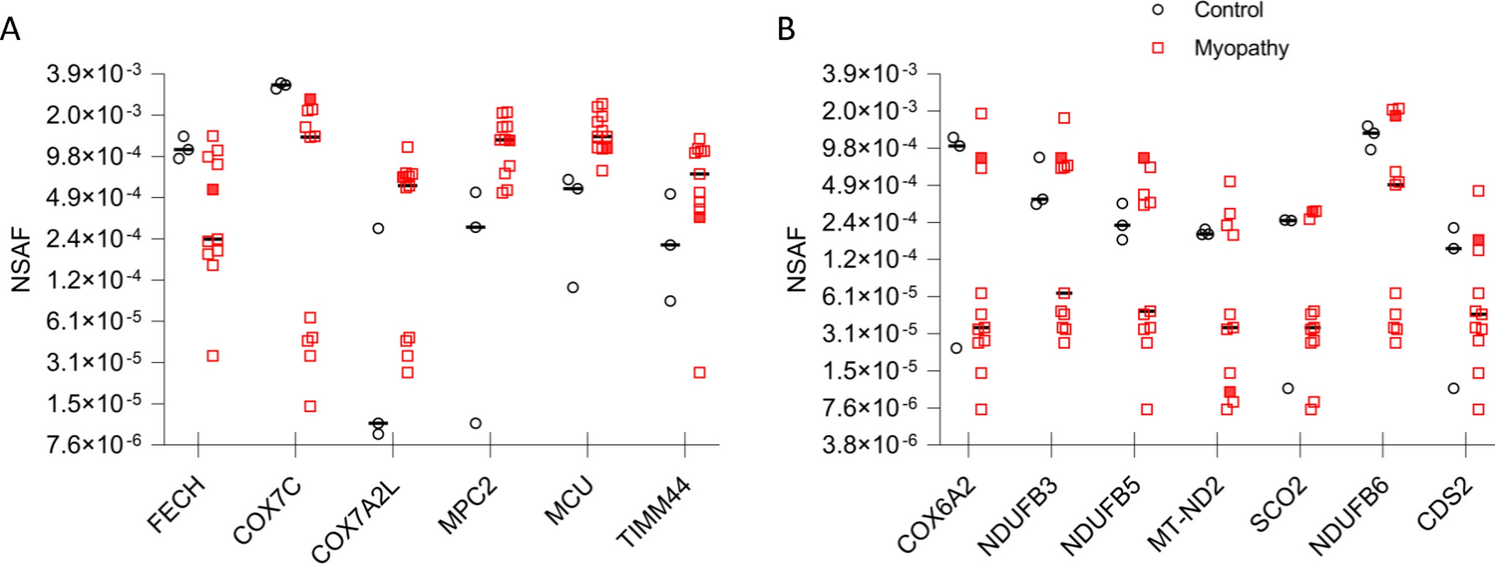
Mitochondrial inner membrane protein expression. Expression levels of proteins localized to the inner mitochondrial membrane. **A** Proteins with threefold or greater change and significantly different from control, **B** Proteins with threefold or greater change from control but not statistically different. Mitochondrial myopathy patient (MM-4) is indicated by a filled red square. Normalized spectral abundance factor (NSAF), Ferrochelatase, Mitochondrial (FECH), Cytochrome C Oxidase Subunit 7C, Mitochondrial (COX7C), Cytochrome C Oxidase Subunit 7A-Related Protein, Mitochondrial (COX7A2L), Mitochondrial Pyruvate Carrier 2 (MPC2), Mitochondrial Calcium Uniporter (MCU), Translocase Of Inner Mitochondrial Membrane 44 (TIMM44), Cytochrome C Oxidase Subunit 6A2, Mitochondrial (COX6A2), NADH:Ubiquinone Oxidoreductase Subunit B3 (NDUFB3), NADH:Ubiquinone Oxidoreductase Subunit B5 (NDUFB5), Mitochondrially Encoded NADH:Ubiquinone Oxidoreductase Core Subunit 2 (MT-ND2), Synthesis Of Cytochrome C Oxidase 2 (SCO2), NADH:Ubiquinone Oxidoreductase Subunit B6 (NDUFB6), CDP-Diacylglycerol Synthase 2 (CDS2)

Patients were chosen for this study based on histological abnormalities, particularly loss of COX staining. We next examined the data for proteins that localize to the mitochondrial inner membrane that could account for the loss of COX expression. Six proteins were differentially expressed using the criteria of (*p* < 0.05, fold change > threefold) (Fig. 4A). Variability in expression of these proteins was observed between patients and in some cases between controls. Previous studies have shown various deletions and mutations in mitochondrial DNA demonstrating that mitochondrial abnormalities may be due to differing causes among these patient groups [25–27]. To further investigate mitochondrial abnormalities that may only affect a subset of our tested patients, we broadened the search criteria to include proteins that met the fold change criteria but not the *p*-value (fold change > threefold). This led to the discovery of 7 additional proteins (Fig. 4B) that exhibited expression changes that could contribute to the observed mitochondrial pathology seen in these patients.

## Antigen processing and presentation components are upregulated in IIM muscle

**Fig. 5:**
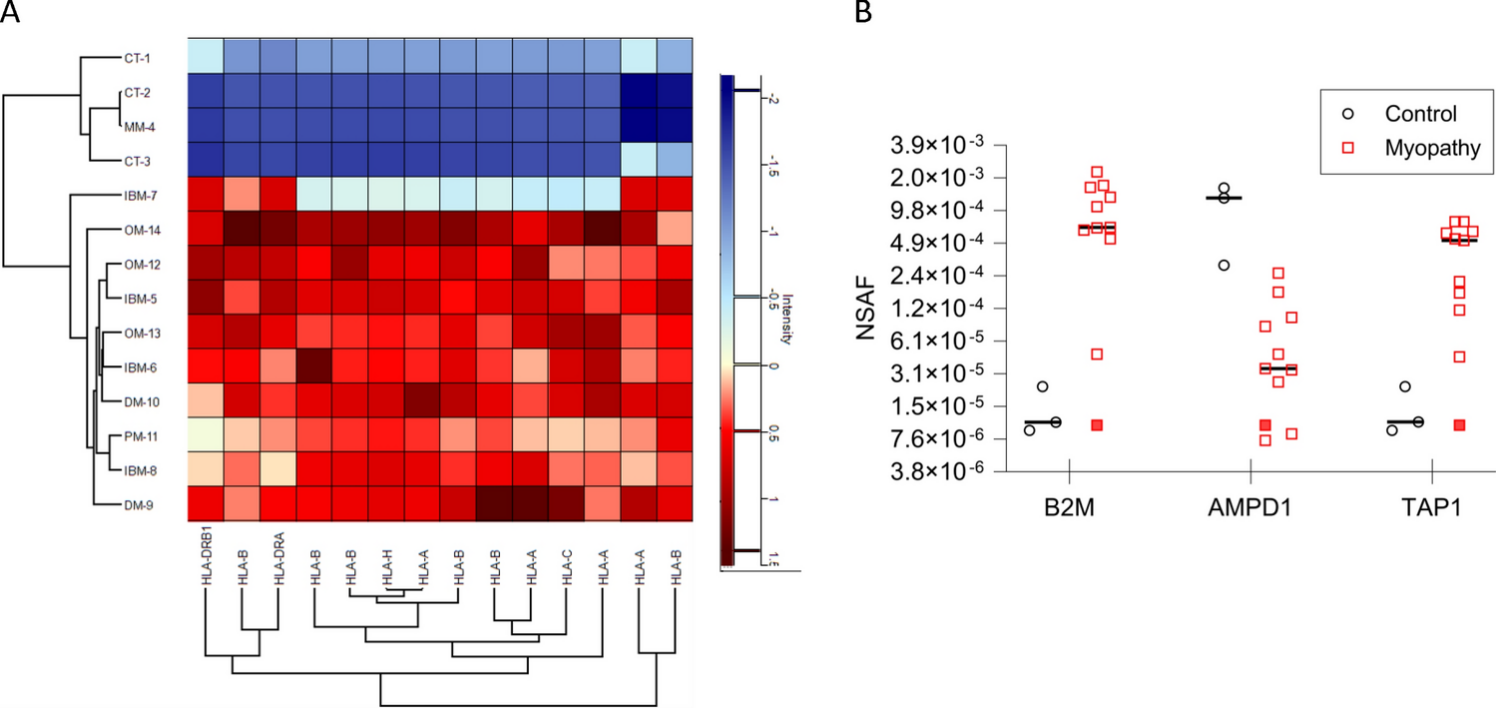
Profile of immune-associated proteins overexpressed in IIM patients. **A** Heatmap illustrating HLA protein expression in subjects. **B** Expression levels of proteins associated with antigen processing and presentation. Mitochondrial myopathy patient (MM-4) is indicated by a filled red square. Normalized spectral abundance factor (NSAF), Beta-2-microglobulin (B2M), adenosine monophosphate deaminase 1 (AMPD1), transporter 1, ATP binding cassette subfamily B member (TAP1)

A prominent feature of myositis muscle is the aberrant upregulation of antigen processing and presentation components. MHC class I expression in IIM muscle has been extensively documented and shown to perpetuate disease features, whereas MHC class II molecule expression has been described but is less well characterized [28, 29]. Consistent with this known disease phenotype, proteins involved with antigen processing and presentation were overexpressed in IIM compared to control muscle, but not in muscle from the mitochondrial myopathy patient (Fig. 5A, B). Classical MHC class I molecules consist of an alpha protein dimerized with a Beta-2-Microglobulin (B2M) protein. Each individual is capable of expressing 3 alpha MHC class I proteins (HLA-A; HLA-B, HLA-C). These class I molecule components were the most highly overexpressed in our IIM patient samples (Fig. 5A, B). MHC class II molecules are comprised of a dimer with an alpha and a beta protein. Patient samples showed modest overexpression of HLA-DRA and HLA-DRB1 (Fig. 5B). IIM patients in this study also overexpressed the non-classical HLA-H protein. TAP1 (Transporter 1, ATP Binding Cassette Subfamily B Member), part of the complex responsible for transporting antigens to MHC class I molecules, was additionally overexpressed (Fig. 5B). Gene expression was previously shown to be upregulated for TAP1 and further solidifies the strong signature of antigen processing and presentation proteins in IIM patients [30].

Acquired deficiency of adenosine monophosphate deaminase 1 (AMPD1) expression has been validated in IIM patient muscles and shown to contribute to muscle weakness in an inflammatory myositis mouse model [31–34]. Although AMPD1 did not meet the criteria for inclusion in our initial analysis (Fig. 2B), we identified and found this protein to be underexpressed in most myositis compared to control patients (Fig. 5B) as expected (*p* = 0.125; fold change = − 26.17). Underexpression of AMPD1 was noted in the patient with a mitochondrial myopathy (MM-4) as well. Just as we observed in searching for mitochondrial inner membrane protein differences, using a *p*-value cutoff is helpful in identifying the most consistent differentially expressed proteins, but presents challenges when trying to search for novel pathways in a highly heterogeneous patient population.

### Pathway analysis comparing myositis muscle to control muscles

To investigate if proteomics performed on the membrane-bound organelle fraction of muscle biopsies could identify novel proteins and/or pathways potentially involved in IIM, and/or provide more direct information regarding known dysregulated pathways, we interrogated databases aimed at biomolecular pathway discovery (Reactome) and physical interactions between proteins (Metascape). After excluding the patient with a mitochondrial myopathy (MM-4), all proteins differentially expressing 2.83-fold or greater change from control (1.5 log fold change) were used for the analysis. *p*-value cutoff was not used to account for the heterogenicity in patients. We identified 168 underexpressed proteins and 60 overexpressed proteins (Supplemental Table 3). The terms myositis and myopathy were queried in PubMed with each differentially regulated protein’s UniProt name. Based on these criteria, 28 of the 228 dysregulated proteins have been previously reported as dysregulated in IIM (Supplemental Table 3).

### Underrepresented pathways and interacting protein network

Using the Reactome database, 125/168 underexpressed proteins were curated and mapped to cellular pathways which revealed 92 significantly underrepresented pathways (FDR adjusted *p* ≤ 0.05). These pathways were grouped based on cellular functions. Broad categories along with their associated proteins are presented in Table 4, and an expanded comprehensive table containing pathway names and *p*-values in Supplemental Table 4. Twenty-six (out of 30) of the top pathways represented 6 broad cellular functions: cellular response to stress, autophagy, synaptic communication, programmed cell death, muscle contraction, and membrane trafficking. Significant, albeit less prominent pathways discovered in the analysis include oncogenic MAPK, calcium, and Rho GTPase signaling pathways, cell cycle, protein folding, metabolism of proteins and carbohydrates, mitochondrial energy production, intracellular vesicle transport, and chemical synapse transmission.

**Table 4:** Significantly underrepresented cellular pathways in IIM

Pathway category	Submitted entities found
Cellular response to stress	CAMK2B; HSPA8; CAMK2D; HSP90AA1; CAMK2A; HSPA2; HSPA1A; TUBB; TUBB4B; TUBA4A; CAP2; RPLP1; COX7C; VAPA; SCO2; LMNA; RPLP2; PREB; HIST1H1C
Autophagy	TOMM70; HSPA8; HSP90AA1; TUBB; PLIN4; UBE2N; PRKAG1; PLIN3; VIM; TUBB4B; TUBA4A
Programmed cell death	LMNA; VIM; HMGB1; DNM1L; KPNB1; PLEC; HIST1H1C; HSP90AA1; PDCD6IP; LMNA;
Muscle contraction	MYBPC2; DES; ACTN3; TMOD4; TNNC2; TNNI2; VIM; CAMK2B; CAMK2D; CAMK2A; CACNB1; STIM1; VCL
Synaptic communication	CAMK2B; CAMK2D; TUBB; CAMK2A; TUBB4B; TUBA4A; PRKAR2A; PRKAG1
Membrane trafficking	RALA; STXBP3; TUBB; PRKAG1; TUBB4B; TUBA4A; RAB18; CAP2; HSPA8; SAR1B; USO1; COPS7A; COPS4; RAB21; COPS3; BIN1; PLIN4; PLIN3; PREB; RAB7A; HSP90AA1; PPP2R1A;
Citric acid cycle and respiratory electron transport	PDP1; NDUFB6; NDUFB5; IDH3G; NUBPL; SCO2; ADHFE1; NDUFB3; BSG; D2HGDH; MT-ND2; COX7C
Cell cycle, mitotic	PPP2R1A; TUBB; LMNA; TUBB4B; TUBA4A; KPNB1; HSP90AA1
Oncogenic MAPK signaling	CAMK2B; MAP2K1; CAMK2D; PPP2R1A; CAMK2A; LMNA; VCL
Diseases of programmed cell death	PRDX4; CAPNS1; LMNA
Protein folding	CCT2; TUBB; TUBB4B; TUBA4A
Metabolism of proteins	EIF4A1; TSFM; MRPS9; MRPS35; QARS; RPLP1; RPLP2; EIF3F; MRPL37; EEF2; CARS2; HSPA8; EEF2; ART3; SAR1B; PRKCSH; USO1; COPS7A; RAB21; PCMT1; MLEC; LMCD1; CAP2; RAB2A; TOMM70; HSPA8; CCT2; TUBB; GPT2; TUBB4B; TUBA4A; KLHL41; COPS4; COPS3; MAVS; HNRNPK; HRC; RAB18; UBE2N; PREB; RAB7A; GPT2
Calcium-dependent events	CAMK2B; CAMK2D; PRKAR2A; CAMK2A
Signaling by Rho GTPases, Miro GTPases and RHOBTB3	HINT2; VAPB; STBD1; CAP2; RAB7A; COPS4; CCT2; HSP90AA1; VIM; TUBB; TUBB4B; TUBA4A
Vesicle-mediated transport	SAR1B; TUBB; USO1; PREB; TUBB4B; TUBA4A; CAP2; HSPA8; RALA; HSP90AA1; STXBP3; PRKAG1; COPS7A; COPS4; RAB21; COPS3; BIN1; RAB18; PLIN4; PLIN3; PREB; RAB7A
Transmission across chemical synapses	CAMK2B; CACNB1; HSPA8; CAMK2D; TUBB; PRKAR2A; CAMK2A; PRKAG1; TUBB4B; COMT; TUBA4A
Metabolism of carbohydrates	GYS1; UGP2; AGL; PGM1

**Fig. 6:**
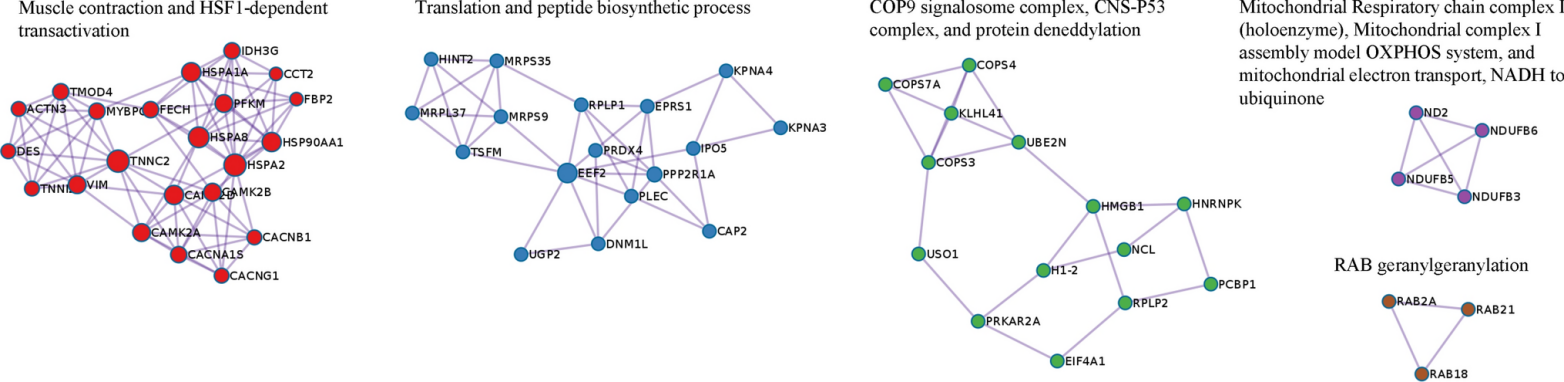
Underrepresented physically Interacting Protein Networks. Dysregulated proteins that physically interact with one another are visualized in networks. Lines show which proteins within the network are physically complexed with one another

When physically interacting proteins were examined, underexpressed proteins in IIM muscle formed interacting networks involved in muscle contraction and heat shock responses, protein translation, regulation of cellular protein ubiquitination, mitochondrial energy production, and vesicle trafficking (Fig. 6). These networks were also revealed in our broader analysis analyzing underrepresented proteins (Table 4 and Supplemental Table 4), albeit this analysis shows more specific parts of the pathways where multiple physically interacting proteins are underexpressed in IIM muscle, which could assist with identifying therapeutic targets.

### Overrepresented pathways and interacting protein network

Using the Reactome database, 56/60 overexpressed proteins were curated and mapped to cellular pathways and revealed 60 significantly overrepresented pathways (FDR adjusted *p* ≤ 0.05). These pathways were grouped based on cellular functions. Broad categories along with their associated proteins are presented in Table 5 and an expanded comprehensive table containing pathways names and *p*-values in Supplemental Table 5. In the top 30 pathways identified, most were involved in immunity, including adaptive immunity, cytokine signaling, and infectious diseases. Collectively, these pathways were composed largely of proteins focused on antigen processing and presentation. Pathways involved in extracellular matrix organization were the sole non-immune members in the top 30 overrepresented group.

**Table 5:** Significantly overrepresented cellular pathways in IIM

Pathway category	Submitted entities found
Adaptive immune system	HLA-DRA; HLA-DRB1; HLA-A; HLA-B; HLA-C; HLA-H; TAP1; B2M; FGB; S100A1; FGG; COL1A1; C3; COL3A1; COL1A2
Cytokine signaling in immune system	HLA-DRA; HLA-DRB1; HLA-A; HLA-B; HLA-C; HLA-H; B2M; ANXA1; COL1A2; CA2; MSN
Infectious disease	HLA-A; HLA-B; HLA-C; HLA-H; B2M; ATP1A1; FGB; GGT5; S100A1; FGG; BGN; APOA1; MSN; DCN; GNAI2; C3; MYO1C; MYH9
Extracellular matrix organization	COL1A1; COL3A1; COL1A2; COL4A1; COL6A2; COL6A1; BGN; DCN; FGB; FGG; CMA1
Vesicle-mediated transport	COL1A1; COL3A1; COL1A2; COL4A1; APOA1; IGHA1
Metabolism	CA2; RHD; BGN; DCN
Nervous system development	COL3A1; COL4A1; COL6A2; COL6A1
Innate immune system	HLA-B; HLA-C; B2M; FGB; S100A1; FGG
Hemostasis	COL1A1; FGB; COL1A2; FGG
Diseases associated with the TLR signaling cascade	FGB; S100A1; FGG
Diseases associated with glycosaminoglycan metabolism	BGN; DCN
Signal transduction	COL1A1; COL3A1; COL1A2; COL4A1; COL6A2; COL6A1
Sensory perception	MYO1C; MYH9; MSN; EZR
Other: immune system	FGB; HLA-H; ANXA1; S100A1; FGG; HLA-B; HLA-C; MSN; TAP1; HLA-A; A1BG; COL1A1; C3; COL3A1; COL1A2; MYO1C; CA2; HLA-DRA; MYH9; ACAA1; B2M; HLA-DRB1

Further examination expanded the network of pathways that are affected by changes in collagen proteins, in addition to extracellular matrix organization. This includes pathways involved in vesicle-mediated transport, nervous system development, hemostasis, and signal transduction (MET and PDGF signaling). Pathways involved in metabolism, diseases associated with TLR signaling, and sensory perception were also significantly overrepresented in IIM patients.

**Fig. 7:**
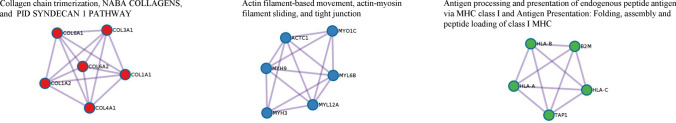
Overrepresented physically Interacting Protein Networks. Dysregulated proteins that physically interact with one another are visualized in networks. Lines show which proteins within the network are physically complexed with one another

Top overrepresented physically interacting protein networks in IIM included collagens specific to collagen chain trimerization and associated syndecan 1 signaling, which may function in cellular proliferation, migration, and repair of damaged muscle (Fig. 7). Collagens were also prominent in our broader analysis of overrepresented proteins (Table 5 and Supplemental Table 5). This physically interacting network analysis adds to our knowledge by showing which of the overexpressed collagens bundle together in IIM muscle. Additionally, interacting proteins involved in muscle and non-muscle cellular movement, trafficking and cell adhesion were upregulated. As expected, complexes related to immune function (antigen processing and presentation) were also upregulated in IIM muscle relative to control.

Together, these exploratory data build on our current knowledge of dysregulated proteins and their affected pathways in IIM with mitochondrial abnormalities and provide a resource for future investigators when interrogating perturbed pathways and proteins in IIM.

### Localization of differentially expressed proteins in IIM muscle

We next selected biopsies from a separate cohort of IIM subjects to localize proteins identified as differentially expressed from control subjects in this study. Subject characteristics are reported in Supplementary Table 6. MHC class I, COPS7A (CSN7A; COP9 signalosome subunit 7A), RAB7A (Ras-related protein Rab-7a), and HSP70-1 (HSPA1A; Heat shock protein family A (Hsp70) member 1A) were chosen for localization. MHC class I was overrepresented in IIM patients, while COPS7A, RAB7A, and HSP70-1 were underrepresented in IIM patients compared to controls.

**Fig. 8:**
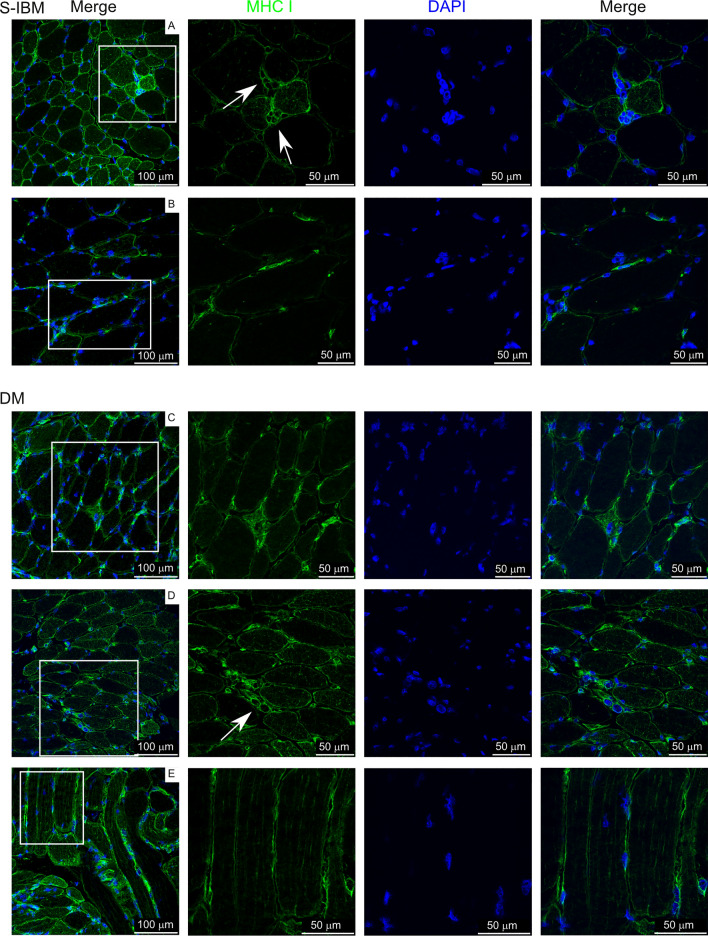
MHC class I labeling on IIM muscle biopsies. White box in left panels indicates area amplified in panels to the right. Arrows indicate staining of interest discussed in the text

Figure 8 shows detection of MHC class I protein, which is important in antigen processing and presentation (as described with Fig. 5). MHC class I protein was detected on the membranes of many muscle fibers as expected. Additionally, expression was also localized to small cells adjacent to mature muscle fibers, which appear to be regenerating myofibers (panels A, D labeled with arrows).

**Fig. 9:**
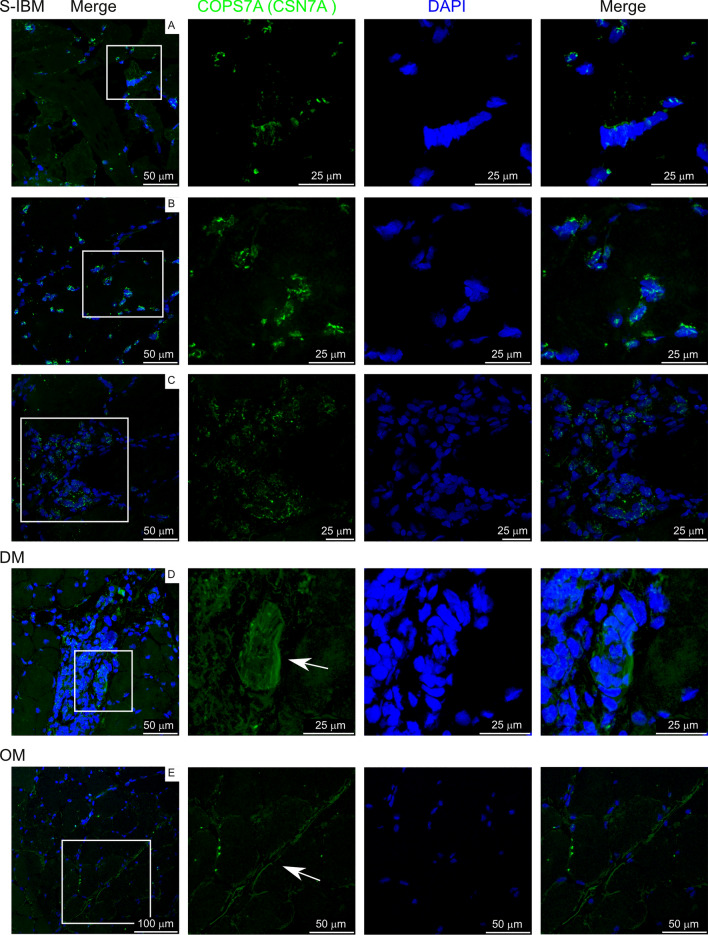
COPS7A (CSN7A) labeling on IIM muscle biopsies. White box in left panels indicates area amplified in panels to the right. Arrows indicate staining of interest discussed in the text

Figure 9 shows detection of CSN7A staining in subjects. This protein is a component of the COP9 (constitutive photomorphogenesis 9) signalosome, a diverse signaling complex involved in processes such as cell proliferation, DNA damage repair, cell cycle, metabolism, and inflammation [35]. CSN7A was localized to select nuclei within our muscle biopsies, but also showed some cytoplasmic staining. Additionally, labeling was localized to structures that resemble a neutrophil net (panel D, labeled with arrow) and occasional cell membranes (panel E, labeled with arrow).

**Fig. 10:**
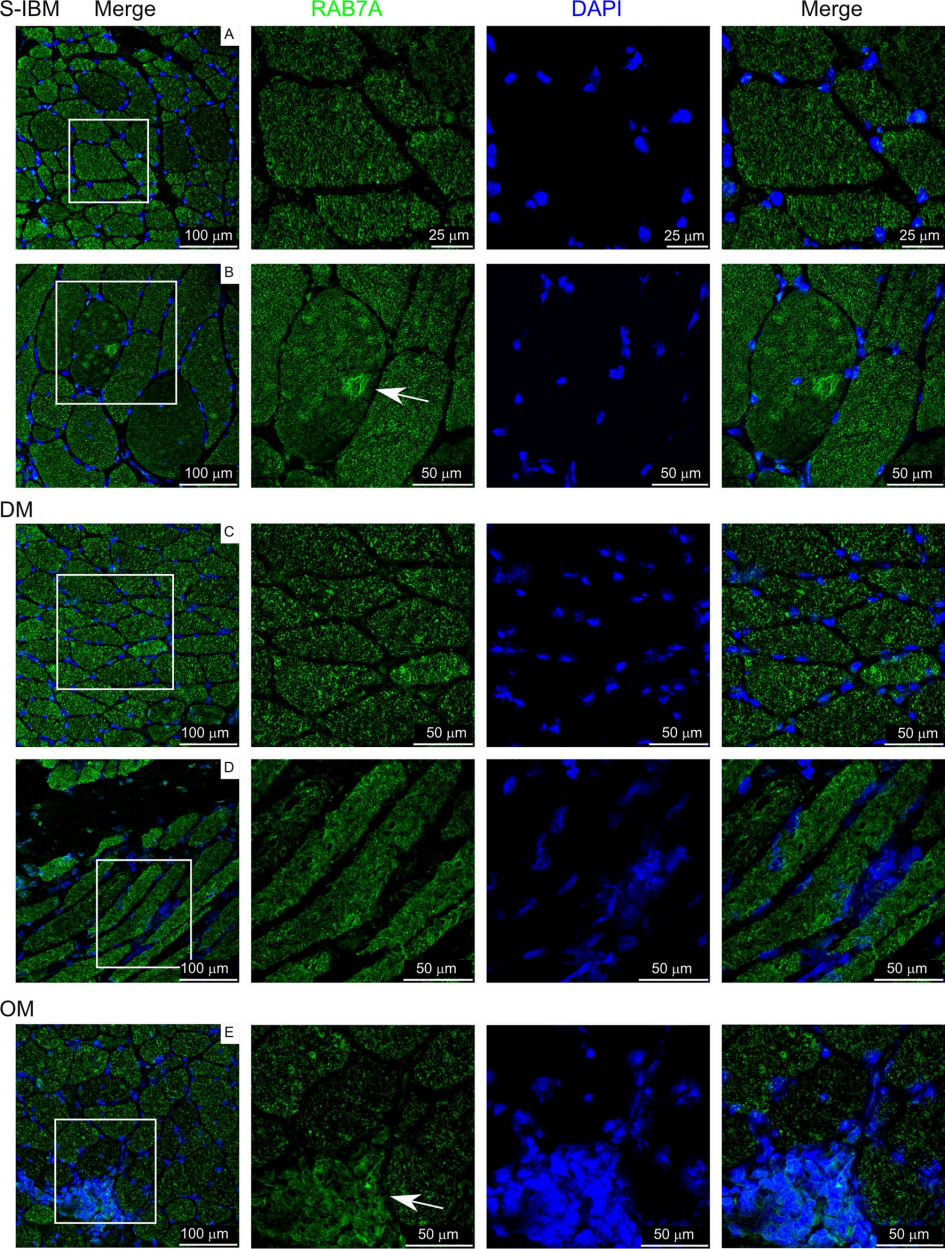
RAB7A labeling on IIM muscle biopsies. White box in left panels indicates area amplified in panels to the right. Arrows indicate staining of interest discussed in the text

Figure 10 shows detection of RAB7A staining. As a protein involved in vesicular trafficking, it plays roles in endocytosis, late endosome trafficking, autophagy, mitophagy, and lipophagy [36]. RAB7A localized to structures within cells, likely endosomes/lysosomes with varying intensities between myofibers. Concentrated expression was detected in rimmed vacuoles from sIBM samples (panel B labeled with arrow). RAB7A was also detected in regions of cellular infiltration (panel E labeled with arrow).

**Fig. 11:**
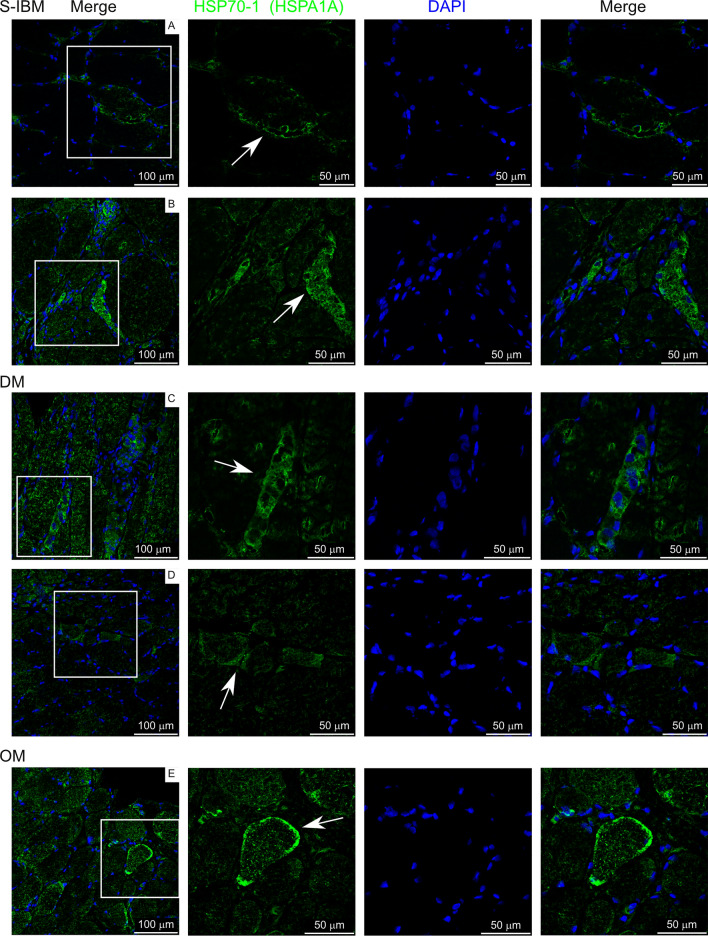
HSP-70-1 (HSPA1A) labeling on IIM muscle biopsies. White box in left panels indicates area amplified in panels to the right. Arrows indicate staining of interest discussed in the text

Figure 11 shows detection of HSP70-1. This protein is involved in cellular homeostasis and the heat shock response, trafficking damaged proteins to lysosomes and stabilizing lysosomal membranes [37]. HSP70-1 was found absent on most myofibers within muscle biopsies. As an inducible protein associated with stress, intracellular HSP70-1 was detected most prominently in myofibers that were invaded by inflammatory cells (panels B, C labeled with arrows). There were, however, some myofibers where inflammatory cells presence was not visible, but peripheral/sarcolemmal HSP70-1 labeling occurred (panels A, D, E labeled with arrows).

### Visualizing subcellular localization of differentially expressed proteins in IIM muscle

**Fig. 12:**
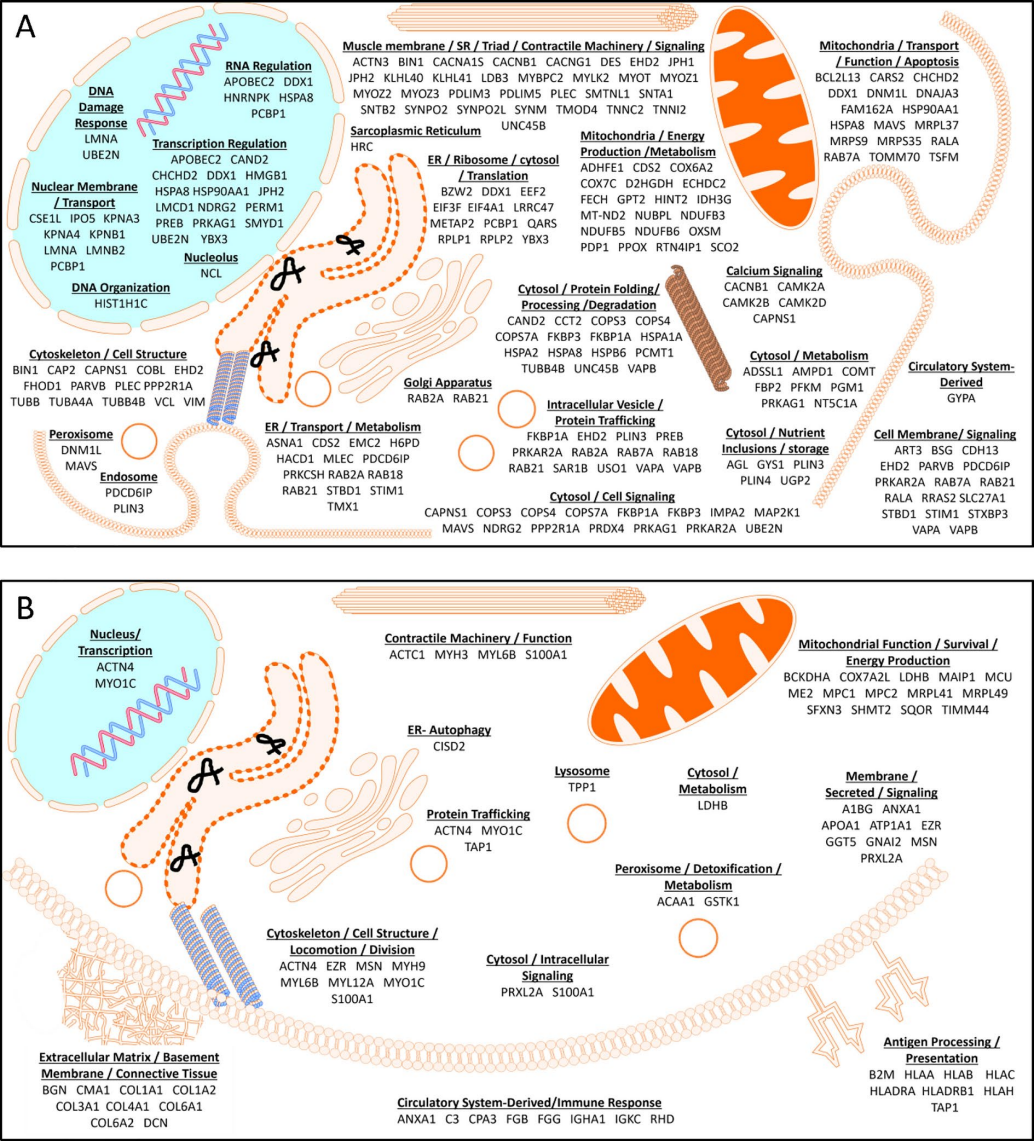
Cartoons representing proteins differentially regulated in IIM muscle. **A** Underrepresented proteins and **B** Overrepresented proteins in IIM compared to control muscle

Figure 12A, B provides a pictorial representation of the differentially expressed proteins and the cellular compartments where they function using data from Supplemental Table 4 (fold change 2.83 or greater). The majority of differentially expressed proteins were underrepresented in IIM (168 of 228; 73.7%). Four of the 228 proteins are not represented in the figure because their functions are uncertain. This highlights the widespread dysregulation occurring in IIM muscle, as every organelle is affected. Consistent with pathway analysis, affected proteins are involved in cell maintenance, contractile function, and signaling, providing a comprehensive picture of the dysregulation captured from the isolation of membrane-bound organelles from muscle biopsies.

## Discussion

We performed this study to explore the utility of subcellular proteomics as a means of better understanding the pathophysiology of IIM with mitochondrial abnormalities. Our data show that by isolating and analyzing the membrane-bound organelle fraction of muscle biopsies, IIM patient muscle expresses a distinctly different proteome than control muscle.

Mitochondrial abnormalities were characteristic of subject’s biopsies in this study, we therefore interrogated dysregulated mitochondrial proteins in our analysis. Thirteen mitochondrial inner membrane proteins were found to be dysregulated in myositis patients compared to controls (Fig. 4). Seven were directly involved in energy production through the mitochondrial electron transport chain. This includes three subunits of respiratory complex I (NDUFB3, NDUFB5, MT-ND2) three subunits of complex IV/COX (COX6A2, COX7A2L, COX7C), and a COX assembly factor (SCO2). Mitochondrial DNA deletions, duplications, and an overall reduction in mitochondrial DNA copy number have been previously identified in IIM patients with COX-deficient myofibers [25–27]. Consistent with our observations, complex I and IV deficiencies were identified [25]. It is unknown whether our cohort of patients have mutations or protein expression is reduced for other reasons, but the commonality of electron transport chain deficits phenotypically links these subjects.

Pathway analysis is a helpful tool for organizing data into categories to understand how disease disrupts cellular function. Even with this organization, data interpretation is challenging because many proteins are diverse, functioning within multiple pathways, potentially resulting in perturbation of multiple processes within an organ. Adding additional complexity, IIM encompasses a heterogenous population, resulting in some but not all subjects showing similar protein changes (as illustrated with mitochondrial electron transport chain protein expression; Fig. 4). Our initial analysis was beneficial for distinguishing IIM with mitochondrial abnormalities from control and a mitochondrial myopathy subject (Fig. 2), but the statistical cutoffs proved too stringent for discovering novel proteins/pathways to interrogate with this heterogenous IIM population, as pathways and proteins that were revealed have previously been well described as dysregulated in IIM (Table 2). Using a less stringent cutoff, we re-analyzed the data, grouped similar pathways to reduce the complexity (Tables 3 and 4), and interrogated multiple pathway tools to search for less recognized but robust pathways/proteins that showed up in these searches (Figs. 6, 7 and Tables 3, 4). Identification of these specific over and underexpressed proteins and visualization of the different pathways they affect may help direct future studies aimed at understanding mechanisms of muscle dysfunction in IIM and choosing downstream functions that may be improved when perturbed protein expression is corrected.

Proteomics via mass spectrometry is a quantitative method we used in this study to address differential expression of proteins within membrane-bound organelles. As a complimentary technique, we chose immunolabeling, a non-quantitative technique to visualize the spatial localization of select dysregulated proteins. We focused on four proteins, choosing those that were prominent in our pathway analyses, as these proteins are more likely to contribute to cellular dysfunction and therefore pathology in IIM. MHC class I (HLA proteins) were overrepresented in IIM subjects, while RAB7A, CSN7A, and HSP70-1 were underexpressed. This representation reflects that three-forths of dysregulated proteins were underexpressed in this study. HLA, CSN7A and HSP70-1 were identified in our physically interacting protein networks (Figs. 6 and 7) and all 4 were identified in subsequent pathway analyses (Tables 4 and 5 and Supplemental Tables 4 and 5). CSN7A has not previously been identified as dysregulated in any IIM subtypes and therefore represents a novel finding. HLA proteins, RAB7A, and HSP70-1 have been previously identified as differentialy expressed in IIM, however RAB7A and HSP70-1 have been studied in sIBM but not DM, PM, or OM. Therefore our interest in RAB7A and HSP70-1 is to highlight the possibility that these proteins and their associated pathways may be more widely involved in mediating IIM pathology, potentially extrapolated to IIM patients experiencing mitochondrial abnormalties.

RAB7A was previously reported as present and overrepresented specifically within sIBM patient rimmed vacuoles [8], but expression outside of rimmed vacules had not previously been addressed. RAB7A localization in our study also found concentrated surrounding rimmed vacuoles of sIBM biopsies, but when all membrane-bound organelles were included in the analysis, this protein was found to be underexpressed compared to control biopsies in all IIM patients except OM-12. RAB7A is a diverse protein, in addition to being involved in protein trafficking in and through cells, it is involved in autophagy, including specific types of autophagy such as mitophagy and lipophagy, apoptosis including ferroptosis, and cytoskeletal functions [36, 38]. Therefore, protein localization may provide insight into how imbalanced homeostasis is affecting IIM patient muscle. For example, although RAB7A may be concentrated in rimmed vaccules of sIBM, its overall underexpression compared to control muscle in IIM with mitochondiral abnormalties may contribute to cellular dysruption due to its unavailability to be involved in its other vital cellular processes such as mitophagy.

Histological hallmarks of sIBM include abnormal muscle inclusions and rimmed vacuoles. Much research on sIBM has focused on identifying dysregulated proteostasis processes such as chaperone-mediated autophagy and mitophagy because they can lead to accumulation of misfolded and postranslationally modified proteins resulting in the presence of inclusions and rimmed vacuoles [39]. Recent studies indicate the presence of vacuoles with chaperone-mediated abnormalties in immune‐mediated necrotizing myopathy as well [40, 41]. Heat shock proteins are molecular chaperones important for folding of newly synthesized proteins as well as responding to stress-induced unfolded protein response. A global cellular response is needed in attempt to maintain a functional proteome during cell stress. This type of response includes an integrated stress response aimed at reducing global protein synsthesis in favor of specific stress associated protein synthesis to help regain proteostasis and unfolded protein and heat shock responses to remove misfolded and damaged proteins from the cell [42]. Upregulation of heat shock proteins using the drug arimoclomol has been investigated as a potential therapy for sIBM in attempt to reduce protein aggregation in muscle [43, 44]. Although insufficient clinical benefit was found using arimoclomol as a monotherapy, this does not negate that dysregulation of this cellular maintenance and stress response system negatively impacts IIM muscle health. Instead, it may indicate that multiple interlinked cell stress pathways may need to be targeted to attain clinical benefit. Our study shows HSP70-1 localization was found predominatly in areas of inflammatory cell infiltration. Interestingly, concentrated HSP70-1 was also found on the periphery of select fibers. Membrane HSP70-1 is well know for its role in protein folding and refolding, but peripheral localization has been detected and described in cancer conditions and in the context of aiding antigen presentation [45–47]. Intriguingly, exosomal release of HSP70-1 from dying cancer cells has been shown in the short-term to result in hightened immune responses, whereas long-term exposure induces immune tolerance [45]. It is unknown whether membrane HSP70-1 in these IIM patients results in exosomal release of this protein from muscle and what affect this may have on long-term immune responses in IIM patients.

CSN7A localized to select nuclei within muscle, occasionally to cell membranes, and structures within inflamed areas that resemble neutrophil nets, but was otherwise expressed at low levels within biopsies. CSN7A as well as several other proteins involved in regulation of ubiquitination were identified as underexpressed in our analysis, including other members of the COP9 signalosome (COPS3, COPS4) and KLHL41 (Kelch-like family member 41). The COP9 signalosome deneddylates Cullin proteins, which destabilizes the complex limiting its ubiquitin ligase activity [35]. Therefore, underexpression of COPS proteins leads to enhanced substrate ubiquitination through this pathway. Downregulation of any of the COPS protein members results in destabilization of the entire complex, revealing the importance of proper regulation of this pathway. In mice, cardiac specific knockout of COPS5 disrupts the ubiquitin–proteasome pathway, resulting in impaired autophagy and accumulation of misfolded protein aggregates. This leads to cardiomyocyte necrosis, inflammatory cell infiltration and early lethality [48–51]. In an ischemic brain model of disease, impairing COP9 signalosome activity through perturbation of COPS5 promotes neuroinflammation, whereas enhancing COP9 signalosome activity protects against neuroinflammation and stress-induced neuronal cell death [52]. This regulation of neuroinflammation is at least in part due to Cullin-mediated ubiquitination regulating NF-κB activation [35]. KLHL41, which interacts with Cullin3, stabilizes the skeletal muscle scaffolding protein nebulin through polyubiquitination, thereby being critical for sarcomeric stabilization [53]. Together these studies show that reduced deneddylation alters the balance of Cullin-induced ubiquitination, perturbing cellular homeostasis and leading to inflammation and protein aggregates, characteristic features of IIM. Further, reduced stabilization of sarcomeres could further disrupt muscle homeostasis contributing to pathological features observed in IIM.

Together, RAB7A, CSN7A, and HSP70-1 are involved in cellular stress responses, mediating protein traficking important for managing the unfolded protein response and mitophagy, and mobilizing MHC molecules to the cell surface. Dysregulation of mitophagy leads to dysfunctional mitochondria and results in increased oxidative damage to cellular proteins. Oxidative damage to HSP70-1 impairs its ability to function properly [37], preventing a return to proteostasis and promoting further cellular dysfunction. Therefore, dysregulation of these proteins and their respective pathways provide a potential link to mitochondrial dysfunction in these patients.

In summary, this study identifies proteins intersecting a wide range of homeostatic pathways that are dysregulated in IIM muscles with mitochondrial abnormalities. Although we highlighted a few of these proteins, research will need to continue into the array of dysregulated proteins and their contribution to various pathological features of disease.

## Supplementary Information

Below is the link to the electronic supplementary material.

Supplementary file 1 (PDF 610 KB)

## Data Availability

Data is provided within the manuscript or supplementary information files.

## References

[1] Varone N, Hinojosa J, Nandakumar D, Modi N, Bhashyam AR, Bhai SF. Exercise recommendations for patients with myositis: a narrative review of safety and efficacy. Clin Exp Rheumatol. 2024;42(2):436–44. 38436327 10.55563/clinexprheumatol/m8fbs1

[2] Coffey VG, Hawley JA. The molecular bases of training adaptation. Sports Med. 2007;37(9):737–63. 17722947 10.2165/00007256-200737090-00001

[3] Chatzinikita E, Maridaki M, Palikaras K, Koutsilieris M, Philippou A. The role of mitophagy in skeletal muscle damage and regeneration. Cells. 2023;12(5):716. 36899852 10.3390/cells12050716 PMC10000750

[4] Schroder JM, Molnar M. Mitochondrial abnormalities and peripheral neuropathy in inflammatory myopathy, especially inclusion body myositis. Mol Cell Biochem. 1997;174(1–2):277–81. 9309700

[5] Danieli MG, Antonelli E, Piga MA, Cozzi MF, Allegra A, Gangemi S. Oxidative stress, mitochondrial dysfunction, and respiratory chain enzyme defects in inflammatory myopathies. Autoimmun Rev. 2023;22(5): 103308. 36822387 10.1016/j.autrev.2023.103308

[6] Miller FW, Lamb JA, Schmidt J, Nagaraju K. Risk factors and disease mechanisms in myositis. Nat Rev Rheumatol. 2018;14(5):255–68. 29674613 10.1038/nrrheum.2018.48 PMC6745704

[7] Guttsches AK, Brady S, Krause K, Maerkens A, Uszkoreit J, Eisenacher M, et al. Proteomics of rimmed vacuoles define new risk allele in inclusion body myositis. Ann Neurol. 2017;81(2):227–39. 28009083 10.1002/ana.24847 PMC5323275

[8] Guttsches AK, Jacobsen F, Schreiner A, Mertens-Rill J, Tegenthoff M, Marcus K, et al. Chaperones in sporadic inclusion body myositis-Validation of proteomic data. Muscle Nerve. 2020;61(1):116–21. 31644823 10.1002/mus.26742

[9] Bhattacharya SK, Thakar JH, Johnson PL, Shanklin DR. Isolation of skeletal muscle mitochondria from hamsters using an ionic medium containing ethylenediaminetetraacetic acid and nagarse. Anal Biochem. 1991;192(2):344–9. 1903610 10.1016/0003-2697(91)90546-6

[10] Ramadasan-Nair R, Gayathri N, Mishra S, Sunitha B, Mythri RB, Nalini A, et al. Mitochondrial alterations and oxidative stress in an acute transient mouse model of muscle degeneration: implications for muscular dystrophy and related muscle pathologies. J Biol Chem. 2014;289(1):485–509. 24220031 10.1074/jbc.M113.493270 PMC3879571

[11] Abdullah HM, Higuchi I, Kubota R, Matsuura E, Hashiguchi A, Abdelbary NH, et al. Histopathological differences between human T-lymphotropic virus type 1-positive and human T-lymphotropic virus type 1-negative polymyositis. Clin Exp Neuroimmunol. 2011;2(1):12–24.

[12] De Bleecker JL, De Paepe B, Aronica E, de Visser M, Amato A, Group EMMBS, et al. 205th ENMC international workshop: pathology diagnosis of idiopathic inflammatory myopathies part II 28–30 march 2014, Naarden, The Netherlands. Neuromuscul Disord. 2015;25(3):268–72. 25572016 10.1016/j.nmd.2014.12.001

[13] Uhlen M, Fagerberg L, Hallstrom BM, Lindskog C, Oksvold P, Mardinoglu A, et al. Proteomics. Tissue-based map of the human proteome. Science. 2015;347(6220):1260419. 25613900 10.1126/science.1260419

[14] Renjini R, Gayathri N, Nalini A, Srinivas Bharath MM. Oxidative damage in muscular dystrophy correlates with the severity of the pathology: role of glutathione metabolism. Neurochem Res. 2012;37(4):885–98. 22219131 10.1007/s11064-011-0683-z

[15] Zybailov B, Mosley AL, Sardiu ME, Coleman MK, Florens L, Washburn MP. Statistical analysis of membrane proteome expression changes in Saccharomyces cerevisiae. J Proteome Res. 2006;5(9):2339–47. 16944946 10.1021/pr060161n

[16] Tyanova S, Temu T, Sinitcyn P, Carlson A, Hein MY, Geiger T, et al. The Perseus computational platform for comprehensive analysis of (prote)omics data. Nat Methods. 2016;13(9):731–40. 27348712 10.1038/nmeth.3901

[17] Chen J, Bardes EE, Aronow BJ, Jegga AG. ToppGene Suite for gene list enrichment analysis and candidate gene prioritization. Nucleic Acids Res. 2009;37(Web Server issue):W305–11. 19465376 10.1093/nar/gkp427 PMC2703978

[18] Liebermeister W, Noor E, Flamholz A, Davidi D, Bernhardt J, Milo R. Visual account of protein investment in cellular functions. Proc Natl Acad Sci U S A. 2014;111(23):8488–93. 24889604 10.1073/pnas.1314810111 PMC4060655

[19] Gillespie M, Jassal B, Stephan R, Milacic M, Rothfels K, Senff-Ribeiro A, et al. The reactome pathway knowledgebase 2022. Nucleic Acids Res. 2022;50(D1):D687–92. 34788843 10.1093/nar/gkab1028 PMC8689983

[20] Zhou Y, Zhou B, Pache L, Chang M, Khodabakhshi AH, Tanaseichuk O, et al. Metascape provides a biologist-oriented resource for the analysis of systems-level datasets. Nat Commun. 2019;10(1):1523. 30944313 10.1038/s41467-019-09234-6 PMC6447622

[21] Amlani A, Choi MY, Tarnopolsky M, Brady L, Clarke AE, La Garcia-De TI, et al. Anti-NT5c1A autoantibodies as biomarkers in inclusion body myositis. Front Immunol. 2019;10:745. 31024569 10.3389/fimmu.2019.00745 PMC6465553

[22] Ikenaga C, Findlay AR, Goyal NA, Robinson S, Cauchi J, Hussain Y, et al. Clinical utility of anti-cytosolic 5′-nucleotidase 1A antibody in idiopathic inflammatory myopathies. Ann Clin Transl Neurol. 2021;8(3):571–8. 33556224 10.1002/acn3.51294 PMC7951108

[23] Lee SA, Lee HJ, Suh BC, Shin HY, Kim SW, Yoon BA, et al. Clinical significance of anti-NT5c1A autoantibody in Korean patients with inflammatory myopathies. PLoS ONE. 2023;18(4): e0284409. 37058449 10.1371/journal.pone.0284409 PMC10104319

[24] Lilleker JB, Rietveld A, Pye SR, Mariampillai K, Benveniste O, Peeters MT, et al. Cytosolic 5′-nucleotidase 1A autoantibody profile and clinical characteristics in inclusion body myositis. Ann Rheum Dis. 2017;76(5):862–8. 28122761 10.1136/annrheumdis-2016-210282 PMC5530338

[25] Hedberg-Oldfors C, Lindgren U, Basu S, Visuttijai K, Lindberg C, Falkenberg M, et al. Mitochondrial DNA variants in inclusion body myositis characterized by deep sequencing. Brain Pathol. 2021;31(3): e12931. 33354847 10.1111/bpa.12931 PMC8412083

[26] Oldfors A, Larsson NG, Lindberg C, Holme E. Mitochondrial DNA deletions in inclusion body myositis. Brain. 1993;116(Pt 2):325–36. 8384916 10.1093/brain/116.2.325

[27] Blume G, Pestronk A, Frank B, Johns DR. Polymyositis with cytochrome oxidase negative muscle fibres. Early quadriceps weakness and poor response to immunosuppressive therapy. Brain. 1997;120(Pt 1):39–45. 9055796 10.1093/brain/120.1.39

[28] Coley W, Rayavarapu S, Nagaraju K. Role of non-immune mechanisms of muscle damage in idiopathic inflammatory myopathies. Arthritis Res Ther. 2012;14(2):209. 22546362 10.1186/ar3791 PMC3446443

[29] Rayavarapu S, Coley W, Kinder TB, Nagaraju K. Idiopathic inflammatory myopathies: pathogenic mechanisms of muscle weakness. Skelet Muscle. 2013;3(1):13. 23758833 10.1186/2044-5040-3-13 PMC3681571

[30] Tezak Z, Hoffman EP, Lutz JL, Fedczyna TO, Stephan D, Bremer EG, et al. Gene expression profiling in DQA1*0501+ children with untreated dermatomyositis: a novel model of pathogenesis. J Immunol. 2002;168(8):4154–63. 11937576 10.4049/jimmunol.168.8.4154

[31] Coley W, Rayavarapu S, Pandey GS, Sabina RL, Van der Meulen JH, Ampong B, et al. The molecular basis of skeletal muscle weakness in a mouse model of inflammatory myopathy. Arthritis Rheum. 2012;64(11):3750–9. 22806328 10.1002/art.34625 PMC3485437

[32] Fishbein WN. Myoadenylate deaminase deficiency: inherited and acquired forms. Biochem Med. 1985;33(2):158–69. 4004819 10.1016/0006-2944(85)90024-9

[33] Sabina RL, Fishbein WN, Pezeshkpour G, Clarke PR, Holmes EW. Molecular analysis of the myoadenylate deaminase deficiencies. Neurology. 1992;42(1):170–9. 1370861 10.1212/wnl.42.1.170

[34] Sabina RL, Sulaiman AR, Wortmann RL. Molecular analysis of acquired myoadenylate deaminase deficiency in polymyositis (idiopathic inflammatory myopathy). Adv Exp Med Biol. 1991;309B:203–5. 1781368 10.1007/978-1-4615-7703-4_46

[35] Schulze-Niemand E, Naumann M. The COP9 signalosome: a versatile regulatory hub of Cullin-RING ligases. Trends Biochem Sci. 2023;48(1):82–95. 36041947 10.1016/j.tibs.2022.08.003

[36] Guerra F, Bucci C. Multiple roles of the small GTPase Rab7. Cells. 2016;5(3):34. 27548222 10.3390/cells5030034 PMC5040976

[37] Yamashima T, Mochly-Rosen D, Wakatsuki S, Mizukoshi E, Seike T, Larus IM, et al. Cleavage of Hsp70.1 causes lysosomal cell death under stress conditions. Front Mol Biosci. 2024;11:1378656. 38859931 10.3389/fmolb.2024.1378656 PMC11163108

[38] Xie Y, Zhou Y, Wang J, Du L, Ren Y, Liu F. Ferroptosis, autophagy, tumor and immunity. Heliyon. 2023;9(9): e19799. 37810047 10.1016/j.heliyon.2023.e19799 PMC10559173

[39] Askanas V, Engel WK, Nogalska A. Sporadic inclusion-body myositis: a degenerative muscle disease associated with aging, impaired muscle protein homeostasis and abnormal mitophagy. Biochim Biophys Acta. 2015;1852(4):633–43. 25241263 10.1016/j.bbadis.2014.09.005

[40] Fischer N, Preusse C, Radke J, Pehl D, Allenbach Y, Schneider U, et al. Sequestosome-1 (p62) expression reveals chaperone-assisted selective autophagy in immune-mediated necrotizing myopathies. Brain Pathol. 2020;30(2):261–71. 31376301 10.1111/bpa.12772 PMC8018061

[41] Preusse C, Marteau T, Fischer N, Hentschel A, Sickmann A, Lang S, et al. Endoplasmic reticulum-stress and unfolded protein response-activation in immune-mediated necrotizing myopathy. Brain Pathol. 2022;32(6): e13084. 35703068 10.1111/bpa.13084 PMC9616093

[42] Kohler A, Kohler V. Better together: interorganellar communication in the regulation of proteostasis. Contact (Thousand Oaks). 2024;7:25152564241272244. 39385949 10.1177/25152564241272245 PMC11462569

[43] Ahmed M, Machado PM, Miller A, Spicer C, Herbelin L, He J, et al. Targeting protein homeostasis in sporadic inclusion body myositis. Sci Transl Med. 2016;8(331):331ra41. 27009270 10.1126/scitranslmed.aad4583 PMC5043094

[44] Machado PM, McDermott MP, Blaettler T, Sundgreen C, Amato AA, Ciafaloni E, et al. Safety and efficacy of arimoclomol for inclusion body myositis: a multicentre, randomised, double-blind, placebo-controlled trial. Lancet Neurol. 2023;22(10):900–11. 37739573 10.1016/S1474-4422(23)00275-2

[45] Albakova Z, Armeev GA, Kanevskiy LM, Kovalenko EI, Sapozhnikov AM. HSP70 multi-functionality in cancer. Cells. 2020;9(3):587. 32121660 10.3390/cells9030587 PMC7140411

[46] Shevtsov M, Multhoff G. Heat shock protein-peptide and hsp-based immunotherapies for the treatment of cancer. Front Immunol. 2016;7:171. 27199993 10.3389/fimmu.2016.00171 PMC4850156

[47] Udono H, Ichiyanagi T, Mizukami S, Imai T. Heat shock proteins in antigen trafficking–implications on antigen presentation to T cells. Int J Hyperthermia. 2009;25(8):617–25. 19551545 10.3109/02656730902902183

[48] Su H, Li F, Ranek MJ, Wei N, Wang X. COP9 signalosome regulates autophagosome maturation. Circulation. 2011;124(19):2117–28. 21986281 10.1161/CIRCULATIONAHA.111.048934 PMC3211066

[49] Su H, Li J, Menon S, Liu J, Kumarapeli AR, Wei N, et al. Perturbation of cullin deneddylation via conditional Csn8 ablation impairs the ubiquitin-proteasome system and causes cardiomyocyte necrosis and dilated cardiomyopathy in mice. Circ Res. 2011;108(1):40–50. 21051661 10.1161/CIRCRESAHA.110.230607 PMC3017673

[50] Su H, Li J, Osinska H, Li F, Robbins J, Liu J, et al. The COP9 signalosome is required for autophagy, proteasome-mediated proteolysis, and cardiomyocyte survival in adult mice. Circ Heart Fail. 2013;6(5):1049–57. 23873473 10.1161/CIRCHEARTFAILURE.113.000338 PMC3835345

[51] Su H, Li J, Zhang H, Ma W, Wei N, Liu J, et al. COP9 signalosome controls the degradation of cytosolic misfolded proteins and protects against cardiac proteotoxicity. Circ Res. 2015;117(11):956–66. 26383969 10.1161/CIRCRESAHA.115.306783 PMC4636927

[52] Tian Y, Milic J, Monasor LS, Chakraborty R, Wang S, Yuan Y, et al. The COP9 signalosome reduces neuroinflammation and attenuates ischemic neuronal stress in organotypic brain slice culture model. Cell Mol Life Sci. 2023;80(9):262. 37597109 10.1007/s00018-023-04911-8 PMC10439869

[53] Ramirez-Martinez A, Cenik BK, Bezprozvannaya S, Chen B, Bassel-Duby R, Liu N, Olson EN. KLHL41 stabilizes skeletal muscle sarcomeres by nonproteolytic ubiquitination. Elife. 2017. 10.7554/eLife.26439. 28826497 10.7554/eLife.26439 PMC5589419

