# Supplementary material for: A window into intracellular events in myositis through subcellular proteomics
Source: Inflamm Res. 2025 Jan 31;74(1):31. doi: 10.1007/s00011-025-01996-8 (PMC11785624; doi:10.1007/s00011-025-01996-8)
Supplement: Supplementary file 1 — (PDF 610 KB) [file 11_2025_1996_MOESM1_ESM.pdf]

Supplementary Tables

Title : A window into intracellular events in myositis through subcellular proteomics

Journal : Inflammation Research

Jennifer M. Peterson[1*], Valérie Leclair[2], Olumide E. Oyebode[1], Dema M. Herzallah[1], Andrea L. Nestor-Kalinoski[3] Jose Morais[4], René P. Zahedi[5,6,7], Mazen Alamr[8], John A. DiBattista[9], Marie Hudson[2*]

[*1]Department of Exercise & Rehabilitative Sciences, The University of Toledo, Toledo OH, USA, [2]Division of Rheumatology and Department of Medicine, McGill University, Montreal, QC, Canada, [3]Advanced Microscopy & Imaging Center, Department of Surgery, University of Toledo, Toledo, OH, [4]Division of Geriatric Medicine and Research Institute, McGill University Health Centre, Quebec, Montreal, QC, Canada, [5]Segal Cancer Proteomics Centre, Lady Davis Institute for Medical Research, Montreal, QC, Canada, [6]Manitoba Centre for Proteomics and Systems Biology, University of Manitoba, Winnipeg, MB, Canada, [7]Department of Internal Medicine and Department of Biochemistry and Medical Genetics, University of Manitoba, Winnipeg, MB, Canada, [8]Department of Neurology and Neurosurgery, McGill University, Montreal, QC, Canada, [9]Department of Medicine and Experimental Medicine, McGill University Health Centre Research Institute, Montreal, QC, Canada.

Corresponding authors*.

Address Correspondence To: Jennifer M. Peterson, The University of Toledo, 2801 W. Bancroft St., MS 119, Toledo OH, 43606, phone: 419-530-6679, email: jennifer.peterson@utoledo.edu

Supplemental Table 1 Page 1. All proteins quantitated in study

Protein Name	Uniprot	Gene	Subcellular localization
T-complex protein 1 subunit beta	P78371	CCT2	Cytoplasm {ECO:0000269|PubMed:20080638}.
Importin subunit beta-1	Q14974	KPNB1	Cytoplasm {ECO:0000269|PubMed:11891849}. Nucleus envelope {ECO:0000269|PubMed:11891849}.
Phosphoglucomutase-1	P36871	PGM1	Isoform 1: Cytoplasm.
Cytochrome c oxidase subunit 7C, mitochondrial	P15954	COX7C	Mitochondrion inner membrane {ECO:0000250}.
Heat shock cognate 71 kDa protein	P11142	HSPA8	Cytoplasm. Melanosome. Nucleus, nucleolus. Cell membrane. Note=Localized in cytoplasmic mRNP granules containing untranslated mRNAs. Translocates rapidly from the cytoplasm to the nuclei, and especially to the nucleoli, upon heat shock.
LIM domain-binding protein 3	O75112	LDB3	Cytoplasm, perinuclear region {ECO:0000269|PubMed:10427098}. Cell projection, pseudopodium {ECO:0000269|PubMed:10427098}. Cytoplasm, cytoskeleton {ECO:0000269|PubMed:10427098}. Cytoplasm, myofibril, sarcomere, Z line {ECO:0000269|PubMed:10427098}. Note=Localized to the cytoplasm around nuclei and pseudopodia of undifferentiated cells and detected throughout the myotubes of differentiated cells. Colocalizes with ACTN2 at the Z-lines.
Putative HLA class I histocompatibility antigen, alpha chain H	P01893	HLA-H	Cell membrane {ECO:0000305}; Single-pass membrane protein {ECO:0000305}.
Adenylosuccinate synthetase isozyme 1	Q8N142	ADSSL1	Cytoplasm {ECO:0000255|HAMAP-Rule:MF_03126, ECO:0000269|PubMed:15786719}.
HLA class I histocompatibility antigen, B-49 alpha chain	P30487	HLA-B	Membrane; Single-pass type I membrane protein.
HLA class I histocompatibility antigen, B-40 alpha chain	Q04826	HLA-B	Membrane; Single-pass type I membrane protein.
Glycogen phosphorylase, muscle form	P11217	PYGM	0
Leucine-rich repeat-containing protein 47	Q8N1G4	LRRC47	0
Protein S100-A1	P23297	S100A1	Cytoplasm {ECO:0000269|PubMed:15171681}.
Cytochrome c oxidase subunit 7A1, mitochondrial	P24310	COX7A1	Mitochondrion inner membrane.
HLA class I histocompatibility antigen, A-1 alpha chain	P30443	HLA-A	Cell membrane {ECO:0000269|PubMed:25880248}; Single-pass type I membrane protein {ECO:0000255}. Endoplasmic reticulum membrane {ECO:0000250|UniProtKB:P01892}; Single-pass type I membrane protein {ECO:0000255}.
HLA class I histocompatibility antigen, B-8 alpha chain	P30460	HLA-B	Membrane; Single-pass type I membrane protein.

Supplemental Table 1 Page 2. All proteins quantitated in study

Protein Name	Uniprot	Gene	Subcellular localization
0	Q9HBI1	PARVB	Cell junction, focal adhesion. Cell membrane; Peripheral membrane protein; Cytoplasmic side. Cytoplasm, cytoskeleton. Cell projection, lamellipodium. Cytoplasm, myofibril, sarcomere. Cytoplasm, myofibril, sarcomere, Z line. Note=Constituent of focal adhesions. Detected at the tips of the leading edge of cells. Colocalizes with F-actin at the tips of lamellipodia.
HLA class I histocompatibility antigen, A-2 alpha chain	P01892	HLA-A	Cell membrane {ECO:0000269|PubMed:21263072, ECO:0000269|PubMed:8805302}; Single-pass type I membrane protein {ECO:0000255}. Endoplasmic reticulum membrane {ECO:0000305|PubMed:8805302}; Single-pass type I membrane protein {ECO:0000255}.
Beta-2-microglobulin	P61769	B2M	Secreted {ECO:0000269|PubMed:1336137, ECO:0000269|PubMed:7554280}. Cell surface {ECO:0000269|PubMed:25356553}. Note=Detected in serum and urine (PubMed:1336137, PubMed:7554280). {ECO:0000269|PubMed:7554280, ECO:0000269|Ref.6}.; Note=(Microbial infection) In the presence of M.tuberculosis EsxA-EsxB complex decreased amounts of B2M are found on the cell surface (PubMed:25356553). {ECO:0000269|PubMed:25356553}.
Phosphoglycerate kinase 1	P00558	PGK1	Cytoplasm.
Antigen peptide transporter 1	Q03518	TAP1	Endoplasmic reticulum membrane; Multi-pass membrane protein. Note=The transmembrane segments seem to form a pore in the membrane.
Up-regulated during skeletal muscle growth protein 5	Q96IX5	ATP5MD	Mitochondrion membrane {ECO:0000269|PubMed:21345788}; Single-pass membrane protein {ECO:0000255}.
Heat shock protein beta-6	O14558	HSPB6	Cytoplasm {ECO:0000269|PubMed:19464326}. Nucleus {ECO:0000269|PubMed:19464326}. Secreted {ECO:0000269|PubMed:22427880}. Note=Translocates to nuclear foci during heat shock. {ECO:0000269|PubMed:19464326}.
Tubulin alpha-4A chain	P68366	TUBA4A	Cytoplasm, cytoskeleton.
Ubiquinone biosynthesis monooxygenase COQ6, mitochondrial	Q9Y2Z9	COQ6	Mitochondrion inner membrane {ECO:0000255|HAMAP-Rule:MF_03193}; Peripheral membrane protein {ECO:0000255|HAMAP-Rule:MF_03193}; Matrix side {ECO:0000255|HAMAP-Rule:MF_03193}. Golgi apparatus {ECO:0000255|HAMAP-Rule:MF_03193}. Cell projection {ECO:0000255|HAMAP-Rule:MF_03193}. Note=Localizes to cell processes and Golgi apparatus in podocytes. {ECO:0000255|HAMAP-Rule:MF_03193}.
HLA class I histocompatibility antigen, B-7 alpha chain	P01889	HLA-B	Membrane; Single-pass type I membrane protein.
m-AAA protease-interacting protein 1, mitochondrial	Q8WWC4	MAIP1	Mitochondrion matrix {ECO:0000269|PubMed:27642048}.
COP9 signalosome complex subunit 7a	Q9UBW8	COPS7A	Cytoplasm {ECO:0000269|PubMed:9535219}. Nucleus {ECO:0000269|PubMed:9535219}.
NADH-cytochrome b5 reductase 3	P00387	CYB5R3	Isoform 1: Endoplasmic reticulum membrane; Lipid-anchor; Cytoplasmic side. Mitochondrion outer membrane; Lipid-anchor; Cytoplasmic side.; Isoform 2: Cytoplasm. Note=Produces the soluble form found in erythrocytes.
Adenylate kinase 2, mitochondrial	P54819	AK2	Mitochondrion intermembrane space {ECO:0000255|HAMAP-Rule:MF_03168}.

Supplemental Table 1 Page 3. All proteins quantitated in study

Protein Name	Uniprot	Gene	Subcellular localization
MICOS complex subunit MIC19	Q9NX63	CHCHD3	Mitochondrion inner membrane {ECO:0000250|UniProtKB:Q9CRB9}; Lipid-anchor {ECO:0000305}; Intermembrane side {ECO:0000250|UniProtKB:Q9CRB9}. Cytoplasm {ECO:0000269|PubMed:22567091}. Nucleus {ECO:0000269|PubMed:22567091}. Mitochondrion {ECO:0000269|PubMed:25781180, ECO:0000269|PubMed:25997101}.
Delta-sarcoglycan	Q92629	SGCD	Cell membrane, sarcolemma; Single-pass type II membrane protein. Cytoplasm, cytoskeleton.
HLA class II histocompatibility antigen, DRB1-1 beta chain	P04229	HLA-DRB1	Cell membrane {ECO:0000269|PubMed:18305173}; Single-pass type I membrane protein {ECO:0000269|PubMed:18305173}. Endoplasmic reticulum membrane {ECO:0000269|PubMed:18305173}; Single-pass type I membrane protein {ECO:0000269|PubMed:18305173}. Golgi apparatus, trans-Golgi network membrane {ECO:0000269|PubMed:18305173}; Single-pass type I membrane protein {ECO:0000269|PubMed:18305173}. Endosome membrane {ECO:0000269|PubMed:18305173}; Single-pass type I membrane protein {ECO:0000269|PubMed:18305173}. Lysosome membrane {ECO:0000269|PubMed:18305173}; Single-pass type I membrane protein {ECO:0000269|PubMed:18305173}. Late endosome membrane {ECO:0000269|PubMed:18305173}; Single-pass type I membrane protein {ECO:0000269|PubMed:18305173}. Note=The MHC class II complex transits through a number of intracellular compartments in the endocytic pathway until it reaches the cell membrane for antigen presentation.
Protein unc-45 homolog B	Q8IWX7	UNC45B	Cytoplasm, cytosol {ECO:0000250}.
HLA class I histocompatibility antigen, Cw-12 alpha chain	P30508	HLA-C	Membrane; Single-pass type I membrane protein.
ATP synthase subunit beta, mitochondrial	P06576	ATP5F1B	Mitochondrion inner membrane {ECO:0000250|UniProtKB:P00829}; Peripheral membrane protein {ECO:0000250|UniProtKB:P00829}; Matrix side {ECO:0000250|UniProtKB:P00829}.
Fructose-bisphosphate aldolase A	P04075	ALDOA	Cytoplasm, myofibril, sarcomere, I band. Cytoplasm, myofibril, sarcomere, M line. Note=In skeletal muscle, accumulates around the M line and within the I band, colocalizing with FBP2 on both sides of the Z line in the absence of Ca(2+). {ECO:0000250}.
Tripartite motif-containing protein 72	Q6ZMU5	TRIM72	Cell membrane, sarcolemma {ECO:0000250}. Cytoplasmic vesicle membrane {ECO:0000250}. Note=Tethered to plasma membrane and cytoplasmic vesicles via its interaction with phosphatidylserine. {ECO:0000250}.
39S ribosomal protein L49, mitochondrial	Q13405	MRPL49	Mitochondrion {ECO:0000269|PubMed:25278503, ECO:0000269|PubMed:25838379, ECO:0000269|PubMed:28892042}.
Apolipoprotein A-I	P02647	APOA1	Secreted.
Mitochondrial pyruvate carrier 2	O95563	MPC2	Mitochondrion inner membrane {ECO:0000269|PubMed:22628558}; Multi-pass membrane protein {ECO:0000269|PubMed:22628558}.

Supplemental Table 1 Page 4. All proteins quantitated in study

Protein Name	Uniprot	Gene	Subcellular localization
Calcium uniporter protein, mitochondrial	Q8NE86	MCU	Mitochondrion inner membrane {ECO:0000269|PubMed:21685886, ECO:0000269|PubMed:21685888, ECO:0000269|PubMed:23755363, ECO:0000269|PubMed:24231807, ECO:0000269|PubMed:27099988, ECO:0000305|PubMed:26341627}; Multi-pass membrane protein {ECO:0000269|PubMed:21685886, ECO:0000269|PubMed:21685888, ECO:0000269|PubMed:24231807}.
Apoptosis-inducing factor 1, mitochondrial	O95831	AIFM1	Mitochondrion intermembrane space. Mitochondrion inner membrane. Cytoplasm. Nucleus. Cytoplasm, perinuclear region. Note=Proteolytic cleavage during or just after translocation into the mitochondrial intermembrane space (IMS) results in the formation of an inner-membrane-anchored mature form (AIFmit). During apoptosis, further proteolytic processing leads to a mature form, which is confined to the mitochondrial IMS in a soluble form (AIFsol). AIFsol is released to the cytoplasm in response to specific death signals, and translocated to the nucleus, where it induces nuclear apoptosis. Colocalizes with EIF3G in the nucleus and perinuclear region.; Isoform 3: Mitochondrion intermembrane space {ECO:0000269|PubMed:20111043}. Mitochondrion inner membrane {ECO:0000269|PubMed:20111043}. Note=Has a stronger membrane anchorage than isoform 1.; Isoform 5: Cytoplasm {ECO:0000269|PubMed:16365034}.
Basigin	P35613	BSG	Cell membrane {ECO:0000269|PubMed:15946952, ECO:0000269|PubMed:17127621, ECO:0000269|PubMed:21536654}; Single-pass type I membrane protein {ECO:0000303|PubMed:15946952}. Melanosome {ECO:0000269|PubMed:17081065}. Note=Identified by mass spectrometry in melanosome fractions from stage I to stage IV. In spermatozoa, localized on the principal piece of caput and in the middle piece during transit in the corpus and cauda epididymides (By similarity). {ECO:0000250|UniProtKB:P18572, ECO:0000269|PubMed:17081065}.
Complement C3	P01024	C3	Secreted.
ATP synthase subunit e, mitochondrial	P56385	ATP5ME	Mitochondrion. Mitochondrion inner membrane.
Mitochondrial carrier homolog 2	Q9Y6C9	MTCH2	Mitochondrion inner membrane {ECO:0000305}; Multi-pass membrane protein {ECO:0000305}.
Hemoglobin subunit alpha	P69905	HBA1; HBA2	0
Tubulin beta chain	P07437	TUBB	Cytoplasm, cytoskeleton {ECO:0000269|PubMed:26637975}.
Heat shock protein beta-1	P04792	HSPB1	Cytoplasm {ECO:0000269|PubMed:10777697, ECO:0000269|PubMed:28144995}. Nucleus {ECO:0000269|PubMed:19464326}. Cytoplasm, cytoskeleton, spindle {ECO:0000269|PubMed:10777697}. Note=Cytoplasmic in interphase cells. Colocalizes with mitotic spindles in mitotic cells. Translocates to the nucleus during heat shock and resides in sub-nuclear structures known as SC35 speckles or nuclear splicing speckles. {ECO:0000269|PubMed:19464326}.

Supplemental Table 1 Page 5. All proteins quantitated in study

Protein Name	Uniprot	Gene	Subcellular localization
Immunoglobulin kappa light chain	P0DOX7	0	Secreted {ECO:0000303|PubMed:20176268, ECO:0000303|PubMed:22158414}. Cell membrane {ECO:0000303|PubMed:20176268, ECO:0000303|PubMed:22158414}.
Immunoglobulin gamma-1 heavy chain	P0DOX5	0	Secreted {ECO:0000303|PubMed:20176268, ECO:0000303|PubMed:22158414}. Cell membrane {ECO:0000303|PubMed:20176268, ECO:0000303|PubMed:22158414}.
Trimeric intracellular cation channel type B	Q9NVV0	TMEM38B	Endoplasmic reticulum membrane {ECO:0000250|UniProtKB:Q9DAV9}; Multi-pass membrane protein {ECO:0000250|UniProtKB:Q9DAV9}.
Probable cysteine--tRNA ligase, mitochondrial	Q9HA77	CARS2	Mitochondrion matrix {ECO:0000250}.
HLA class I histocompatibility antigen, B-38 alpha chain	Q95365	HLA-B	Membrane; Single-pass type I membrane protein.
NADH dehydrogenase [ubiquinone] 1 alpha subcomplex subunit 6	P56556	NDUFA6	Mitochondrion inner membrane {ECO:0000305|PubMed:12611891}; Peripheral membrane protein {ECO:0000305}; Matrix side {ECO:0000305}.
NADH dehydrogenase [ubiquinone] 1 beta subcomplex subunit 11, mitochondrial	Q9NX14	NDUFB11	Mitochondrion inner membrane {ECO:0000305|PubMed:12611891}; Single-pass membrane protein {ECO:0000305}.
Importin subunit alpha-3	O00629	KPNA4	Cytoplasm {ECO:0000250}. Nucleus {ECO:0000250}.
Aldehyde dehydrogenase X, mitochondrial	P30837	ALDH1B1	Mitochondrion matrix.
ATP synthase subunit delta, mitochondrial	P30049	ATP5F1D	Mitochondrion. Mitochondrion inner membrane.
ATP synthase subunit alpha, mitochondrial	P25705	ATP5F1A	Mitochondrion {ECO:0000269|PubMed:30146159}. Mitochondrion inner membrane {ECO:0000250|UniProtKB:P19483}; Peripheral membrane protein {ECO:0000250|UniProtKB:P19483}; Matrix side {ECO:0000250|UniProtKB:P19483}. Cell membrane {ECO:0000269|PubMed:10077593}; Peripheral membrane protein {ECO:0000269|PubMed:10077593}; Extracellular side {ECO:0000269|PubMed:10077593}. Note=Colocalizes with HRG on the cell surface of T-cells (PubMed:19285951). {ECO:0000269|PubMed:19285951}.

Supplemental Table 1 Page 6. All proteins quantitated in study

Protein Name	Uniprot	Gene	Subcellular localization
ATP-dependent RNA helicase DDX1	Q92499	DDX1	Nucleus. Cytoplasm. Cytoplasmic granule. Cytoplasm, cytosol {ECO:0000250|UniProtKB:Q91VR5}. Mitochondrion {ECO:0000250|UniProtKB:Q91VR5}. Note=Localized with MBNL1, TIAL1 and YBX1 in stress granules upon stress. Localized with CSTF2 in cleavage bodies. Forms large aggregates called DDX1 bodies. Relocalized into multiple foci (IR-induced foci or IRIF) after IR treatment, a process that depends on the presence of chromosomal DNA and/or RNA-DNA duplexes. Relocalized at sites of DNA double-strand breaks (DSBs) in an ATM-dependent manner after IR treatment. Colocalized with RELA in the nucleus upon TNF-alpha induction. Enters into the nucleus in case of active transcription while it accumulates in cytosol when transcription level is low (PubMed:24608264). Colocalizes in the cytosol with DDX21, DHX36 and TICAM1. Colocalizes in the mitochondria with TICAM1 and poly(I:C) RNA ligand. The multi-helicase-TICAM1 complex may translocate to the mitochondria upon poly(I:C) stimulation (By similarity). {ECO:0000250|UniProtKB:Q91VR5, ECO:0000269|PubMed:24608264}.; Cytoplasm {ECO:0000269|PubMed:20573827}. Note=(Microbial infection) Relocalized to the cytoplasm with a perinuclear staining pattern in avian infectious bronchitis virus (IBV)-infected cells (PubMed:20573827). Required for proper localization of HIV-1 Rev (PubMed:15567440). {ECO:0000269|PubMed:15567440, ECO:0000269|PubMed:20573827}.
Four and a half LIM domains protein 1	Q13642	FHL1	Isoform 1: Cytoplasm.; Isoform 3: Cytoplasm. Nucleus.; Isoform 2: Nucleus. Cytoplasm, cytosol. Note=Predominantly nuclear in myoblasts but is cytosolic in differentiated myotubes.
Heat shock 70 kDa protein 1A	P0DMV8	HSPA1A	Cytoplasm {ECO:0000269|PubMed:17289661}. Nucleus {ECO:0000269|PubMed:27137183}. Cytoplasm, cytoskeleton, microtubule organizing center, centrosome {ECO:0000269|PubMed:27137183}. Note=Localized in cytoplasmic mRNP granules containing untranslated mRNAs.
HLA class I histocompatibility antigen, A-24 alpha chain	P05534	HLA-A	Membrane; Single-pass type I membrane protein.
Tripeptidyl-peptidase 1	O14773	TPP1	Lysosome {ECO:0000269|PubMed:19941651}. Melanosome {ECO:0000269|PubMed:12643545}. Note=Identified by mass spectrometry in melanosome fractions from stage I to stage IV. {ECO:0000269|PubMed:12643545}.
Plectin	Q15149	PLEC	Cytoplasm, cytoskeleton {ECO:0000269|PubMed:12482924}. Cell junction, hemidesmosome {ECO:0000269|PubMed:12482924}.
Reticulon-2	O75298	RTN2	Endoplasmic reticulum membrane {ECO:0000305}; Multi-pass membrane protein {ECO:0000305}.
Elongation factor Tu, mitochondrial	P49411	TUFM	Mitochondrion {ECO:0000269|PubMed:22749352}.
2,4-dienoyl-CoA reductase, mitochondrial	Q16698	DECR1	Mitochondrion.

Supplemental Table 1 Page 7. All proteins quantitated in study

Protein Name	Uniprot	Gene	Subcellular localization
HLA class II histocompatibility antigen, DR alpha chain	P01903	HLA-DRA	Cell membrane; Single-pass type I membrane protein. Endoplasmic reticulum membrane; Single-pass type I membrane protein. Golgi apparatus, trans-Golgi network membrane; Single-pass type I membrane protein. Endosome membrane; Single-pass type I membrane protein. Lysosome membrane; Single-pass type I membrane protein. Late endosome membrane; Single-pass type I membrane protein. Note=The MHC class II complex transits through a number of intracellular compartments in the endocytic pathway until it reaches the cell membrane for antigen presentation.
Ubiquinone biosynthesis O-methyltransferase, mitochondrial	Q9NZJ6	COQ3	Mitochondrion inner membrane {ECO:0000255|HAMAP-Rule:MF_03190}; Peripheral membrane protein {ECO:0000255|HAMAP-Rule:MF_03190}; Matrix side {ECO:0000255|HAMAP-Rule:MF_03190}.
Creatine kinase M-type	P06732	CKM	Cytoplasm.
Sideroflexin-1	Q9H9B4	SFXN1	Mitochondrion inner membrane {ECO:0000269|PubMed:30442778}; Multi-pass membrane protein {ECO:0000255}.
2-oxoisovalerate dehydrogenase subunit alpha, mitochondrial	P12694	BCKDHA	Mitochondrion matrix.
Tubulin beta-4B chain	P68371	TUBB4B	Cytoplasm, cytoskeleton.
HLA class I histocompatibility antigen, B-41 alpha chain	P30479	HLA-B	Membrane; Single-pass type I membrane protein.
Immunoglobulin lambda constant 2	P0DOY2	IGLC2	Secreted {ECO:0000303|PubMed:20176268, ECO:0000303|PubMed:22158414}. Cell membrane {ECO:0000303|PubMed:20176268, ECO:0000303|PubMed:22158414}.
Glutathione hydrolase 5 proenzyme	P36269	GGT5	Membrane {ECO:0000250}; Single-pass type II membrane protein {ECO:0000250}.
Alpha-actinin-4	O43707	ACTN4	Nucleus {ECO:0000269|PubMed:22351778, ECO:0000269|PubMed:9508771}. Cytoplasm {ECO:0000269|PubMed:22351778, ECO:0000269|PubMed:9508771}. Cell junction {ECO:0000250|UniProtKB:P57780}. Cytoplasm, cytoskeleton, stress fiber {ECO:0000269|PubMed:9508771}. Note=Localized in cytoplasmic mRNP granules containing untranslated mRNAs. Nuclear translocation can be induced by the PI3 kinase inhibitor wortmannin or by cytochalasin D. Exclusively localized in the nucleus in a limited number of cell lines (breast cancer cell line MCF-7, oral floor cancer IMC-2, and bladder cancer KU-7). {ECO:0000269|PubMed:17289661, ECO:0000269|PubMed:9508771}.
Glutathione S-transferase kappa 1	Q9Y2Q3	GSTK1	Peroxisome {ECO:0000269|PubMed:14742434}.
Myosin-7	P12883	MYH7	Cytoplasm, myofibril {ECO:0000250|UniProtKB:P02564}. Cytoplasm, myofibril, sarcomere {ECO:0000250|UniProtKB:P02564}. Note=Thick filaments of the myofibrils. {ECO:0000250|UniProtKB:P02564}.
PDZ and LIM domain protein 3	Q53GG5	PDLIM3	Cytoplasm, myofibril, sarcomere, Z line {ECO:0000269|PubMed:10063829}. Note=Localizes to myofiber Z-lines.

Supplemental Table 1 Page 8. All proteins quantitated in study

Protein Name	Uniprot	Gene	Subcellular localization
Dihydrolipoyllysine-residue succinyltransferase component of 2-oxoglutarate dehydrogenase complex, mitochondrial	P36957	DLST	Mitochondrion matrix {ECO:0000305|PubMed:29211711}. Nucleus {ECO:0000269|PubMed:29211711}. Note=Mainly localizes in the mitochondrion. A small fraction localizes to the nucleus, where the 2-oxoglutarate dehydrogenase complex is required for histone succinylation. {ECO:0000269|PubMed:29211711}.
Troponin T, slow skeletal muscle	P13805	TNNT1	0
Superoxide dismutase [Mn], mitochondrial	P04179	SOD2	Mitochondrion matrix.
Mast cell carboxypeptidase A	P15088	CPA3	Cytoplasmic vesicle, secretory vesicle. Note=Secretory granules.
L-lactate dehydrogenase B chain	P07195	LDHB	Cytoplasm.
Translocator protein	P30536	TSPO	Mitochondrion membrane {ECO:0000269|PubMed:1847678}; Multi-pass membrane protein {ECO:0000269|PubMed:1847678}.
Mitochondrial 2-oxoglutarate/malate carrier protein	Q02978	SLC25A11	Mitochondrion inner membrane {ECO:0000250|UniProtKB:P97700}; Multi-pass membrane protein {ECO:0000250|UniProtKB:P97700}.
Pyruvate kinase PKM	P14618	PKM	Cytoplasm {ECO:0000269|PubMed:25263439}. Nucleus. Note=Translocates to the nucleus in response to different apoptotic stimuli. Nuclear translocation is sufficient to induce cell death that is caspase independent, isoform-specific and independent of its enzymatic activity.
14-3-3 protein gamma	P61981	YWHAG	Cytoplasm {ECO:0000250}.
Transmembrane protein 109	Q9BVC6	TMEM109	Nucleus outer membrane {ECO:0000250|UniProtKB:O77751}; Multi-pass membrane protein {ECO:0000250|UniProtKB:O77751}. Endoplasmic reticulum membrane {ECO:0000250|UniProtKB:O77751}; Multi-pass membrane protein {ECO:0000250|UniProtKB:O77751}. Sarcoplasmic reticulum membrane {ECO:0000250|UniProtKB:O77751}; Multi-pass membrane protein {ECO:0000250|UniProtKB:O77751}.
CDGSH iron-sulfur domain-containing protein 2	Q8N5K1	CISD2	Endoplasmic reticulum membrane; Single-pass membrane protein. Mitochondrion outer membrane; Single-pass membrane protein. Note=According to PubMed:20010695, it mainly localizes to the endoplasmic reticulum. However, experiments in mouse showed that it mainly localizes to the mitochondrion outer membrane.
Phosphate carrier protein, mitochondrial	Q00325	SLC25A3	Mitochondrion inner membrane; Multi-pass membrane protein.
NAD-dependent malic enzyme, mitochondrial	P23368	ME2	Mitochondrion matrix.
HIG1 domain family member 1A, mitochondrial	Q9Y241	HIGD1A	Mitochondrion membrane {ECO:0000255|PROSITE-ProRule:PRU00836, ECO:0000269|PubMed:22342701}; Multi-pass membrane protein {ECO:0000255|PROSITE-ProRule:PRU00836, ECO:0000269|PubMed:22342701}. Mitochondrion inner membrane {ECO:0000305|PubMed:22342701}.
NADH dehydrogenase [ubiquinone] 1 alpha subcomplex subunit 7	O95182	NDUFA7	Mitochondrion inner membrane {ECO:0000305|PubMed:12611891}; Peripheral membrane protein {ECO:0000305}; Matrix side {ECO:0000305}.

Supplemental Table 1 Page 9. All proteins quantitated in study

Protein Name	Uniprot	Gene	Subcellular localization
Desmin	P17661	DES	Cytoplasm, myofibril, sarcomere, Z line {ECO:0000269|PubMed:24200904, ECO:0000269|PubMed:26724190}. Cytoplasm {ECO:0000269|PubMed:25394388}. Cell membrane, sarcolemma {ECO:0000269|PubMed:25394388}. Nucleus {ECO:0000250|UniProtKB:P31001}. Note=Localizes in the intercalated disks which occur at the Z line of cardiomyocytes (PubMed:24200904, PubMed:26724190). Localizes in the nucleus exclusively in differentiating cardiac progenitor cells and premature cardiomyocytes (By similarity). {ECO:0000250|UniProtKB:P31001, ECO:0000269|PubMed:24200904, ECO:0000269|PubMed:26724190}.
3-ketoacyl-CoA thiolase, peroxisomal	P09110	ACAA1	Peroxisome.
Myc box-dependent-interacting protein 1	O00499	BIN1	Isoform BIN1: Nucleus {ECO:0000269|PubMed:8782822}. Cytoplasm {ECO:0000269|PubMed:9182667}. Endosome {ECO:0000250|UniProtKB:O08539}. Cell membrane, sarcolemma, T-tubule {ECO:0000250|UniProtKB:O08839}.; Isoform IIA: Cytoplasm {ECO:0000269|PubMed:9182667}.
Ferrochelatase, mitochondrial	P22830	FECH	Mitochondrion inner membrane; Peripheral membrane protein; Matrix side.
Serine/threonine-protein phosphatase 2A 65 kDa regulatory subunit A alpha isoform	P30153	PPP2R1A	Cytoplasm {ECO:0000250|UniProtKB:Q32PI5}. Nucleus {ECO:0000269|PubMed:30611118}. Chromosome, centromere {ECO:0000269|PubMed:16580887}. Lateral cell membrane {ECO:0000269|PubMed:15525651}. Cell projection, dendrite {ECO:0000269|PubMed:15525651}. Note=Centromeric localization requires the presence of BUB1. {ECO:0000269|PubMed:16580887}.
Chymase	P23946	CMA1	Secreted. Cytoplasmic granule. Note=Mast cell granules.
Perilipin-4	Q96Q06	PLIN4	Cell membrane {ECO:0000250|UniProtKB:O88492}. Cytoplasm {ECO:0000250|UniProtKB:O88492}. Lipid droplet {ECO:0000250|UniProtKB:O88492}. Note=Nascent lipid droplet surface-associated; association with lipid droplets is triacylglycerol synthesis-dependent. {ECO:0000250|UniProtKB:O88492}.
Alpha-enolase	P06733	ENO1	Cytoplasm {ECO:0000269|PubMed:10802057}. Cell membrane {ECO:0000269|PubMed:10802057}. Cytoplasm, myofibril, sarcomere, M line {ECO:0000269|PubMed:10802057}. Note=Can translocate to the plasma membrane in either the homodimeric (alpha/alpha) or heterodimeric (alpha/gamma) form. ENO1 is localized to the M line.; Isoform MBP-1: Nucleus.

Supplemental Table 1 Page 10. All proteins quantitated in study

Protein Name	Uniprot	Gene	Subcellular localization
cAMP-dependent protein kinase catalytic subunit alpha	P17612	PRKACA	Cytoplasm. Cell membrane. Nucleus {ECO:0000250}. Mitochondrion {ECO:0000250}. Membrane {ECO:0000305}; Lipid-anchor {ECO:0000305}. Note=Translocates into the nucleus (monomeric catalytic subunit). The inactive holoenzyme is found in the cytoplasm. Distributed throughout the cytoplasm in meiotically incompetent oocytes. Associated to mitochondrion as meiotic competence is acquired. Aggregates around the germinal vesicles (GV) at the immature GV stage oocytes (By similarity). Colocalizes with HSF1 in nuclear stress bodies (nSBs) upon heat shock (PubMed:21085490). {ECO:0000250, ECO:0000269|PubMed:21085490}.; Isoform 2: Cell projection, cilium, flagellum {ECO:0000269|PubMed:10906071}. Cytoplasmic vesicle, secretory vesicle, acrosome {ECO:0000250|UniProtKB:P05132}. Note=Expressed in the midpiece region of the sperm flagellum (PubMed:10906071). Colocalizes with MROH2B and TCP11 on the acrosome and tail regions in round spermatids and spermatozoa regardless of the capacitation status of the sperm (By similarity). {ECO:0000250|UniProtKB:P05132, ECO:0000269|PubMed:10906071}.
NADH dehydrogenase [ubiquinone] flavoprotein 2, mitochondrial	P19404	NDUFV2	Mitochondrion inner membrane.
Myosin light chain 6B	P14649	MYL6B	0
Carbonic anhydrase 3	P07451	CA3	Cytoplasm.

Supplemental Table 1 Page 11. All proteins quantitated in study

Protein Name	Uniprot	Gene	Subcellular localization
Annexin A1	P04083	ANXA1	Nucleus {ECO:0000269|PubMed:10772777, ECO:0000269|PubMed:19625660}. Cytoplasm {ECO:0000269|PubMed:10772777, ECO:0000269|PubMed:17008549, ECO:0000269|PubMed:19625660}. Cell projection, cilium {ECO:0000250|UniProtKB:P46193}. Cell membrane {ECO:0000269|PubMed:10772777}. Membrane {ECO:0000269|PubMed:17008549, ECO:0000269|PubMed:2532504, ECO:0000269|PubMed:8557678}; Peripheral membrane protein {ECO:0000269|PubMed:2532504, ECO:0000269|PubMed:8557678}. Endosome membrane {ECO:0000250|UniProtKB:P07150}; Peripheral membrane protein {ECO:0000250|UniProtKB:P07150}. Basolateral cell membrane {ECO:0000250|UniProtKB:P51662}. Apical cell membrane {ECO:0000250|UniProtKB:P10107}. Lateral cell membrane {ECO:0000250|UniProtKB:P10107}. Secreted {ECO:0000269|PubMed:17008549, ECO:0000269|PubMed:19625660, ECO:0000269|PubMed:25664854}. Secreted, extracellular space {ECO:0000269|PubMed:25664854}. Cell membrane {ECO:0000269|PubMed:10772777, ECO:0000269|PubMed:19625660, ECO:0000269|PubMed:25664854}; Peripheral membrane protein {ECO:0000269|PubMed:10772777, ECO:0000269|PubMed:19625660, ECO:0000269|PubMed:25664854}; Extracellular side {ECO:0000269|PubMed:10772777, ECO:0000269|PubMed:19625660, ECO:0000269|PubMed:25664854}. Secreted, exosome {ECO:0000269|PubMed:25664854}. Cytoplasmic vesicle, secretory vesicle lumen {ECO:0000269|PubMed:10772777}. Cell projection, phagocytic cup {ECO:0000250|UniProtKB:P10107}. Early endosome {ECO:0000250|UniProtKB:P19619}. Cytoplasmic vesicle membrane {ECO:0000250|UniProtKB:P19619}; Peripheral membrane protein {ECO:0000250|UniProtKB:P19619}. Note=Secreted, at least in part via exosomes and other secretory vesicles. Detected in exosomes and other extracellular vesicles (PubMed:25664854). Detected in gelatinase granules in resting neutrophils (PubMed:10772777). Secretion is increased in response to wounding and inflammation (PubMed:25664854). Secretion is increased upon T-cell activation (PubMed:17008549). Neutrophil adhesion to endothelial cells stimulates secretion via gelatinase granules, but foreign particle phagocytosis has no effect (PubMed:10772777). Colocalizes with actin fibers at phagocytic cups (By similarity). Displays calcium-dependent binding to phospholipid membranes (PubMed:2532504, PubMed:8557678). {ECO:0000250|UniProtKB:P10107, ECO:0000269|PubMed:10772777, ECO:0000269|PubMed:17008549, ECO:0000269|PubMed:2532504, ECO:0000269|PubMed:25664854, ECO:0000269|PubMed:8557678}.
Mitochondrial pyruvate carrier 1	Q9Y5U8	MPC1	Mitochondrion inner membrane {ECO:0000269|PubMed:22628558}; Multi-pass membrane protein {ECO:0000269|PubMed:22628558}.
Metaxin-1	Q13505	MTX1	Membrane {ECO:0000305}; Single-pass type I membrane protein {ECO:0000305}. Mitochondrion outer membrane {ECO:0000250}.
HLA class I histocompatibility antigen, A-3 alpha chain	P04439	HLA-A	Cell membrane {ECO:0000250|UniProtKB:P01892}; Single-pass type I membrane protein {ECO:0000255}. Endoplasmic reticulum membrane {ECO:0000250|UniProtKB:P01892}; Single-pass type I membrane protein {ECO:0000255}.

Supplemental Table 1 Page 12. All proteins quantitated in study

Protein Name	Uniprot	Gene	Subcellular localization
Calcium-binding mitochondrial carrier protein SCaMC-1	Q6NUK1	SLC25A24	Mitochondrion inner membrane {ECO:0000269|PubMed:15054102, ECO:0000269|PubMed:15123600, ECO:0000269|PubMed:22015608}; Multi-pass membrane protein {ECO:0000269|PubMed:15054102, ECO:0000269|PubMed:15123600, ECO:0000269|PubMed:22015608}.
10 kDa heat shock protein, mitochondrial	P61604	HSPE1	Mitochondrion matrix.
Myotilin	Q9UBF9	MYOT	Cell membrane, sarcolemma {ECO:0000269|PubMed:10369880}. Cytoplasm, cytoskeleton {ECO:0000269|PubMed:10369880}. Cytoplasm, myofibril, sarcomere, Z line {ECO:0000269|PubMed:16076904}. Note=Sarcomeric, also localized to the sarcolemma (PubMed:10369880). Colocalizes with MYOZ1 at the Z-lines in skeletal muscle (PubMed:16076904). {ECO:0000269|PubMed:10369880, ECO:0000269|PubMed:16076904}.
Guanine nucleotide-binding protein G(i) subunit alpha-2	P04899	GNAI2	Cytoplasm {ECO:0000269|PubMed:17635935}. Cytoplasm, cytoskeleton, microtubule organizing center, centrosome {ECO:0000269|PubMed:17635935}. Cell membrane {ECO:0000269|PubMed:17635935}. Membrane {ECO:0000305}; Lipid-anchor {ECO:0000305}. Note=Localizes in the centrosomes of interphase and mitotic cells. Detected at the cleavage furrow and/or the midbody.
Junctional sarcoplasmic reticulum protein 1	Q96MG2	JSRP1	Sarcoplasmic reticulum membrane {ECO:0000250}. Endoplasmic reticulum membrane {ECO:0000250}. Note=Colocalizes with ryanodine receptors at the sarcoplasmic reticulum triad membranes. {ECO:0000250}.
NADH dehydrogenase [ubiquinone] iron-sulfur protein 5	O43920	NDUFS5	Mitochondrion inner membrane {ECO:0000305|PubMed:12611891}; Peripheral membrane protein {ECO:0000305}. Mitochondrion intermembrane space {ECO:0000305}.
Collagen alpha-2(I) chain	P08123	COL1A2	Secreted, extracellular space, extracellular matrix {ECO:0000255|PROSITE-ProRule:PRU00793}.
Prohibitin	P35232	PHB	Mitochondrion inner membrane {ECO:0000269|PubMed:20959514}. Nucleus {ECO:0000269|PubMed:20959514}.
Calpain small subunit 1	P04632	CAPNS1	Cytoplasm {ECO:0000250}. Cell membrane {ECO:0000250}. Note=Translocates to the plasma membrane upon calcium binding. {ECO:0000250}.
Sarcalumenin	Q86TD4	SRL	Sarcoplasmic reticulum lumen {ECO:0000250}. Note=Associated through calcium with the membrane. {ECO:0000250}.

Supplemental Table 1 Page 13. All proteins quantitated in study

Protein Name	Uniprot	Gene	Subcellular localization
Unconventional myosin-Ic	O00159	MYO1C	Isoform 1: Cytoplasm {ECO:0000269|PubMed:22736583}. Nucleus {ECO:0000269|PubMed:22736583}. Note=Colocalizes with RNA polymerase II. Absent from nucleoli and does not colocalize with RNA polymerase I. Translocates to nuclear speckles upon exposure to inhibitors of RNA polymerase II transcription.; Isoform 2: Cytoplasm. Cell membrane {ECO:0000250}; Peripheral membrane protein {ECO:0000250}; Cytoplasmic side {ECO:0000250}. Cell projection, stereocilium membrane {ECO:0000250}. Cell projection, ruffle {ECO:0000250}. Cytoplasmic vesicle {ECO:0000250}. Note=Colocalizes with CABP1 and CIB1 at cell margin, membrane ruffles and punctate regions on the cell membrane. Colocalizes in adipocytes with GLUT4 at actin-based membranes. Colocalizes with GLUT4 at insulin-induced ruffles at the cell membrane. Localizes transiently at cell membrane to region known to be enriched in PIP2. Activation of phospholipase C results in its redistribution to the cytoplasm (By similarity). {ECO:0000250}.; Isoform 3: Nucleus, nucleoplasm. Nucleus, nucleolus. Nucleus, nuclear pore complex. Note=Colocalizes with RNA polymerase II in the nucleus. Colocalizes with RNA polymerase I in nucleoli (By similarity). In the nucleolus, is localized predominantly in dense fibrillar component (DFC) and in granular component (GC). Accumulates strongly in DFC and GC during activation of transcription. Colocalizes with transcription sites. Colocalizes in the granular cortex at the periphery of the nucleolus with RPS6. Colocalizes in nucleoplasm with RPS6 and actin that are in contact with RNP particles. Colocalizes with RPS6 at the nuclear pore level. {ECO:0000250}.
Ezrin	P15311	EZR	Apical cell membrane {ECO:0000269|PubMed:18046454}; Peripheral membrane protein {ECO:0000269|PubMed:18046454}; Cytoplasmic side {ECO:0000269|PubMed:18046454}. Cell projection {ECO:0000269|PubMed:18046454}. Cell projection, microvillus membrane {ECO:0000269|PubMed:18046454}; Peripheral membrane protein {ECO:0000269|PubMed:18046454}; Cytoplasmic side {ECO:0000269|PubMed:18046454}. Cell projection, ruffle membrane {ECO:0000269|PubMed:18046454}; Peripheral membrane protein {ECO:0000269|PubMed:18046454}; Cytoplasmic side {ECO:0000269|PubMed:18046454}. Cytoplasm, cell cortex {ECO:0000269|PubMed:18046454}. Cytoplasm, cytoskeleton {ECO:0000269|PubMed:18046454}. Cell projection, microvillus {ECO:0000250|UniProtKB:P26040}. Note=Localization to the apical membrane of parietal cells depends on the interaction with MPP5. Localizes to cell extensions and peripheral processes of astrocytes (By similarity). Microvillar peripheral membrane protein (cytoplasmic side). {ECO:0000250|UniProtKB:P31977}.
Peptidyl-prolyl cis-trans isomerase FKBP3	Q00688	FKBP3	Nucleus.
Sulfide:quinone oxidoreductase, mitochondrial	Q9Y6N5	SQOR	Mitochondrion {ECO:0000305}.

Supplemental Table 1 Page 14. All proteins quantitated in study

Protein Name	Uniprot	Gene	Subcellular localization
Redox-regulatory protein FAM213A	Q9BRX8	PRXL2A	Cytoplasm {ECO:0000269|PubMed:19951071}. Secreted {ECO:0000269|PubMed:26438880}. Note=Secreted from mature adipocytes but not from preadipocytes. {ECO:0000269|PubMed:26438880}.
Fibrinogen gamma chain	P02679	FGG	Secreted {ECO:0000269|PubMed:10074346, ECO:0000269|PubMed:19296670, ECO:0000269|PubMed:9628725}.
Erythrocyte band 7 integral membrane protein	P27105	STOM	Cell membrane {ECO:0000269|PubMed:9243190}; Peripheral membrane protein {ECO:0000269|PubMed:9243190}; Cytoplasmic side {ECO:0000269|PubMed:9243190}. Cytoplasm, cytoskeleton {ECO:0000269|PubMed:9243190}. Cell membrane {ECO:0000269|PubMed:12130500}; Lipid-anchor {ECO:0000269|PubMed:12130500}; Cytoplasmic side {ECO:0000269|PubMed:12130500}. Membrane raft {ECO:0000269|PubMed:12130500, ECO:0000269|PubMed:23219802}. Melanosome {ECO:0000269|PubMed:12643545, ECO:0000269|PubMed:17081065}. Cytoplasmic vesicle {ECO:0000250|UniProtKB:P54116}. Note=Localizes to juxtanuclear structure probably derived from the Golgi apparatus (PubMed:9243190). Colocalizes with cortical actin microfilaments at small plasma membrane protrusions (PubMed:9243190). Associates with alpha-granular lipid rafts (PubMed:12130500). Translocates from the alpha-granular lipid rafts to the cell membrane on thrombin activation and selectively enriched in released microvesicles (PubMed:12130500). Identified by mass spectrometry in melanosome fractions from stage I to stage IV (PubMed:12643545). {ECO:0000269|PubMed:12130500, ECO:0000269|PubMed:12643545, ECO:0000269|PubMed:9243190}.
NADH dehydrogenase [ubiquinone] 1 alpha subcomplex subunit 10, mitochondrial	O95299	NDUFA10	Mitochondrion matrix {ECO:0000305|PubMed:12611891}.
Blood group Rh(D) polypeptide	Q02161	RHD	Cell membrane {ECO:0000269|PubMed:1544931, ECO:0000269|PubMed:3142870}; Multi-pass membrane protein {ECO:0000255}.
Tropomyosin alpha-3 chain	P06753	TPM3	Cytoplasm, cytoskeleton {ECO:0000305}.
Peroxiredoxin-4	Q13162	PRDX4	Cytoplasm {ECO:0000269|PubMed:18052930, ECO:0000269|PubMed:9388242}. Endoplasmic reticulum {ECO:0000269|PubMed:18052930}. Note=Cotranslationally translocated to and retained within the endoplasmic reticulum. A small fraction of the protein is cytoplasmic. {ECO:0000269|PubMed:18052930}.
Cytochrome b-c1 complex subunit 8	O14949	UQCRQ	Mitochondrion inner membrane.
Long-chain-fatty-acid--CoA ligase 1	P33121	ACSL1	Mitochondrion outer membrane {ECO:0000250}; Single-pass type III membrane protein {ECO:0000250}. Peroxisome membrane {ECO:0000250}; Single-pass type III membrane protein {ECO:0000250}. Microsome membrane {ECO:0000250}; Single-pass type III membrane protein {ECO:0000250}. Endoplasmic reticulum membrane {ECO:0000250}; Single-pass type III membrane protein {ECO:0000250}.

Supplemental Table 1 Page 15. All proteins quantitated in study

Protein Name	Uniprot	Gene	Subcellular localization
NADH dehydrogenase [ubiquinone] iron-sulfur protein 3, mitochondrial	O75489	NDUFS3	Mitochondrion inner membrane.
NADH dehydrogenase [ubiquinone] 1 alpha subcomplex subunit 8	P51970	NDUFA8	Mitochondrion inner membrane {ECO:0000269|PubMed:21310150}; Peripheral membrane protein {ECO:0000269|PubMed:21310150}. Mitochondrion intermembrane space {ECO:0000269|PubMed:21310150}. Mitochondrion {ECO:0000269|PubMed:23676665}.
Heat shock-related 70 kDa protein 2	P54652	HSPA2	Cytoplasm, cytoskeleton, spindle {ECO:0000250|UniProtKB:P17156}. Note=Colocalizes with SHCBP1L at spindle during the meiosis process. {ECO:0000250|UniProtKB:P17156}.
Sideroflexin-3	Q9BWM7	SFXN3	Mitochondrion membrane {ECO:0000269|PubMed:30442778, ECO:0000269|Ref.4}; Multi-pass membrane protein {ECO:0000255}.
Malate dehydrogenase, cytoplasmic	P40925	MDH1	Cytoplasm.
Myosin-9	P35579	MYH9	Cytoplasm, cytoskeleton {ECO:0000250|UniProtKB:Q8VDD5}. Cytoplasm, cell cortex {ECO:0000250|UniProtKB:Q8VDD5}. Note=Colocalizes with actin filaments at lamellipodia margins and at the leading edge of migrating cells (PubMed:20052411). In retinal pigment epithelial cells, predominantly localized to stress fiber-like structures with some localization to cytoplasmic puncta (PubMed:27331610). {ECO:0000269|PubMed:20052411, ECO:0000269|PubMed:27331610}.
Ecto-ADP-ribosyltransferase 3	Q13508	ART3	Cell membrane; Lipid-anchor, GPI-anchor.
Collagen alpha-1(VI) chain	P12109	COL6A1	Secreted, extracellular space, extracellular matrix {ECO:0000250}.
Alpha-actinin-2	P35609	ACTN2	Cytoplasm, myofibril, sarcomere, Z line {ECO:0000269|PubMed:11171996, ECO:0000269|PubMed:19932097}. Note=Colocalizes with MYOZ1 and FLNC at the Z-lines of skeletal muscle.
Immunoglobulin heavy constant alpha 1	P01876	IGHA1	Secreted {ECO:0000303|PubMed:20176268, ECO:0000303|PubMed:22158414}. Cell membrane {ECO:0000303|PubMed:20176268, ECO:0000303|PubMed:22158414}.
Mitochondrial import inner membrane translocase subunit TIM44	O43615	TIMM44	Mitochondrion inner membrane {ECO:0000269|PubMed:10339406}; Peripheral membrane protein {ECO:0000269|PubMed:10339406}; Matrix side {ECO:0000269|PubMed:10339406}. Mitochondrion matrix {ECO:0000269|PubMed:10339406}.
Polyribonucleotide nucleotidyltransferase 1, mitochondrial	Q8TCS8	PNPT1	Cytoplasm. Mitochondrion. Mitochondrion intermembrane space; Peripheral membrane protein.
Cytochrome c oxidase subunit 5A, mitochondrial	P20674	COX5A	Mitochondrion inner membrane.
Collagen alpha-1(IV) chain	P02462	COL4A1	Secreted, extracellular space, extracellular matrix, basement membrane {ECO:0000250|UniProtKB:P02463}.
Vesicle-associated membrane protein-associated protein B/C	O95292	VAPB	Endoplasmic reticulum membrane {ECO:0000250}; Single-pass type IV membrane protein {ECO:0000250}. Note=Present in mitochondria-associated membranes that are endoplasmic reticulum membrane regions closely apposed to the outer mitochondrial membrane. {ECO:0000269|PubMed:22131369}.

Supplemental Table 1 Page 16. All proteins quantitated in study

Protein Name	Uniprot	Gene	Subcellular localization
Basic leucine zipper and W2 domain-containing protein 2	Q9Y6E2	BZW2	0
3-oxoacyl-[acyl-carrier-protein] synthase, mitochondrial	Q9NWU1	OXSM	Mitochondrion {ECO:0000269|PubMed:15668256}.
Alpha-1B-glycoprotein	P04217	A1BG	Secreted.
Ras-related protein Ral-A	P11233	RALA	Cell membrane {ECO:0000269|PubMed:17875936, ECO:0000269|PubMed:18756269, ECO:0000269|PubMed:19306925}; Lipid-anchor {ECO:0000269|PubMed:17875936}; Cytoplasmic side. Cleavage furrow {ECO:0000269|PubMed:18756269}. Midbody, Midbody ring {ECO:0000269|PubMed:16213214}. Note=Predominantly at the cell surface in the absence of LPA. In the presence of LPA, colocalizes with LPAR1 and LPAR2 in endocytic vesicles (PubMed:19306925). May colocalize with CNTRL/centriolin at the midbody ring (PubMed:16213214). However, localization at the midbody at late cytokinesis was not confirmed (PubMed:18756269). {ECO:0000269|PubMed:16213214, ECO:0000269|PubMed:18756269, ECO:0000269|PubMed:19306925}.
Serine hydroxymethyltransferase, mitochondrial	P34897	SHMT2	Mitochondrion {ECO:0000269|PubMed:21876188, ECO:0000269|PubMed:24075985}. Mitochondrion matrix, mitochondrion nucleoid {ECO:0000269|PubMed:18063578}. Mitochondrion inner membrane {ECO:0000269|PubMed:21876188}. Cytoplasm {ECO:0000269|PubMed:24075985}. Nucleus {ECO:0000269|PubMed:24075985}. Note=Mainly localizes in the mitochondrion. Also found in the cytoplasm and nucleus as part of the BRISC complex (PubMed:24075985). {ECO:0000269|PubMed:24075985}.
Serotransferrin	P02787	TF	Secreted.
Titin	Q8WZ42	TTN	Cytoplasm {ECO:0000305|PubMed:16410549}. Nucleus {ECO:0000269|PubMed:16410549}.
Pyruvate dehydrogenase protein X component, mitochondrial	O00330	PDHX	Mitochondrion matrix.
ATP-dependent 6-phosphofructokinase, muscle type	P08237	PFKM	Cytoplasm {ECO:0000255|HAMAP-Rule:MF_03184}.
ADP/ATP translocase 2	P05141	SLC25A5	Mitochondrion inner membrane; Multi-pass membrane protein.
Annexin A2	P07355	ANXA2	Secreted, extracellular space, extracellular matrix, basement membrane {ECO:0000269|PubMed:17081065}. Melanosome {ECO:0000269|PubMed:17081065}. Note=In the lamina beneath the plasma membrane. Identified by mass spectrometry in melanosome fractions from stage I to stage IV. Translocated from the cytoplasm to the cell surface through a Golgi-independent mechanism.
Mitochondrial fission 1 protein	Q9Y3D6	FIS1	Mitochondrion outer membrane; Single-pass membrane protein. Peroxisome membrane; Single-pass membrane protein.
Biglycan	P21810	BGN	Secreted, extracellular space, extracellular matrix {ECO:0000250}.
28S ribosomal protein S35, mitochondrial	P82673	MRPS35	Mitochondrion {ECO:0000269|PubMed:25838379}.

Supplemental Table 1 Page 17. All proteins quantitated in study

Protein Name	Uniprot	Gene	Subcellular localization
Hemoglobin subunit beta	P68871	HBB	0
Myozenin-2	Q9NPC6	MYOZ2	Cytoplasm, myofibril, sarcomere, Z line {ECO:0000250}. Note=Colocalizes with ACTN1 and PPP3CA at the Z-line of heart and skeletal muscle. {ECO:0000250}.
Acyl carrier protein, mitochondrial	O14561	NDUFAB1	Mitochondrion {ECO:0000269|PubMed:12611891, ECO:0000269|PubMed:28892042}.
Eukaryotic initiation factor 4A-I	P60842	EIF4A1	0
Myosin-binding protein C, slow-type	Q00872	MYBPC1	0
Galectin-1	P09382	LGALS1	Secreted, extracellular space, extracellular matrix {ECO:0000269|PubMed:14617626}.
NADH-cytochrome b5 reductase 1	Q9UHQ9	CYB5R1	Membrane {ECO:0000305}; Single-pass membrane protein {ECO:0000305}.
Sodium/potassium-transporting ATPase subunit alpha-1	P05023	ATP1A1	Cell membrane, sarcolemma {ECO:0000269|PubMed:7711835}; Multi-pass membrane protein {ECO:0000255}. Melanosome {ECO:0000269|PubMed:17081065}. Note=Identified by mass spectrometry in melanosome fractions from stage I to stage IV. {ECO:0000269|PubMed:17081065}.
Peptidyl-prolyl cis-trans isomerase F, mitochondrial	P30405	PPIF	Mitochondrion matrix {ECO:0000269|PubMed:10406942}.
Medium-chain specific acyl-CoA dehydrogenase, mitochondrial	P11310	ACADM	Mitochondrion matrix.
Calsequestrin-1	P31415	CASQ1	Endoplasmic reticulum {ECO:0000269|PubMed:27185316, ECO:0000269|PubMed:28895244}. Sarcoplasmic reticulum {ECO:0000269|PubMed:27196359}. Sarcoplasmic reticulum lumen {ECO:0000250|UniProtKB:P07221}. Sarcoplasmic reticulum membrane; Peripheral membrane protein; Lumenal side {ECO:0000250|UniProtKB:P07221}. Mitochondrion matrix {ECO:0000250|UniProtKB:O09165}. Note=This isoform of calsequestrin occurs in the sarcoplasmic reticulum's terminal cisternae luminal spaces of fast skeletal muscle cells. Preferentially forms linear and round aggregates in the endoplasmic reticulum (ER) of resting cells (PubMed:28895244). In a minority of cells, homogeneously detected in the ER lumen (PubMed:28895244). Colocalizes with STIM1 at endoplasmic reticulum in response to a depletion of intracellular calcium (PubMed:27185316). {ECO:0000250|UniProtKB:P07221, ECO:0000269|PubMed:27185316, ECO:0000269|PubMed:28895244}.
O-acetyl-ADP-ribose deacetylase MACROD1	Q9BQ69	MACROD1	Nucleus {ECO:0000269|PubMed:23474712}. Note=Recruited to DNA lesions, probably via mono-APD-ribosylated proteins. {ECO:0000269|PubMed:23474712}.
Tropomodulin-4	Q9NZQ9	TMOD4	Cytoplasm, cytoskeleton {ECO:0000269|PubMed:25250574}. Note=In myofibrils with sarcomeric structure, localizes to the pointed end of actin thin filaments (PubMed:25250574). {ECO:0000269|PubMed:25250574}.
FH1/FH2 domain-containing protein 1	Q9Y613	FHOD1	Cytoplasm. Cytoplasm, cytoskeleton. Cell projection, bleb. Note=Predominantly cytoplasmic.

Supplemental Table 1 Page 18. All proteins quantitated in study

Protein Name	Uniprot	Gene	Subcellular localization
Microsomal glutathione S-transferase 3	O14880	MGST3	Endoplasmic reticulum membrane {ECO:0000305}; Multi-pass membrane protein {ECO:0000305}. Microsome membrane {ECO:0000305}; Multi-pass membrane protein {ECO:0000305}. Microsome membrane {ECO:0000305}; Lipid-anchor {ECO:0000305}. Membrane {ECO:0000269|PubMed:9278457}.
Heat shock protein HSP 90-alpha	P07900	HSP90AA1	Nucleus {ECO:0000250|UniProtKB:P07901}. Cytoplasm {ECO:0000250|UniProtKB:P07901}. Melanosome {ECO:0000269|PubMed:17081065}. Cell membrane {ECO:0000269|PubMed:11276205}. Note=Identified by mass spectrometry in melanosome fractions from stage I to stage IV.
ATP synthase subunit f, mitochondrial	P56134	ATP5MF	Mitochondrion. Mitochondrion inner membrane {ECO:0000305}; Single-pass membrane protein {ECO:0000305}.
Protein FAM210A	Q96ND0	FAM210A	Membrane {ECO:0000255, ECO:0000305}; Single-pass membrane protein {ECO:0000255}. Mitochondrion {ECO:0000250|UniProtKB:Q8BGY7}. Cytoplasm {ECO:0000250|UniProtKB:Q8BGY7}.
Epoxide hydrolase 1	P07099	EPHX1	Microsome membrane {ECO:0000250|UniProtKB:P07687}; Single-pass type III membrane protein {ECO:0000250|UniProtKB:P07687}. Endoplasmic reticulum membrane {ECO:0000250|UniProtKB:P07687}; Single-pass type III membrane protein {ECO:0000250|UniProtKB:P07687}.
GTP-binding protein SAR1b	Q9Y6B6	SAR1B	Endoplasmic reticulum membrane {ECO:0000250}; Peripheral membrane protein {ECO:0000250}. Golgi apparatus, Golgi stack membrane {ECO:0000250}; Peripheral membrane protein {ECO:0000250}. Note=Associated with the endoplasmic reticulum and Golgi stacks, in particular in the juxta-nuclear Golgi region. {ECO:0000250}.
Actin, alpha cardiac muscle 1	P68032	ACTC1	Cytoplasm, cytoskeleton.
Collagen alpha-2(VI) chain	P12110	COL6A2	Secreted, extracellular space, extracellular matrix {ECO:0000269|PubMed:8305732}. Membrane {ECO:0000269|PubMed:8305732}; Peripheral membrane protein {ECO:0000269|PubMed:8305732}. Note=Recruited on membranes by CSPG4.
Myosin regulatory light chain 12A	P19105	MYL12A	0
Myosin regulatory light chain 2, ventricular/cardiac muscle isoform	P10916	MYL2	Cytoplasm, myofibril, sarcomere, A band {ECO:0000250|UniProtKB:P08733}.
Kelch-like protein 41	O60662	KLHL41	Cytoplasm {ECO:0000269|PubMed:19424503}. Cytoplasm, cytoskeleton {ECO:0000250|UniProtKB:A2AUC9}. Cell projection, pseudopodium {ECO:0000250|UniProtKB:Q9ER30}. Cell projection, ruffle {ECO:0000250|UniProtKB:Q9ER30}. Cytoplasm, myofibril, sarcomere, M line {ECO:0000250|UniProtKB:A2AUC9}. Sarcoplasmic reticulum membrane {ECO:0000269|PubMed:24268659}. Endoplasmic reticulum membrane {ECO:0000269|PubMed:24268659}. Note=Predominantly cytoplasmic but can colocalize with F-actin at the membrane ruffle-like structures at the tips of transformation-specific pseudopodia. {ECO:0000269|PubMed:19424503}.
Collagen alpha-1(III) chain	P02461	COL3A1	Secreted, extracellular space, extracellular matrix {ECO:0000255|PROSITE-ProRule:PRU00793}.

Supplemental Table 1 Page 19. All proteins quantitated in study

Protein Name	Uniprot	Gene	Subcellular localization
Cytochrome c oxidase subunit 7A-related protein, mitochondrial	O14548	COX7A2L	Mitochondrion inner membrane {ECO:0000250}.
5'-AMP-activated protein kinase subunit gamma-1	P54619	PRKAG1	0
Surfeit locus protein 4	O15260	SURF4	Endoplasmic reticulum membrane {ECO:0000269|PubMed:15308636, ECO:0000269|PubMed:18287528}; Multi-pass membrane protein {ECO:0000255}. Endoplasmic reticulum-Golgi intermediate compartment membrane {ECO:0000269|PubMed:18287528}; Multi-pass membrane protein {ECO:0000255}. Golgi apparatus membrane {ECO:0000269|PubMed:18287528}; Multi-pass membrane protein {ECO:0000255}. Note=Cycles between the endoplasmic reticulum and the Golgi. {ECO:0000269|PubMed:18287528}.
Mitochondrial-processing peptidase subunit beta	O75439	PMPCB	Mitochondrion matrix {ECO:0000250}.
Enoyl-CoA hydratase domain-containing protein 2, mitochondrial	Q86YB7	ECHDC2	Mitochondrion {ECO:0000305}.
Eukaryotic translation initiation factor 3 subunit F	O00303	EIF3F	Cytoplasm {ECO:0000255|HAMAP-Rule:MF_03005}.
Protein NipSnap homolog 2	O75323	NIPSNAP2	Cytoplasm {ECO:0000250|UniProtKB:O55126}. Mitochondrion outer membrane {ECO:0000269|PubMed:26387735}.
Adenylyl cyclase-associated protein 2	P40123	CAP2	Cell membrane {ECO:0000250}; Peripheral membrane protein {ECO:0000250}.
NADH dehydrogenase [ubiquinone] 1 beta subcomplex subunit 6	O95139	NDUFB6	Mitochondrion inner membrane {ECO:0000305|PubMed:12611891}; Single-pass membrane protein {ECO:0000255}; Matrix side {ECO:0000305}.
Collagen alpha-3(VI) chain	P12111	COL6A3	Secreted, extracellular space, extracellular matrix {ECO:0000250}.
Heterogeneous nuclear ribonucleoprotein K	P61978	HNRNPK	Cytoplasm {ECO:0000269|PubMed:1729596}. Nucleus, nucleoplasm {ECO:0000269|PubMed:16360036, ECO:0000269|PubMed:1729596, ECO:0000269|PubMed:18775702, ECO:0000269|PubMed:22721921}. Cell projection, podosome {ECO:0000269|PubMed:22721921}. Note=Recruited to p53/TP53-responsive promoters, in the presence of functional p53/TP53 (PubMed:16360036). In case of ASFV infection, there is a shift in the localization which becomes predominantly nuclear (PubMed:18775702).
Troponin C, skeletal muscle	P02585	TNNC2	0
Solute carrier family 2, facilitated glucose transporter member 1	P11166	SLC2A1	Cell membrane; Multi-pass membrane protein. Melanosome. Note=Localizes primarily at the cell surface. Identified by mass spectrometry in melanosome fractions from stage I to stage IV.
Fibrinogen beta chain	P02675	FGB	Secreted {ECO:0000269|PubMed:10074346, ECO:0000269|PubMed:19296670, ECO:0000269|PubMed:9628725}.

Supplemental Table 1 Page 20. All proteins quantitated in study

Protein Name	Uniprot	Gene	Subcellular localization
Ras-related protein Rab-21	Q9UL25	RAB21	Endoplasmic reticulum membrane {ECO:0000269|PubMed:10887961}; Lipid-anchor {ECO:0000305}. Golgi apparatus, trans-Golgi network {ECO:0000269|PubMed:19745841}. Golgi apparatus membrane {ECO:0000305}. Early endosome membrane {ECO:0000269|PubMed:16525121, ECO:0000269|PubMed:25648148}. Cytoplasmic vesicle membrane {ECO:0000269|PubMed:10887961}. Cleavage furrow {ECO:0000269|PubMed:18804435}. Note=Colocalizes with ANKRD27 and VAMP7 in neurites (By similarity). In nonpolarized epithelial Caco-2 cells, found in the endoplasmic reticulum; in polarized cells, observed in vesicles in the apical cytoplasm (PubMed:10887961). During mitosis, in mid-telophase, localized in the ingressing cleavage furrow (PubMed:18804435). In late telophase, detected at the opposite poles of the daughter cells, in vesicles at the base of lamellipodia formed by the separating daughter cells (PubMed:18804435). {ECO:0000250|UniProtKB:P35282, ECO:0000269|PubMed:10887961, ECO:0000269|PubMed:18804435}.
ADP/ATP translocase 1	P12235	SLC25A4	Mitochondrion inner membrane; Multi-pass membrane protein.
Prelamin-A/C	P02545	LMNA	Nucleus {ECO:0000269|PubMed:15372542}. Nucleus envelope {ECO:0000269|PubMed:29599122}. Nucleus lamina. Nucleus, nucleoplasm. Note=Farnesylation of prelamin-A/C facilitates nuclear envelope targeting and subsequent cleaveage by ZMPSTE24/FACE1 to remove the farnesyl group produces mature lamin-A/C, which can then be inserted into the nuclear lamina. EMD is required for proper localization of non-farnesylated prelamin-A/C.; Isoform C: Nucleus speckle {ECO:0000269|PubMed:16061563}.
FUN14 domain-containing protein 2	Q9BWH2	FUNDC2	0
ATP synthase subunit gamma, mitochondrial	P36542	ATP5F1C	Mitochondrion inner membrane {ECO:0000250|UniProtKB:P05631}; Peripheral membrane protein {ECO:0000250|UniProtKB:P05631}; Matrix side {ECO:0000250|UniProtKB:P05631}.

Supplemental Table 1 Page 21. All proteins quantitated in study

Protein Name	Uniprot	Gene	Subcellular localization
Moesin	P26038	MSN	Cell membrane {ECO:0000269|PubMed:11728332, ECO:0000269|PubMed:15039356, ECO:0000269|PubMed:18586956, ECO:0000269|PubMed:7884872, ECO:0000269|PubMed:9298994}; Peripheral membrane protein {ECO:0000250|UniProtKB:P26041}; Cytoplasmic side {ECO:0000250|UniProtKB:P26041}. Cytoplasm, cytoskeleton {ECO:0000250|UniProtKB:P26041}. Apical cell membrane {ECO:0000250|UniProtKB:P26041}; Peripheral membrane protein {ECO:0000250|UniProtKB:P26041}; Cytoplasmic side {ECO:0000250|UniProtKB:P26041}. Cell projection, microvillus membrane {ECO:0000250|UniProtKB:P26041}; Peripheral membrane protein {ECO:0000250|UniProtKB:P26041}; Cytoplasmic side {ECO:0000250|UniProtKB:P26041}. Cell projection, microvillus {ECO:0000250|UniProtKB:P26041}. Note=Phosphorylated form is enriched in microvilli-like structures at apical membrane. Increased cell membrane localization of both phosphorylated and non-phosphorylated forms seen after thrombin treatment (By similarity). Localizes at the uropods of T lymphoblasts. {ECO:0000250|UniProtKB:P26041, ECO:0000269|PubMed:18586956, ECO:0000269|PubMed:9298994}.
Starch-binding domain-containing protein 1	O95210	STBD1	Preautophagosomal structure membrane {ECO:0000269|PubMed:20810658, ECO:0000305|PubMed:21893048}; Single-pass type III membrane protein {ECO:0000305|PubMed:9794794}. Endoplasmic reticulum membrane {ECO:0000269|PubMed:24837458}; Single-pass type III membrane protein {ECO:0000305|PubMed:9794794}. Cell membrane, sarcolemma, T-tubule {ECO:0000269|PubMed:9794794}. Note=Also detected near the junctional sarcoplasmic reticulum (PubMed:9794794). Concentrates at perinuclear structures (PubMed:21893048). {ECO:0000269|PubMed:21893048, ECO:0000269|PubMed:9794794}.
MICOS complex subunit MIC60	Q16891	IMMT	Mitochondrion inner membrane {ECO:0000269|PubMed:25764979, ECO:0000269|PubMed:25997101}; Single-pass membrane protein {ECO:0000255}. Mitochondrion {ECO:0000269|PubMed:25781180}.
Coronin-6	Q6QEF8	CORO6	0
Myozenin-1	Q9NP98	MYOZ1	Nucleus. Cell projection, pseudopodium. Note=Localized to the nucleus and pseudopodia of undifferentiated cells and detected throughout the myotubes of differentiated cells. Colocalizes with ACTN2, FLNC and MYOT at the Z-lines of skeletal muscle.
Vimentin	P08670	VIM	Cytoplasm {ECO:0000269|PubMed:21465480, ECO:0000269|PubMed:29496907}. Cytoplasm, cytoskeleton {ECO:0000269|PubMed:29496907}. Nucleus matrix {ECO:0000250|UniProtKB:P31000}.
Beta-2-syntrophin	Q13425	SNTB2	Membrane. Cytoplasmic vesicle, secretory vesicle membrane; Peripheral membrane protein. Cell junction {ECO:0000250}. Cytoplasm, cytoskeleton. Note=Membrane-associated. In muscle, it is exclusively localized at the neuromuscular junction (By similarity). In insulinoma cell line, it is enriched in secretory granules. {ECO:0000250}.

Supplemental Table 1 Page 22. All proteins quantitated in study

Protein Name	Uniprot	Gene	Subcellular localization
Junctophilin-1	Q9HDC5	JPH1	Cell membrane {ECO:0000250}; Peripheral membrane protein {ECO:0000250}. Endoplasmic reticulum membrane {ECO:0000250}; Single-pass type IV membrane protein {ECO:0000250}. Sarcoplasmic reticulum membrane {ECO:0000250}; Single-pass type IV membrane protein {ECO:0000250}. Note=Localized predominantly on the plasma membrane. The transmembrane domain is anchored in endoplasmic/sarcoplasmic reticulum membrane, while the N-terminal part associates with the plasma membrane. In skeletal muscle cells, it is predominantly localized at the junction of the A and I bands (By similarity). {ECO:0000250}.
Cofilin-1	P23528	CFL1	Nucleus matrix {ECO:0000269|PubMed:15580268}. Cytoplasm, cytoskeleton {ECO:0000269|PubMed:15580268}. Cell projection, ruffle membrane {ECO:0000269|PubMed:15580268}; Peripheral membrane protein {ECO:0000269|PubMed:15580268}; Cytoplasmic side {ECO:0000269|PubMed:15580268}. Cell projection, lamellipodium membrane {ECO:0000269|PubMed:15580268}; Peripheral membrane protein {ECO:0000269|PubMed:15580268}; Cytoplasmic side {ECO:0000269|PubMed:15580268}. Cell projection, lamellipodium {ECO:0000250|UniProtKB:P18760}. Note=Colocalizes with the actin cytoskeleton in membrane ruffles and lamellipodia. Detected at the cleavage furrow and contractile ring during cytokinesis. Almost completely in nucleus in cells exposed to heat shock or 10% dimethyl sulfoxide.
Decorin	P07585	DCN	Secreted, extracellular space, extracellular matrix.
Synaptophysin-like protein 2	Q5VXT5	SYPL2	Membrane {ECO:0000250}; Multi-pass membrane protein {ECO:0000250}. Note=Triad junction, the junctional complex between the transverse tubule and the sarcoplasmic reticulum. {ECO:0000250}.
Voltage-dependent L-type calcium channel subunit beta-1	Q02641	CACNB1	Cell membrane, sarcolemma {ECO:0000305|PubMed:15615847}; Peripheral membrane protein {ECO:0000250|UniProtKB:P19517}; Cytoplasmic side {ECO:0000250|UniProtKB:P19517}. Cell membrane {ECO:0000305|PubMed:1309651}; Peripheral membrane protein {ECO:0000305|PubMed:1309651}.
Collagen alpha-1(I) chain	P02452	COL1A1	Secreted, extracellular space, extracellular matrix {ECO:0000255|PROSITE-ProRule:PRU00793}.
Histone H2A type 1-D	P20671	HIST1H2AD	Nucleus. Chromosome.
28S ribosomal protein S31, mitochondrial	Q92665	MRPS31	Mitochondrion {ECO:0000269|PubMed:11279123}.
C->U-editing enzyme APOBEC-2	Q9Y235	APOBEC2	0
Actin, cytoplasmic 1	P60709	ACTB	Cytoplasm, cytoskeleton {ECO:0000269|PubMed:11687588, ECO:0000269|PubMed:17289661}. Nucleus {ECO:0000269|PubMed:11687588, ECO:0000269|PubMed:29925947}. Note=Localized in cytoplasmic mRNP granules containing untranslated mRNAs. {ECO:0000269|PubMed:17289661}.
Glutamine--tRNA ligase	P47897	QARS	Cytoplasm, cytosol {ECO:0000269|PubMed:19289464}. Cytoplasm {ECO:0000269|PubMed:10791971, ECO:0000269|PubMed:24656866}.
Myomesin-2	P54296	MYOM2	Cytoplasm, myofibril, sarcomere, M line {ECO:0000250}.

Supplemental Table 1 Page 23. All proteins quantitated in study

Protein Name	Uniprot	Gene	Subcellular localization
ATP synthase subunit O, mitochondrial	P48047	ATP5PO	Mitochondrion {ECO:0000250}. Mitochondrion inner membrane {ECO:0000250}.
Peroxiredoxin-6	P30041	PRDX6	Cytoplasm {ECO:0000269|PubMed:16186110, ECO:0000269|PubMed:19188445, ECO:0000269|PubMed:9497358}. Lysosome {ECO:0000250|UniProtKB:O35244}. Note=Also found in lung secretory organelles (lamellar bodies). {ECO:0000250|UniProtKB:O35244}.
Dual specificity mitogen-activated protein kinase kinase 1	Q02750	MAP2K1	Cytoplasm, cytoskeleton, microtubule organizing center, centrosome {ECO:0000269|PubMed:14737111}. Cytoplasm, cytoskeleton, microtubule organizing center, spindle pole body {ECO:0000269|PubMed:14737111}. Cytoplasm {ECO:0000269|PubMed:10409742, ECO:0000269|PubMed:17101779}. Nucleus {ECO:0000269|PubMed:17101779}. Membrane {ECO:0000269|PubMed:10409742}; Peripheral membrane protein {ECO:0000269|PubMed:10409742}. Note=Localizes at centrosomes during prometaphase, midzone during anaphase and midbody during telophase/cytokinesis (PubMed:14737111). Membrane localization is probably regulated by its interaction with KSR1 (PubMed:10409742). {ECO:0000269|PubMed:10409742, ECO:0000269|PubMed:14737111}.
Troponin I, slow skeletal muscle	P19237	TNNI1	0
Glucose-6-phosphate isomerase	P06744	GPI	Cytoplasm {ECO:0000269|PubMed:11437381}. Secreted {ECO:0000269|PubMed:11437381}.
Hydroxysteroid dehydrogenase-like protein 2	Q6YN16	HSDL2	Peroxisome {ECO:0000269|PubMed:19703561}.
cAMP-dependent protein kinase type II-alpha regulatory subunit	P13861	PRKAR2A	Cytoplasm {ECO:0000269|PubMed:21423175}. Cell membrane {ECO:0000269|PubMed:21423175}. Note=Colocalizes with PJA2 in the cytoplasm and the cell membrane.
Troponin I, fast skeletal muscle	P48788	TNNI2	0
Thioredoxin-related transmembrane protein 2	Q9Y320	TMX2	Membrane {ECO:0000305}; Single-pass type I membrane protein {ECO:0000305}.
Fructose-bisphosphate aldolase C	P09972	ALDOC	0
Myosin-8	P13535	MYH8	Cytoplasm, myofibril. Note=Thick filaments of the myofibrils.
Fructose-1,6-bisphosphatase isozyme 2	O00757	FBP2	Cell junction {ECO:0000250}. Cytoplasm. Nucleus. Cytoplasm, myofibril, sarcomere, Z line. Note=In neonatal cardiomyocytes, distributed throughout the cytosol, accumulating in the intercalated disks which occur at the Z line of cardiomyocytes and connect adjacent cells, and also located in the nucleus; dissociates from the Z line following an increase in cytosolic Ca(2+) concentration (By similarity). In muscle precursor cells, localizes predominantly to the nucleus and to a lesser extent to the cytoplasm at the proliferative phase, while mainly localizing to the cytoplasm at the differentiation phase (By similarity). Colocalizes with ALDOA and alpha-actinin on both sides of the Z line of skeletal muscle; dissociates rapidly from the Z line following an increase in cytosolic Ca(2+) concentration. {ECO:0000250}.
Mitochondrial coenzyme A transporter SLC25A42	Q86VD7	SLC25A42	Mitochondrion inner membrane {ECO:0000269|PubMed:19429682}; Multi-pass membrane protein {ECO:0000269|PubMed:19429682}.

Supplemental Table 1 Page 24. All proteins quantitated in study

Protein Name	Uniprot	Gene	Subcellular localization
Mitochondrial import receptor subunit TOM70	O94826	TOMM70	Mitochondrion outer membrane {ECO:0000250}; Single-pass membrane protein {ECO:0000250}.
Gelsolin	P06396	GSN	Isoform 2: Cytoplasm, cytoskeleton.; Isoform 1: Secreted.
Single-stranded DNA-binding protein, mitochondrial	Q04837	SSBP1	Mitochondrion {ECO:0000269|PubMed:18063578}. Mitochondrion matrix, mitochondrion nucleoid {ECO:0000269|PubMed:18063578}.
Carbonic anhydrase 2	P00918	CA2	Cytoplasm {ECO:0000269|PubMed:15990874}. Cell membrane {ECO:0000269|PubMed:15990874}. Note=Colocalized with SLC26A6 at the surface of the cell membrane in order to form a bicarbonate transport metabolon. Displaced from the cytosolic surface of the cell membrane by PKC in phorbol myristate acetate (PMA)-induced cells.
Alpha-1-syntrophin	Q13424	SNTA1	Cell membrane, sarcolemma {ECO:0000250}; Peripheral membrane protein {ECO:0000250}; Cytoplasmic side {ECO:0000250}. Cell junction {ECO:0000250}. Cytoplasm, cytoskeleton {ECO:0000250}. Note=In skeletal muscle, it localizes at the cytoplasmic side of the sarcolemmal membrane and at neuromuscular junctions. {ECO:0000250}.
Hemoglobin subunit delta	P02042	HBD	0
Protein-L-isoaspartate(D-aspartate) O-methyltransferase	P22061	PCMT1	Cytoplasm.
Cadherin-13	P55290	CDH13	Cell membrane; Lipid-anchor, GPI-anchor.
Beta-enolase	P13929	ENO3	Cytoplasm. Note=Localized to the Z line. Some colocalization with CKM at M-band (By similarity). {ECO:0000250}.
ES1 protein homolog, mitochondrial	A0A0B4J2D5	GATD3B	Mitochondrion {ECO:0000305}.
MICOS complex subunit MIC13	Q5XKP0	MICOS13	Mitochondrion inner membrane {ECO:0000269|PubMed:25997101}; Single-pass membrane protein {ECO:0000255}. Note=Enriched at crista junctions. {ECO:0000269|PubMed:25997101}.
Mimecan	P20774	OGN	Secreted, extracellular space, extracellular matrix {ECO:0000250}.
Dihydrolipoyllysine-residue acetyltransferase component of pyruvate dehydrogenase complex, mitochondrial	P10515	DLAT	Mitochondrion matrix.
28S ribosomal protein S29, mitochondrial	P51398	DAP3	Mitochondrion {ECO:0000269|PubMed:11162496, ECO:0000269|PubMed:20563667}.
Protein disulfide-isomerase A6	Q15084	PDIA6	Endoplasmic reticulum lumen {ECO:0000269|PubMed:15466936}. Cell membrane {ECO:0000269|PubMed:15466936}. Melanosome {ECO:0000269|PubMed:12643545, ECO:0000269|PubMed:17081065}. Note=Identified by mass spectrometry in melanosome fractions from stage I to stage IV (PubMed:12643545). {ECO:0000269|PubMed:12643545}.
Beta-2-glycoprotein 1	P02749	APOH	Secreted.

Supplemental Table 1 Page 25. All proteins quantitated in study

Protein Name	Uniprot	Gene	Subcellular localization
Dolichyl-diphosphooligosaccharide--protein glycosyltransferase 48 kDa subunit	P39656	DDOST	Endoplasmic reticulum membrane {ECO:0000250|UniProtKB:Q29381}; Single-pass type I membrane protein {ECO:0000250|UniProtKB:Q29381}.
Peptidyl-prolyl cis-trans isomerase A	P62937	PPIA	Cytoplasm {ECO:0000269|PubMed:16527992}. Secreted {ECO:0000269|PubMed:16527992}. Note=Secretion occurs in response to oxidative stress in vascular smooth muscle through a vesicular secretory pathway that involves actin remodeling and myosin II activation, and mediates ERK1/2 activation.
Sarcoplasmic/endoplasmic reticulum calcium ATPase 1	O14983	ATP2A1	Endoplasmic reticulum membrane {ECO:0000250|UniProtKB:P04191}; Multi-pass membrane protein {ECO:0000250|UniProtKB:P04191}. Sarcoplasmic reticulum membrane {ECO:0000250|UniProtKB:P04191}; Multi-pass membrane protein {ECO:0000250|UniProtKB:P04191}.
Vitronectin	P04004	VTN	Secreted, extracellular space.
Pyruvate dehydrogenase E1 component subunit alpha, somatic form, mitochondrial	P08559	PDHA1	Mitochondrion matrix.
Synaptopodin 2-like protein	Q9H987	SYNPO2L	Cytoplasm, cytoskeleton {ECO:0000250}.
DnaJ homolog subfamily A member 3, mitochondrial	Q96EY1	DNAJA3	Mitochondrion matrix. Cytoplasm, cytosol {ECO:0000250}. Cell junction, synapse, postsynaptic cell membrane {ECO:0000250}; Peripheral membrane protein {ECO:0000250}. Note=Recruited to the postsynaptic cell membrane of the neuromuscular junction through interaction with MUSK. {ECO:0000250}.
Transmembrane protein 14C	Q9P0S9	TMEM14C	Mitochondrion membrane {ECO:0000250}; Multi-pass membrane protein {ECO:0000250}.
Cytochrome b-c1 complex subunit Rieske, mitochondrial	P47985	UQCRFS1	Mitochondrion inner membrane {ECO:0000250|UniProtKB:Q5ZLR5}; Single-pass membrane protein {ECO:0000250|UniProtKB:Q5ZLR5}.
Tropomyosin alpha-1 chain	P09493	TPM1	Cytoplasm, cytoskeleton {ECO:0000250|UniProtKB:P04692}. Note=Associates with F-actin stress fibers. {ECO:0000250|UniProtKB:P04692}.
Cysteine and glycine-rich protein 3	P50461	CSRP3	Nucleus {ECO:0000250|UniProtKB:P50463}. Cytoplasm {ECO:0000269|PubMed:18505755}. Cytoplasm, cytoskeleton {ECO:0000305}. Cytoplasm, myofibril, sarcomere, Z line {ECO:0000269|PubMed:24860983}. Cytoplasm, myofibril, sarcomere {ECO:0000269|PubMed:24934443}. Note=Nucleocytoplasmic shuttling protein. Mainly cytoplasmic. In the Z line, found associated with GLRX3 (By similarity). {ECO:0000250|UniProtKB:P50462, ECO:0000250|UniProtKB:P50463}.; Isoform 2: Cytoplasm, myofibril, sarcomere, Z line {ECO:0000269|PubMed:24860983}.
Ras-related protein Rap-1b	P61224	RAP1B	Cell membrane. Cytoplasm, cytosol. Cell junction. Note=May shuttle between plasma membrane and cytosol. Presence of KRIT1 and CDH5 is required for its localization to the cell junction.
Cytochrome b-c1 complex subunit 6, mitochondrial	P07919	UQCRH	Mitochondrion inner membrane {ECO:0000250|UniProtKB:P00127}.

Supplemental Table 1 Page 26. All proteins quantitated in study

Protein Name	Uniprot	Gene	Subcellular localization
COP9 signalosome complex subunit 3	Q9UNS2	COPS3	Cytoplasm {ECO:0000269|PubMed:9535219}. Nucleus {ECO:0000269|PubMed:9535219}.
Glycogen [starch] synthase, muscle	P13807	GYS1	0
Catalase	P04040	CAT	Peroxisome.
Vesicle-associated membrane protein-associated protein A	Q9P0L0	VAPA	Endoplasmic reticulum membrane {ECO:0000269|PubMed:10523508, ECO:0000269|PubMed:16143324, ECO:0000269|PubMed:19289470, ECO:0000269|PubMed:25447204}; Single-pass type IV membrane protein {ECO:0000269|PubMed:10523508, ECO:0000269|PubMed:19289470}. Cell membrane {ECO:0000269|PubMed:25447204}; Single-pass type IV membrane protein {ECO:0000305}. Cell junction, tight junction {ECO:0000269|PubMed:10523508}. Nucleus membrane {ECO:0000250|UniProtKB:Q9Z270}. Note=Present in the plasma membrane and in intracellular vesicles, together with SNARE proteins. May also associate with the cytoskeleton. Colocalizes with OCLN at the tight junction in polarized epithelial cells. {ECO:0000269|PubMed:10523508}.
ATP synthase subunit d, mitochondrial	O75947	ATP5PD	Mitochondrion. Mitochondrion inner membrane.
Thioredoxin-related transmembrane protein 1	Q9H3N1	TMX1	Membrane {ECO:0000305}; Single-pass type I membrane protein {ECO:0000305}. Endoplasmic reticulum membrane {ECO:0000305}; Single-pass type I membrane protein {ECO:0000305}. Note=Predominantly found in the endoplasmic reticulum. {ECO:0000269|PubMed:11152479}.
14-3-3 protein epsilon	P62258	YWHAE	Nucleus {ECO:0000269|PubMed:12917326}. Cytoplasm {ECO:0000269|PubMed:12917326}. Melanosome {ECO:0000269|PubMed:12042314, ECO:0000269|PubMed:17081065}. Note=Identified by mass spectrometry in melanosome fractions from stage I to stage IV. {ECO:0000269|PubMed:12042314, ECO:0000269|PubMed:17081065}.
Cathepsin D	P07339	CTSD	Lysosome. Melanosome. Secreted, extracellular space. Note=Identified by mass spectrometry in melanosome fractions from stage I to stage IV. In aortic samples, detected as an extracellular protein loosely bound to the matrix (PubMed:20551380). {ECO:0000269|PubMed:20551380}.
Myosin-3	P11055	MYH3	Cytoplasm, myofibril. Note=Thick filaments of the myofibrils.
Succinate--CoA ligase [ADP-forming] subunit beta, mitochondrial	Q9P2R7	SUCLA2	Mitochondrion {ECO:0000255|HAMAP-Rule:MF_03220, ECO:0000269|PubMed:15877282, ECO:0000269|PubMed:17287286, ECO:0000269|PubMed:25944712}.
Exportin-2	P55060	CSE1L	Cytoplasm {ECO:0000269|PubMed:9323134}. Nucleus {ECO:0000269|PubMed:9323134}. Note=Shuttles between the nucleus and the cytoplasm. {ECO:0000269|PubMed:9323134}.

Supplemental Table 1 Page 27. All proteins quantitated in study

Protein Name	Uniprot	Gene	Subcellular localization
Clathrin heavy chain 1	Q00610	CLTC	Cytoplasmic vesicle membrane {ECO:0000269|PubMed:17081065}; Peripheral membrane protein {ECO:0000269|PubMed:17081065}; Cytoplasmic side {ECO:0000269|PubMed:17081065}. Membrane, coated pit {ECO:0000269|PubMed:17081065}; Peripheral membrane protein {ECO:0000269|PubMed:17081065}; Cytoplasmic side {ECO:0000269|PubMed:17081065}. Melanosome {ECO:0000269|PubMed:17081065}. Cytoplasm, cytoskeleton, spindle {ECO:0000269|PubMed:15858577, ECO:0000269|PubMed:16968737, ECO:0000269|PubMed:23918938}. Note=Cytoplasmic face of coated pits and vesicles. Identified by mass spectrometry in melanosome fractions from stage I to stage IV. In complex with TACC3 and CKAP5 (forming the TACC3/ch-TOG/clathrin complex) localized to inter-microtubule bridges in mitotic spindles. {ECO:0000269|PubMed:25596274}.
[Pyruvate dehydrogenase [acetyl-transferring]]-phosphatase 1, mitochondrial	Q9P0J1	PDP1	Mitochondrion matrix {ECO:0000250}.
General vesicular transport factor p115	O60763	USO1	Cytoplasm, cytosol {ECO:0000269|PubMed:19454686, ECO:0000269|PubMed:9478999}. Golgi apparatus membrane {ECO:0000269|PubMed:19454686, ECO:0000269|PubMed:9478999}; Peripheral membrane protein {ECO:0000269|PubMed:19454686, ECO:0000269|PubMed:9478999}. Note=Recycles between the cytosol and the Golgi apparatus during interphase. During interphase, the phosphorylated form is found exclusively in cytosol; the unphosphorylated form is associated with Golgi apparatus membranes. {ECO:0000269|PubMed:19454686, ECO:0000269|PubMed:9478999}.
Ryanodine receptor 1	P21817	RYR1	Sarcoplasmic reticulum membrane {ECO:0000269|PubMed:7556644}; Multi-pass membrane protein {ECO:0000305}. Sarcoplasmic reticulum {ECO:0000250|UniProtKB:P11716}. Note=The number of predicted transmembrane domains varies between orthologs, but the 3D-structures show the presence of six transmembrane regions. Both N-terminus and C-terminus are cytoplasmic. {ECO:0000250|UniProtKB:P11716}.
Importin-5	O00410	IPO5	Cytoplasm. Nucleus. Nucleus, nucleolus. Note=Nucleus; nuclear rim. Found particularly in the nuclear rim and nucleolus.
Tubulin alpha-1B chain	P68363	TUBA1B	Cytoplasm, cytoskeleton.
Cytochrome c oxidase subunit 7A2, mitochondrial	P14406	COX7A2	Mitochondrion inner membrane.
Ras-related protein Rab-18	Q9NP72	RAB18	Cell membrane {ECO:0000305}; Lipid-anchor {ECO:0000305}; Cytoplasmic side {ECO:0000305}.
Methionine aminopeptidase 2	P50579	METAP2	Cytoplasm {ECO:0000255|HAMAP-Rule:MF_03175, ECO:0000269|PubMed:21537465}. Note=About 30% of expressed METAP2 associates with polysomes.
Tropomyosin beta chain	P07951	TPM2	Cytoplasm, cytoskeleton {ECO:0000250|UniProtKB:P58775}. Note=Associates with F-actin stress fibers. {ECO:0000250|UniProtKB:P58775}.
Cullin-associated NEDD8-dissociated protein 2	O75155	CAND2	Nucleus {ECO:0000250}.

Supplemental Table 1 Page 28. All proteins quantitated in study

Protein Name	Uniprot	Gene	Subcellular localization
Bcl-2-like protein 13	Q9BXK5	BCL2L13	Isoform 2: Mitochondrion membrane {ECO:0000305}; Single-pass membrane protein {ECO:0000305}. Nucleus {ECO:0000305}.; Isoform 1: Nucleus {ECO:0000305}.
Transmembrane protein 43	Q9BTV4	TMEM43	Endoplasmic reticulum {ECO:0000250}. Nucleus inner membrane; Multi-pass membrane protein. Note=Retained in the inner nuclear membrane through interaction with EMD and A- and B-lamins. The N- and C-termini are oriented towards the nucleoplasm. The majority of the hydrophilic domain resides in the endoplasmic reticulum lumen (By similarity). {ECO:0000250}.
Mitochondrial carnitine/acylcarnitine carrier protein	O43772	SLC25A20	Mitochondrion inner membrane; Multi-pass membrane protein.
Phosphatidylglycerophosphatase and protein-tyrosine phosphatase 1	Q8WUK0	PTPMT1	Mitochondrion inner membrane {ECO:0000250|UniProtKB:P0C089}; Peripheral membrane protein {ECO:0000250|UniProtKB:P0C089}; Matrix side {ECO:0000250|UniProtKB:P0C089}.
GTP:AMP phosphotransferase AK3, mitochondrial	Q9UIJ7	AK3	Mitochondrion matrix {ECO:0000255|HAMAP-Rule:MF_03169, ECO:0000269|PubMed:11485571}.
Smoothelin-like protein 1	A8MU46	SMTNL1	Cytoplasm, myofibril {ECO:0000269|PubMed:18310078}. Cytoplasm, myofibril, sarcomere, I band {ECO:0000250}. Cytoplasm, myofibril, sarcomere, M line {ECO:0000250}. Nucleus {ECO:0000250}. Note=Colocalizes with MYH2. In its unphosphorylated state, localizes to the cytoplasm (By similarity). Phosphorylation at Ser-301 promotes translocation to the nucleus (By similarity). {ECO:0000250}.
Inositol monophosphatase 2	O14732	IMPA2	0
Ras-related protein R-Ras2	P62070	RRAS2	Cell membrane {ECO:0000250}; Lipid-anchor {ECO:0000250}; Cytoplasmic side {ECO:0000250}. Note=Inner surface of plasma membrane possibly with attachment requiring acylation of the C-terminal cysteine (By similarity with RAS).
Myosin light chain kinase 2, skeletal/cardiac muscle	Q9H1R3	MYLK2	Cytoplasm. Note=Colocalizes with phosphorylated myosin light chain (RLCP) at filaments of the myofibrils.
Myomesin-3	Q5VTT5	MYOM3	Cytoplasm, myofibril, sarcomere, M line {ECO:0000250}.
ADP-ribosylation factor 1	P84077	ARF1	Golgi apparatus {ECO:0000269|PubMed:17555535}. Cytoplasm, perinuclear region {ECO:0000269|PubMed:17555535}. Cell junction, synapse, synaptosome {ECO:0000250}. Cell junction, synapse, postsynaptic cell membrane, postsynaptic density {ECO:0000250}. Membrane {ECO:0000305}; Lipid-anchor {ECO:0000305}. Golgi apparatus, trans-Golgi network membrane {ECO:0000250|UniProtKB:P84078}; Lipid-anchor {ECO:0000250|UniProtKB:P84078}.
LIM and cysteine-rich domains protein 1	Q9NZU5	LMCD1	Cytoplasm {ECO:0000250}. Nucleus {ECO:0000250}. Note=May shuttle between the cytoplasm and the nucleus. {ECO:0000250}.
Myosin-binding protein C, fast-type	Q14324	MYBPC2	0
Alpha-1-antitrypsin	P01009	SERPINA1	Secreted. Endoplasmic reticulum. Note=The S and Z allele are not secreted effectively and accumulate intracellularly in the endoplasmic reticulum.; Short peptide from AAT: Secreted, extracellular space, extracellular matrix.
Elongation factor 1-alpha 2	Q05639	EEF1A2	Nucleus {ECO:0000250}.

Supplemental Table 1 Page 29. All proteins quantitated in study

Protein Name	Uniprot	Gene	Subcellular localization
Delta-1-pyrroline-5-carboxylate dehydrogenase, mitochondrial	P30038	ALDH4A1	Mitochondrion matrix.
Prohibitin-2	Q99623	PHB2	Mitochondrion inner membrane {ECO:0000269|PubMed:20959514}. Cytoplasm {ECO:0000269|PubMed:19496786}. Nucleus {ECO:0000269|PubMed:19496786, ECO:0000269|PubMed:20959514}.
39S ribosomal protein L41, mitochondrial	Q8IXM3	MRPL41	Mitochondrion {ECO:0000269|PubMed:16024796, ECO:0000269|PubMed:25278503, ECO:0000269|PubMed:25838379, ECO:0000269|PubMed:28892042}.
Pyruvate dehydrogenase E1 component subunit beta, mitochondrial	P11177	PDHB	Mitochondrion matrix.
Myosin-1	P12882	MYH1	Cytoplasm, myofibril. Note=Thick filaments of the myofibrils.
NADH dehydrogenase [ubiquinone] 1 beta subcomplex subunit 9	Q9Y6M9	NDUFB9	Mitochondrion inner membrane {ECO:0000305|PubMed:12611891}; Peripheral membrane protein {ECO:0000305}; Matrix side {ECO:0000305}.
Histone H4	P62805	HIST1H4A; HIST1H4B; HIST1H4C; HIST1H4D; HIST1H4E; HIST1H4F; HIST1H4H; HIST1H4I; HIST1H4J; HIST1H4K; HIST1H4L; HIST2H4A; HIST2H4B; HIST4H4	Nucleus. Chromosome.
28S ribosomal protein S27, mitochondrial	Q92552	MRPS27	Cytoplasm {ECO:0000269|PubMed:28714366}. Mitochondrion {ECO:0000269|PubMed:22841715, ECO:0000269|PubMed:25838379}.
Lysosome-associated membrane glycoprotein 1	P11279	LAMP1	Cell membrane {ECO:0000250|UniProtKB:P05300}; Single-pass type I membrane protein {ECO:0000255}. Endosome membrane; Single-pass type I membrane protein {ECO:0000255}. Lysosome membrane {ECO:0000269|PubMed:16176980, ECO:0000269|PubMed:17897319}; Single-pass type I membrane protein {ECO:0000255}. Late endosome {ECO:0000269|PubMed:16176980}. Note=This protein shuttles between lysosomes, endosomes, and the plasma membrane (By similarity). Colocalizes with OSBPL1A at the late endosome (PubMed:16176980). {ECO:0000250|UniProtKB:P05300, ECO:0000269|PubMed:16176980, ECO:0000269|PubMed:17897319}.

Supplemental Table 1 Page 30. All proteins quantitated in study

Protein Name	Uniprot	Gene	Subcellular localization
Dynamin-1-like protein	O00429	DNM1L	Cytoplasm, cytosol. Golgi apparatus. Endomembrane system; Peripheral membrane protein. Mitochondrion outer membrane {ECO:0000269|PubMed:26122121, ECO:0000269|PubMed:27145208, ECO:0000269|PubMed:28969390}; Peripheral membrane protein. Peroxisome. Membrane, clathrin-coated pit {ECO:0000250}. Cytoplasmic vesicle, secretory vesicle, synaptic vesicle membrane {ECO:0000250}. Note=Mainly cytosolic. Translocated to the mitochondrial membrane through O-GlcNAcylation and interaction with FIS1. Recruited to the mitochondrial outer membrane by interaction with MIEF1. Colocalized with MARCH5 at mitochondrial membrane. Localizes to mitochondria at sites of division. Localizes to mitochondria following necrosis induction. Associated with peroxisomal membranes, partly recruited there by PEX11B. May also be associated with endoplasmic reticulum tubules and cytoplasmic vesicles and found to be perinuclear. In some cell types, localizes to the Golgi complex. Binds to phospholipid membranes.
Histone H2B type 1-B	P33778	HIST1H2BB	Nucleus. Chromosome.
Putative transferase CAF17, mitochondrial	Q5T440	IBA57	Mitochondrion {ECO:0000269|PubMed:22323289, ECO:0000269|PubMed:23462291}.
Aspartyl/asparaginyl beta-hydroxylase	Q12797	ASPH	Isoform 1: Endoplasmic reticulum membrane; Single-pass type II membrane protein {ECO:0000250|UniProtKB:Q28056}.; Isoform 4: Sarcoplasmic reticulum membrane; Single-pass type II membrane protein {ECO:0000305|PubMed:15485681}.; Isoform 8: Endoplasmic reticulum membrane; Single-pass type II membrane protein {ECO:0000269|PubMed:22586105}.
Myomesin-1	P52179	MYOM1	Cytoplasm, myofibril, sarcomere, M line {ECO:0000250}.
ADP/ATP translocase 3	P12236	SLC25A6	Mitochondrion inner membrane; Multi-pass membrane protein. Note=The complex formed with ARL2BP, ARL2 and SLC25A6 is expressed in mitochondria. {ECO:0000250}.
ATPase ASNA1	O43681	ASNA1	Cytoplasm. Endoplasmic reticulum. Nucleus, nucleolus.
Guanine nucleotide-binding protein G(I)/G(S)/G(T) subunit beta-2	P62879	GNB2	Cytoplasm, perinuclear region {ECO:0000269|PubMed:16498633}.
AMP deaminase 1	P23109	AMPD1	0
Peroxisomal multifunctional enzyme type 2	P51659	HSD17B4	Peroxisome.
Poly(rC)-binding protein 1	Q15365	PCBP1	Nucleus. Cytoplasm. Note=Loosely bound in the nucleus. May shuttle between the nucleus and the cytoplasm.
Alpha-aminoadipic semialdehyde dehydrogenase	P49419	ALDH7A1	Cytoplasm, cytosol {ECO:0000269|PubMed:20207735}. Nucleus {ECO:0000269|PubMed:20207735}.; Isoform 1: Mitochondrion {ECO:0000269|PubMed:20207735}.
UTP--glucose-1-phosphate uridylyltransferase	Q16851	UGP2	Cytoplasm {ECO:0000269|PubMed:8354390}.

Supplemental Table 1 Page 31. All proteins quantitated in study

Protein Name	Uniprot	Gene	Subcellular localization
Protein disulfide-isomerase	P07237	P4HB	Endoplasmic reticulum {ECO:0000269|PubMed:23475612}. Endoplasmic reticulum lumen {ECO:0000269|PubMed:10636893, ECO:0000269|PubMed:23475612}. Melanosome {ECO:0000269|PubMed:12643545, ECO:0000269|PubMed:17081065}. Cell membrane {ECO:0000269|PubMed:21670307}; Peripheral membrane protein {ECO:0000305}. Note=Highly abundant. In some cell types, seems to be also secreted or associated with the plasma membrane, where it undergoes constant shedding and replacement from intracellular sources (Probable). Localizes near CD4-enriched regions on lymphoid cell surfaces (PubMed:11181151). Identified by mass spectrometry in melanosome fractions from stage I to stage IV (PubMed:10636893). Colocalizes with MTTP in the endoplasmic reticulum (PubMed:23475612). {ECO:0000269|PubMed:10636893, ECO:0000269|PubMed:11181151, ECO:0000269|PubMed:23475612, ECO:0000305}.
Cytochrome c oxidase subunit 5B, mitochondrial	P10606	COX5B	Mitochondrion inner membrane.
Protein kinase C and casein kinase substrate in neurons protein 3	Q9UKS6	PACSIN3	Cytoplasm {ECO:0000269|PubMed:11179684}. Cell membrane {ECO:0000250}; Peripheral membrane protein {ECO:0000250}; Cytoplasmic side {ECO:0000250}. Note=Detected at the inner aspect of the plasma membrane in myotubes. {ECO:0000250}.
Glutamate dehydrogenase 1, mitochondrial	P00367	GLUD1	Mitochondrion matrix.
Ubiquitin-conjugating enzyme E2 N	P61088	UBE2N	Nucleus {ECO:0000269|PubMed:19340006}. Cytoplasm {ECO:0000269|PubMed:19340006}.
EH domain-containing protein 2	Q9NZN4	EHD2	Cell membrane {ECO:0000269|PubMed:17233914}; Peripheral membrane protein {ECO:0000250|UniProtKB:Q8BH64}; Cytoplasmic side {ECO:0000250|UniProtKB:Q8BH64}. Membrane, caveola {ECO:0000250|UniProtKB:Q8BH64}; Peripheral membrane protein {ECO:0000250|UniProtKB:Q8BH64}; Cytoplasmic side {ECO:0000250|UniProtKB:Q8BH64}. Endosome membrane {ECO:0000250|UniProtKB:Q4V8H8}; Peripheral membrane protein {ECO:0000250|UniProtKB:Q4V8H8}; Cytoplasmic side {ECO:0000250|UniProtKB:Q4V8H8}. Cytoplasm, cytosol {ECO:0000250|UniProtKB:Q8BH64}. Note=Colocalizes with GLUT4 in intracellular tubulovesicular structures that are associated with cortical F-actin. Colocalizes with FER1L5 at plasma membrane in myoblasts and myotubes. {ECO:0000250|UniProtKB:Q8BH64}.
Glycerol-3-phosphate dehydrogenase [NAD(+)], cytoplasmic	P21695	GPD1	Cytoplasm.
Perilipin-3	O60664	PLIN3	Cytoplasm {ECO:0000269|PubMed:15545278, ECO:0000269|PubMed:26357594, ECO:0000269|PubMed:9590177}. Endosome membrane {ECO:0000305}; Peripheral membrane protein {ECO:0000305}; Cytoplasmic side {ECO:0000305}. Lipid droplet {ECO:0000305}. Note=Membrane associated on endosomes. Detected in the envelope and the core of lipid bodies and in lipid sails. {ECO:0000305}.

Supplemental Table 1 Page 32. All proteins quantitated in study

Protein Name	Uniprot	Gene	Subcellular localization
Pentatricopeptide repeat domain-containing protein 3, mitochondrial	Q96EY7	PTCD3	Mitochondrion {ECO:0000269|PubMed:19427859, ECO:0000269|PubMed:23908630}.
Y-box-binding protein 3	P16989	YBX3	Cytoplasm. Nucleus.
CD59 glycoprotein	P13987	CD59	Cell membrane; Lipid-anchor, GPI-anchor. Secreted. Note=Soluble form found in a number of tissues.
Phosphoglycerate mutase 2	P15259	PGAM2	0
Peroxiredoxin-2	P32119	PRDX2	Cytoplasm {ECO:0000269|PubMed:9497357}.
Caveolin-3	P56539	CAV3	Golgi apparatus membrane {ECO:0000250}; Peripheral membrane protein {ECO:0000250}. Cell membrane {ECO:0000250|UniProtKB:P51638}; Peripheral membrane protein {ECO:0000250}. Membrane, caveola {ECO:0000250|UniProtKB:P51637}; Peripheral membrane protein {ECO:0000250}. Cell membrane, sarcolemma {ECO:0000250|UniProtKB:P51637}. Note=Potential hairpin-like structure in the membrane. Membrane protein of caveolae (By similarity). {ECO:0000250}.
Troponin T, fast skeletal muscle	P45378	TNNT3	0
Glyceraldehyde-3-phosphate dehydrogenase	P04406	GAPDH	Cytoplasm, cytosol {ECO:0000269|PubMed:12829261}. Nucleus {ECO:0000250}. Cytoplasm, perinuclear region {ECO:0000269|PubMed:12829261}. Membrane {ECO:0000269|PubMed:12829261}. Cytoplasm, cytoskeleton {ECO:0000250}. Note=Translocates to the nucleus following S-nitrosylation and interaction with SIAH1, which contains a nuclear localization signal (By similarity). Postnuclear and Perinuclear regions. {ECO:0000250}.
Thioredoxin-dependent peroxide reductase, mitochondrial	P30048	PRDX3	Mitochondrion {ECO:0000250|UniProtKB:P35705}. Cytoplasm {ECO:0000305|PubMed:12492477}. Early endosome {ECO:0000269|PubMed:15750338}. Note=Localizes to early endosomes in a RPS6KC1-dependent manner. {ECO:0000269|PubMed:15750338}.
Protein FAM162A	Q96A26	FAM162A	Membrane {ECO:0000305}; Single-pass membrane protein {ECO:0000305}. Mitochondrion {ECO:0000269|PubMed:15082785, ECO:0000269|PubMed:16698020}.
Dysferlin	O75923	DYSF	Cell membrane, sarcolemma; Single-pass type II membrane protein. Cytoplasmic vesicle membrane {ECO:0000250}; Single-pass type II membrane protein {ECO:0000250}. Cell membrane. Note=Colocalizes, during muscle differentiation, with BIN1 in the T-tubule system of myotubules and at the site of contact between two myotubes or a myoblast and a myotube. Wounding of myotubes led to its focal enrichment to the site of injury and to its relocalization in a Ca(2+)-dependent manner toward the plasma membrane. Colocalizes with AHNAK, AHNAK2 and PARVB at the sarcolemma of skeletal muscle. Detected on the apical plasma membrane of the syncytiotrophoblast. Reaches the plasmma membrane through a caveolin-independent mechanism. Retained by caveolin at the plasmma membrane (By similarity). Colocalizes, during muscle differentiation, with CACNA1S in the T-tubule system of myotubules (By similarity). Accumulates and colocalizes with fusion vesicles at the sarcolemma disruption sites (By similarity). {ECO:0000250}.

Supplemental Table 1 Page 33. All proteins quantitated in study

Protein Name	Uniprot	Gene	Subcellular localization
Protoporphyrinogen oxidase	P50336	PPOX	Mitochondrion inner membrane {ECO:0000250}; Peripheral membrane protein {ECO:0000250}; Intermembrane side {ECO:0000250}.
Electron transfer flavoprotein subunit alpha, mitochondrial	P13804	ETFA	Mitochondrion matrix {ECO:0000305}.
Isocitrate dehydrogenase [NAD] subunit beta, mitochondrial	O43837	IDH3B	Mitochondrion.
Dehydrogenase/reductase SDR family member 7	Q9Y394	DHRS7	0
Programmed cell death 6-interacting protein	Q8WUM4	PDCD6IP	Cytoplasm, cytosol {ECO:0000250|UniProtKB:Q9QZA2}. Melanosome {ECO:0000269|PubMed:17081065}. Cytoplasm, cytoskeleton, microtubule organizing center, centrosome {ECO:0000269|PubMed:17556548, ECO:0000269|PubMed:17853893}. Secreted, exosome {ECO:0000269|PubMed:22660413}. Cell junction, tight junction {ECO:0000250|UniProtKB:Q9WU78}. Midbody, Midbody ring {ECO:0000269|PubMed:17853893, ECO:0000269|PubMed:18641129}. Note=Identified by mass spectrometry in melanosome fractions from stage I to stage IV. Colocalized with CEP55 at centrosomes of non-dividing cells. Component of the actomyosin-tight junction complex (By similarity). PDCD6IP targeting to the midbody requires the interaction with CEP55 (PubMed:18641129). {ECO:0000250|UniProtKB:Q9QZA2, ECO:0000250|UniProtKB:Q9WU78, ECO:0000269|PubMed:17081065, ECO:0000269|PubMed:17556548, ECO:0000269|PubMed:17853893, ECO:0000269|PubMed:18641129}.
Methyltransferase-like protein 7A	Q9H8H3	METTL7A	Lipid droplet {ECO:0000269|PubMed:18477614, ECO:0000269|PubMed:19773358}. Endoplasmic reticulum {ECO:0000269|PubMed:19773358}. Membrane {ECO:0000269|PubMed:18477614}.
Peroxiredoxin-5, mitochondrial	P30044	PRDX5	Isoform Mitochondrial: Mitochondrion {ECO:0000269|PubMed:10521424, ECO:0000269|PubMed:10751410}.; Isoform Cytoplasmic+peroxisomal: Cytoplasm {ECO:0000269|PubMed:10514471, ECO:0000269|PubMed:10751410}. Peroxisome matrix {ECO:0000269|PubMed:10514471, ECO:0000269|PubMed:10521424, ECO:0000269|PubMed:10751410}. Note=Imported into peroxisomes via peroxisomal targeting signal 1 receptor PEX5. {ECO:0000269|PubMed:10514471}.
Acylglycerol kinase, mitochondrial	Q53H12	AGK	Mitochondrion inner membrane {ECO:0000269|PubMed:15939762, ECO:0000269|PubMed:16269826, ECO:0000269|PubMed:28712724, ECO:0000269|PubMed:28712726}; Peripheral membrane protein {ECO:0000269|PubMed:28712724}. Mitochondrion intermembrane space {ECO:0000269|PubMed:28712724, ECO:0000269|PubMed:28712726}. Note=Localizes in the mitochondrion intermembrane space, where it associates with the inner membrane (PubMed:28712724). It is unclear whether the N-terminal hydrophobic region forms a transmembrane region or associates with the membrane without crossing it (PubMed:28712724, PubMed:28712726). {ECO:0000269|PubMed:28712724, ECO:0000269|PubMed:28712726}.

Supplemental Table 1 Page 34. All proteins quantitated in study

Protein Name	Uniprot	Gene	Subcellular localization
ATP synthase F(0) complex subunit B1, mitochondrial	P24539	ATP5PB	Mitochondrion. Mitochondrion inner membrane.
Annexin A5	P08758	ANXA5	0
Glycogen debranching enzyme	P35573	AGL	Cytoplasm {ECO:0000269|PubMed:17908927}. Note=Under glycogenolytic conditions localizes to the nucleus.
GDH/6PGL endoplasmic bifunctional protein	O95479	H6PD	Endoplasmic reticulum lumen. Note=Microsomes, endoplasmic reticulum lumen.
PDZ and LIM domain protein 5	Q96HC4	PDLIM5	Cell junction, synapse, postsynaptic cell membrane, postsynaptic density {ECO:0000250}. Cell junction, synapse, synaptosome {ECO:0000250}. Cytoplasm {ECO:0000250}. Note=Detected both at presynaptic and postsynaptic sites. {ECO:0000250}.
Synemin	O15061	SYNM	Cytoplasm, cytoskeleton. Cell junction, adherens junction. Note=There are at least two distinct SYNM subpopulations, one in which SYMN interacts with DES within the Z-lines, and another in which it interacts with both DTNA and DES at the costamere.
Eukaryotic translation initiation factor 4 gamma 2	P78344	EIF4G2	0
Ras-related protein Rab-7a	P51149	RAB7A	Cytoplasmic vesicle, phagosome membrane {ECO:0000269|PubMed:12944476, ECO:0000269|PubMed:21255211}; Peripheral membrane protein {ECO:0000305}; Cytoplasmic side {ECO:0000305}. Late endosome membrane {ECO:0000269|PubMed:12944476, ECO:0000269|PubMed:14617358, ECO:0000269|PubMed:16176980, ECO:0000269|PubMed:20028791}; Peripheral membrane protein {ECO:0000305}; Cytoplasmic side {ECO:0000305}. Lysosome membrane {ECO:0000269|PubMed:12944476, ECO:0000269|PubMed:20028791}; Peripheral membrane protein {ECO:0000305}; Cytoplasmic side {ECO:0000305}. Melanosome membrane {ECO:0000269|PubMed:12643545, ECO:0000269|PubMed:17081065}; Peripheral membrane protein {ECO:0000305}; Cytoplasmic side {ECO:0000305}. Cytoplasmic vesicle, autophagosome membrane {ECO:0000269|PubMed:20028791}; Peripheral membrane protein {ECO:0000305}; Cytoplasmic side {ECO:0000305}. Lipid droplet {ECO:0000250|UniProtKB:P51150}. Endosome membrane {ECO:0000269|PubMed:22431521}. Note=Colocalizes with OSBPL1A at the late endosome (PubMed:16176980). Found in the ruffled border (a late endosomal-like compartment in the plasma membrane) of bone-resorbing osteoclasts. Recruited to phagosomes containing S.aureus or Mycobacterium (PubMed:21255211). Lipid droplet localization is increased upon ADRB2 stimulation (By similarity). {ECO:0000250|UniProtKB:P51150, ECO:0000269|PubMed:16176980, ECO:0000269|PubMed:21255211}.

Supplemental Table 1 Page 35. All proteins quantitated in study

Protein Name	Uniprot	Gene	Subcellular localization
Stromal interaction molecule 1	Q13586	STIM1	Cell membrane; Single-pass type I membrane protein {ECO:0000269|PubMed:11004585, ECO:0000269|PubMed:16005298, ECO:0000269|PubMed:16208375, ECO:0000269|PubMed:18854159, ECO:0000269|PubMed:19249086, ECO:0000269|PubMed:27185316, ECO:0000269|PubMed:28219928}. Endoplasmic reticulum membrane; Single-pass type I membrane protein {ECO:0000269|PubMed:16005298, ECO:0000269|PubMed:16208375, ECO:0000269|PubMed:18854159, ECO:0000269|PubMed:19249086, ECO:0000269|PubMed:26322679, ECO:0000269|PubMed:27185316}. Cytoplasm, cytoskeleton {ECO:0000269|PubMed:19632184}. Sarcoplasmic reticulum {ECO:0000269|PubMed:25326555}. Note=Translocates from the endoplasmic reticulum to the cell membrane in response to a depletion of intracellular calcium and is detected at punctae corresponding to junctions between the endoplasmic reticulum and the cell membrane (PubMed:19249086, PubMed:16005298, PubMed:16208375, PubMed:18854159). Associated with the microtubule network at the growing distal tip of microtubules (PubMed:19632184). Colocalizes with ORAI1 at the cell membrane (PubMed:27185316). Colocalizes preferentially with CASQ1 at endoplasmic reticulum in response to a depletion of intracellular calcium (PubMed:27185316). {ECO:0000269|PubMed:16005298, ECO:0000269|PubMed:16208375, ECO:0000269|PubMed:18854159, ECO:0000269|PubMed:19249086, ECO:0000269|PubMed:19632184, ECO:0000269|PubMed:27185316}.
Carbonyl reductase family member 4	Q8N4T8	CBR4	Mitochondrion matrix {ECO:0000269|PubMed:19000905, ECO:0000269|PubMed:19571038}.
Calcium-binding mitochondrial carrier protein Aralar1	O75746	SLC25A12	Mitochondrion inner membrane {ECO:0000269|PubMed:9722566}; Multi-pass membrane protein {ECO:0000269|PubMed:9722566}.
Atypical kinase COQ8A, mitochondrial	Q8NI60	COQ8A	Mitochondrion {ECO:0000269|PubMed:11888884, ECO:0000269|PubMed:25498144}. Membrane {ECO:0000255}; Single-pass membrane protein {ECO:0000255, ECO:0000305|PubMed:25216398}.
Membrane primary amine oxidase	Q16853	AOC3	Cell membrane {ECO:0000269|PubMed:9653080}; Single-pass type II membrane protein {ECO:0000269|PubMed:9653080}.
PGC-1 and ERR-induced regulator in muscle protein 1	Q5SV97	PERM1	Cytoplasm {ECO:0000250|UniProtKB:Q149B8}. Nucleus {ECO:0000250|UniProtKB:Q149B8}. Note=Shows a nuclear localization in the presence of PPARGC1A. {ECO:0000250|UniProtKB:Q149B8}.
Epimerase family protein SDR39U1	Q9NRG7	SDR39U1	0

Supplemental Table 1 Page 36. All proteins quantitated in study

Protein Name	Uniprot	Gene	Subcellular localization
Junctophilin-2	Q9BR39	JPH2	Junctophilin-2: Cell membrane {ECO:0000250|UniProtKB:Q9ET78}; Peripheral membrane protein {ECO:0000250|UniProtKB:Q9ET78}. Sarcoplasmic reticulum membrane {ECO:0000250|UniProtKB:Q9ET78}; Single-pass type IV membrane protein {ECO:0000250|UniProtKB:Q9ET78}. Endoplasmic reticulum membrane {ECO:0000250|UniProtKB:Q9ET78}; Single-pass type IV membrane protein {ECO:0000250|UniProtKB:Q9ET78}. Note=The transmembrane domain is anchored in sarcoplasmic reticulum membrane, while the N-terminal part associates with the plasma membrane. In heart cells, it predominantly associates along Z lines within myocytes. In skeletal muscle, it is specifically localized at the junction of A and I bands. {ECO:0000250|UniProtKB:Q9ET78}.; Junctophilin-2 N-terminal fragment: Nucleus {ECO:0000269|PubMed:30409805}. Note=Accumulates in the nucleus of stressed hearts. {ECO:0000250|UniProtKB:Q9ET78}.
ATP synthase-coupling factor 6, mitochondrial	P18859	ATP5PF	Mitochondrion. Mitochondrion inner membrane.
Creatine kinase S-type, mitochondrial	P17540	CKMT2	Mitochondrion inner membrane; Peripheral membrane protein; Intermembrane side.
Mycophenolic acid acyl-glucuronide esterase, mitochondrial	Q9NUJ1	ABHD10	Mitochondrion {ECO:0000305}.
Trifunctional enzyme subunit alpha, mitochondrial	P40939	HADHA	Mitochondrion.
Isovaleryl-CoA dehydrogenase, mitochondrial	P26440	IVD	Mitochondrion matrix.
Mitochondrial chaperone BCS1	Q9Y276	BCS1L	Mitochondrion inner membrane {ECO:0000269|PubMed:18628306, ECO:0000269|PubMed:9878253}; Single-pass membrane protein {ECO:0000269|PubMed:18628306, ECO:0000269|PubMed:9878253}.
ATP-binding cassette sub-family B member 7, mitochondrial	O75027	ABCB7	Mitochondrion inner membrane {ECO:0000305}; Multi-pass membrane protein {ECO:0000255|PROSITE-ProRule:PRU00441}.
Carnitine O-palmitoyltransferase 2, mitochondrial	P23786	CPT2	Mitochondrion inner membrane; Peripheral membrane protein; Matrix side.
Calcium/calmodulin-dependent protein kinase type II subunit beta	Q13554	CAMK2B	Cytoplasm, cytoskeleton {ECO:0000269|PubMed:21529938}. Cytoplasm, cytoskeleton, microtubule organizing center, centrosome {ECO:0000250}. Sarcoplasmic reticulum membrane {ECO:0000269|PubMed:21529938}; Peripheral membrane protein {ECO:0000269|PubMed:21529938}; Cytoplasmic side {ECO:0000269|PubMed:21529938}. Cell junction, synapse {ECO:0000250|UniProtKB:P08413}. Note=In slow-twitch muscle, evenly distributed between longitudinal SR and junctional SR.

Supplemental Table 1 Page 37. All proteins quantitated in study

Protein Name	Uniprot	Gene	Subcellular localization
Catechol O-methyltransferase	P21964	COMT	Isoform Soluble: Cytoplasm.; Isoform Membrane-bound: Cell membrane; Single-pass type II membrane protein; Extracellular side {ECO:0000269|PubMed:21846718}.
Kelch-like protein 40	Q2TBA0	KLHL40	Cytoplasm {ECO:0000250|UniProtKB:Q9D783}. Cytoplasm, myofibril, sarcomere, A band {ECO:0000269|PubMed:23746549}. Cytoplasm, myofibril, sarcomere, I band {ECO:0000250|UniProtKB:Q9D783}.
Glycerol-3-phosphate dehydrogenase, mitochondrial	P43304	GPD2	Mitochondrion.
Myosin light chain 3	P08590	MYL3	0
Filamin-C	Q14315	FLNC	Cytoplasm {ECO:0000269|PubMed:11038172, ECO:0000269|PubMed:25351925}. Membrane {ECO:0000269|PubMed:11038172}; Peripheral membrane protein {ECO:0000269|PubMed:11038172}. Cytoplasm, cytoskeleton {ECO:0000269|PubMed:11038172}. Cytoplasm, myofibril, sarcomere, Z line {ECO:0000269|PubMed:11038172}. Note=A small amount localizes at membranes. In striated muscle cells, it predominantly localizes in myofibrillar Z lines, while a minor fraction localizes with subsarcolemme. Targeting to developing and mature Z lines is mediated by the intradomain insert.
Isobutyryl-CoA dehydrogenase, mitochondrial	Q9UKU7	ACAD8	Mitochondrion {ECO:0000269|PubMed:11013134}.
Protein NDRG2	Q9UN36	NDRG2	Cytoplasm. Cytoplasm, perinuclear region. Cell projection, growth cone {ECO:0000250}. Note=In neurons, seems to concentrate at axonal growth cone. Perinuclear in neurons (By similarity). {ECO:0000250}.
NADH dehydrogenase [ubiquinone] 1 alpha subcomplex subunit 11	Q86Y39	NDUFA11	Mitochondrion inner membrane {ECO:0000305|PubMed:12611891}; Multi-pass membrane protein {ECO:0000255}; Matrix side {ECO:0000305}.
Alpha-actinin-1	P12814	ACTN1	Cytoplasm, cytoskeleton {ECO:0000269|PubMed:24069336, ECO:0000269|PubMed:7750553}. Cytoplasm, myofibril, sarcomere, Z line {ECO:0000269|PubMed:7750553}. Cell membrane {ECO:0000250|UniProtKB:Q9Z1P2}. Cell junction {ECO:0000250|UniProtKB:Q9Z1P2}. Cell projection, ruffle {ECO:0000250|UniProtKB:Q7TPR4}. Note=Colocalizes with MYOZ2 and PPP3CA at the Z-line of heart and skeletal muscle. Colocalizes with PSD in membrane ruffles and central reticular structures. {ECO:0000250|UniProtKB:Q7TPR4}.
Syntaxin-binding protein 3	O00186	STXBP3	Cytoplasm, cytosol {ECO:0000269|PubMed:10194441}. Cell membrane {ECO:0000269|PubMed:10194441}. Note=In platelets, predominantly cytosolic. Low amounts membrane-associated.
Cytochrome b5 type B	O43169	CYB5B	Mitochondrion outer membrane {ECO:0000250|UniProtKB:P04166}.

Supplemental Table 1 Page 38. All proteins quantitated in study

Protein Name	Uniprot	Gene	Subcellular localization
Lysine--tRNA ligase	Q15046	KARS	Isoform Cytoplasmic: Cytoplasm, cytosol {ECO:0000269|PubMed:10952987, ECO:0000269|PubMed:19289464, ECO:0000269|PubMed:23159739, ECO:0000269|PubMed:28887846}. Cytoplasm {ECO:0000269|PubMed:15220430}. Nucleus {ECO:0000269|PubMed:15220430, ECO:0000269|PubMed:23159739}. Cell membrane {ECO:0000269|PubMed:15220430}; Peripheral membrane protein {ECO:0000269|PubMed:15220430}. Secreted {ECO:0000269|PubMed:15851690}. Note=Secretion is induced by TNF-alpha (PubMed:15851690). Cytosolic in quiescent mast cells. Translocates into the nucleus in response to mast cell activation by immunoglobulin E (PubMed:23159739). {ECO:0000269|PubMed:15851690, ECO:0000269|PubMed:23159739}.; Isoform Mitochondrial: Mitochondrion {ECO:0000269|PubMed:10952987}.
Peptidyl-prolyl cis-trans isomerase FKBP1A	P62942	FKBP1A	Cytoplasm, cytosol {ECO:0000269|PubMed:1701173}. Sarcoplasmic reticulum membrane {ECO:0000250|UniProtKB:P62943}; Peripheral membrane protein {ECO:0000250|UniProtKB:P62943}; Cytoplasmic side {ECO:0000250|UniProtKB:P62943}.
Protein disulfide-isomerase A3	P30101	PDIA3	Endoplasmic reticulum {ECO:0000269|PubMed:23826168}. Endoplasmic reticulum lumen {ECO:0000250}. Melanosome {ECO:0000269|PubMed:12643545, ECO:0000269|PubMed:17081065}. Note=Identified by mass spectrometry in melanosome fractions from stage I to stage IV (PubMed:12643545). {ECO:0000269|PubMed:12643545}.
D-beta-hydroxybutyrate dehydrogenase, mitochondrial	Q02338	BDH1	Mitochondrion inner membrane {ECO:0000250|UniProtKB:Q02337}. Mitochondrion matrix {ECO:0000250|UniProtKB:Q02337}.
Ubiquitin-60S ribosomal protein L40	P62987	UBA52	Ubiquitin: Cytoplasm {ECO:0000250}. Nucleus {ECO:0000250}.; 60S ribosomal protein L40: Cytoplasm {ECO:0000250}.
Reticulon-4	Q9NQC3	RTN4	Isoform A: Endoplasmic reticulum membrane {ECO:0000269|PubMed:25612671, ECO:0000269|PubMed:26906412, ECO:0000269|PubMed:27619977, ECO:0000269|PubMed:27786289}; Multi-pass membrane protein {ECO:0000255}. Cell membrane; Multi-pass membrane protein {ECO:0000255}; Cytoplasmic side {ECO:0000269|PubMed:27786289}. Note=Anchored to the membrane of the endoplasmic reticulum (ER) through 2 putative transmembrane domains. Localizes throughout the ER tubular network (PubMed:27619977). Co-localizes with TMEM33 at the ER sheets. {ECO:0000269|PubMed:25612671, ECO:0000269|PubMed:27619977}.; Isoform B: Endoplasmic reticulum membrane {ECO:0000269|PubMed:27786289}; Multi-pass membrane protein {ECO:0000255}. Cell membrane {ECO:0000269|PubMed:15034570}; Multi-pass membrane protein {ECO:0000255}; Extracellular side {ECO:0000269|PubMed:15034570}. Cell junction {ECO:0000269|PubMed:21183689}. Note=Mainly located on endoplasmic reticulum tubules and sheet edges (PubMed:27786289). Upon ICAM1 engagement, redistributed toward endothelial junctions where interacts with CDH5 (PubMed:21183689). {ECO:0000269|PubMed:21183689, ECO:0000269|PubMed:27786289}.; Isoform C: Endoplasmic reticulum membrane {ECO:0000269|PubMed:25612671}; Multi-pass membrane protein {ECO:0000255}.

Supplemental Table 1 Page 39. All proteins quantitated in study

Protein Name	Uniprot	Gene	Subcellular localization
Long-chain-fatty-acid--CoA ligase 3	O95573	ACSL3	Mitochondrion outer membrane {ECO:0000250}; Single-pass type III membrane protein {ECO:0000250}. Peroxisome membrane {ECO:0000250}; Single-pass type III membrane protein {ECO:0000250}. Microsome membrane {ECO:0000250}; Single-pass type III membrane protein {ECO:0000250}. Endoplasmic reticulum membrane {ECO:0000250}; Single-pass type III membrane protein {ECO:0000250}.
Triadin	Q13061	TRDN	Cell membrane {ECO:0000269|PubMed:22422768}. Sarcoplasmic reticulum membrane {ECO:0000269|PubMed:22422768, ECO:0000269|PubMed:24325401}; Single-pass type II membrane protein {ECO:0000269|PubMed:22422768, ECO:0000269|PubMed:24325401}.
Trifunctional enzyme subunit beta, mitochondrial	P55084	HADHB	Mitochondrion {ECO:0000269|PubMed:21527675}. Mitochondrion inner membrane {ECO:0000269|PubMed:21527675}. Mitochondrion outer membrane {ECO:0000269|PubMed:21527675}. Endoplasmic reticulum {ECO:0000269|PubMed:21527675}.
CD81 antigen	P60033	CD81	Cell membrane {ECO:0000269|PubMed:1383329, ECO:0000269|PubMed:1695320, ECO:0000269|PubMed:20237408, ECO:0000269|PubMed:22307619, ECO:0000269|PubMed:2398277}; Multi-pass membrane protein {ECO:0000255}. Basolateral cell membrane {ECO:0000269|PubMed:20375010}; Multi-pass membrane protein {ECO:0000255}. Note=Associates with CLDN1 and the CLDN1-CD81 complex localizes to the basolateral cell membrane. {ECO:0000269|PubMed:20375010}.
Acetyl-CoA acetyltransferase, mitochondrial	P24752	ACAT1	Mitochondrion.
Transmembrane protein 143	Q96AN5	TMEM143	Membrane {ECO:0000305}; Multi-pass membrane protein {ECO:0000305}.
Nesprin-2	Q8WXH0	SYNE2	Nucleus outer membrane {ECO:0000305}; Single-pass type IV membrane protein {ECO:0000305}; Cytoplasmic side {ECO:0000305}. Sarcoplasmic reticulum membrane {ECO:0000305|PubMed:15671068}; Single-pass type IV membrane protein {ECO:0000305}. Cell membrane {ECO:0000305}; Single-pass membrane protein {ECO:0000305}. Cytoplasm, cytoskeleton. Mitochondrion. Nucleus, nucleoplasm. Cytoplasm, myofibril, sarcomere, Z line {ECO:0000305|PubMed:15671068}. Note=Different isoform patterns are found in the different compartments of the cell. The isoforms having the C-terminal transmembrane span can be found in several organellar membranes like the nuclear envelope, the sarcoplasmic reticulum of myoblasts, or the lamellipodia and focal adhesions at the cell membrane. The largest part of the outer nuclear membrane-associated protein is cytoplasmic, while its C-terminal part is associated with the nuclear envelope, most probably the outer nuclear membrane. Remains associated with the nuclear envelope during its breakdown in mitotic cells. Shorter soluble isoforms can be found in the cytoplasm and within the nucleus.; Isoform 8: Cell junction, focal adhesion {ECO:0000269|PubMed:22768332}. Note=In U2OS cells. {ECO:0000269|PubMed:22768332}.
NADH dehydrogenase [ubiquinone] 1 beta subcomplex subunit 10	O96000	NDUFB10	Mitochondrion inner membrane {ECO:0000305|PubMed:12611891}; Peripheral membrane protein {ECO:0000305}; Matrix side {ECO:0000305}.

Supplemental Table 1 Page 40. All proteins quantitated in study

Protein Name	Uniprot	Gene	Subcellular localization
Histone-lysine N-methyltransferase SMYD1	Q8NB12	SMYD1	Cytoplasm {ECO:0000250}. Nucleus {ECO:0000250}.
Ras-related protein Rab-2A	P61019	RAB2A	Endoplasmic reticulum-Golgi intermediate compartment membrane {ECO:0000269|PubMed:17081065}; Lipid-anchor {ECO:0000269|PubMed:17081065}. Melanosome {ECO:0000269|PubMed:17081065}. Endoplasmic reticulum membrane {ECO:0000305}; Lipid-anchor {ECO:0000305}. Golgi apparatus membrane {ECO:0000305}; Lipid-anchor {ECO:0000305}. Note=Identified by mass spectrometry in melanosome fractions from stage I to stage IV.
L-2-hydroxyglutarate dehydrogenase, mitochondrial	Q9H9P8	L2HGDH	Mitochondrion {ECO:0000269|PubMed:16005139}.
Long-chain fatty acid transport protein 1	Q6PCB7	SLC27A1	Cell membrane {ECO:0000250|UniProtKB:Q60714}; Single-pass membrane protein {ECO:0000250|UniProtKB:Q60714}. Endomembrane system {ECO:0000250|UniProtKB:Q60714}; Single-pass membrane protein {ECO:0000250|UniProtKB:Q60714}. Cytoplasm {ECO:0000250|UniProtKB:Q60714}. Note=Plasma membrane and intracellular membranes, at least in adipocytes. In adipocytes, but not myocytes, insulin via the mTORC1 signaling pathway induces a rapid translocation of SLC27A1 from intracellular compartments to the plasma membrane, paralleled by increased LCFA uptake. Insulin-dependent translocation from the cytoplasm to the cell membrane is regulated by EPRS. Predominantly cytoplasmic in myocytes. {ECO:0000250|UniProtKB:Q60714}.
Serum albumin	P02768	ALB	Secreted.
Dehydrogenase/reductase SDR family member 7B	Q6IAN0	DHRS7B	Endoplasmic reticulum membrane {ECO:0000250}; Single-pass type II membrane protein {ECO:0000250}.
Mitochondrial import receptor subunit TOM40 homolog	O96008	TOMM40	Mitochondrion outer membrane {ECO:0000269|PubMed:15644312}; Multi-pass membrane protein {ECO:0000255}.

Supplemental Table 1 Page 41. All proteins quantitated in study

Protein Name	Uniprot	Gene	Subcellular localization
Synaptopodin-2	Q9UMS6	SYNPO2	Nucleus {ECO:0000250|UniProtKB:Q91YE8}. Cytoplasm {ECO:0000250|UniProtKB:Q91YE8}. Cytoplasm, cytoskeleton {ECO:0000269|PubMed:24005909}. Cytoplasm, myofibril, sarcomere, Z line {ECO:0000269|PubMed:20554076}. Cell junction, focal adhesion {ECO:0000269|PubMed:20554076}. Note=Shuttles between the nucleus and the cytoplasm in a differentiation-dependent and stress-induced fashion. In undifferentiated myoblasts strongly expressed in the nucleus, after induction of myotube differentiation is located to both nucleus and cytoplasm along acting filaments, and in differentiated myotubes is located at the Z lines. Upon stress redistributes from cytoplasm of myoblasts and myotubes to the nucleus. Nuclear import is KPNA2-dependent and promoted by phosphorylation by PKA and/or CaMK2, and inhibition of calcineurin. The nuclear export is XPO1-dependent (By similarity). Localized in a fiber-like pattern, partly overlapping with filamentous actin (PubMed:18371299). {ECO:0000250|UniProtKB:Q91YE8, ECO:0000269|PubMed:18371299}.; Isoform 1: Cytoplasm, cytoskeleton {ECO:0000269|PubMed:24005909}. Note=Localizes to induced actin bundles with contiguous staining. {ECO:0000269|PubMed:24005909}.; Isoform 2: Cytoplasm, cytoskeleton {ECO:0000269|PubMed:24005909}. Note=Localizes to induced actin bundles with punctuate staining. {ECO:0000269|PubMed:24005909}.; Isoform 3: Cytoplasm, cytoskeleton {ECO:0000269|PubMed:24005909}. Note=Localizes to induced irregular actin bundles with contiguous and punctuated staining. {ECO:0000269|PubMed:24005909}.; Isoform 4: Cytoplasm, cytoskeleton {ECO:0000269|PubMed:24005909}. Note=Localizes to induced actin bundles with punctuate staining. {ECO:0000269|PubMed:24005909}.; Isoform 5: Cytoplasm, cytoskeleton {ECO:0000269|PubMed:24005909}. Note=Localizes to induced actin bundles with contiguous staining. {ECO:0000269|PubMed:24005909}.
Lamin-B2	Q03252	LMNB2	Nucleus inner membrane; Lipid-anchor; Nucleoplasmic side.
CDGSH iron-sulfur domain-containing protein 1	Q9NZ45	CISD1	Mitochondrion outer membrane {ECO:0000269|PubMed:17376863, ECO:0000269|PubMed:17766439, ECO:0000269|PubMed:17905743, ECO:0000269|PubMed:18047834}; Single-pass type III membrane protein {ECO:0000269|PubMed:17376863, ECO:0000269|PubMed:17766439, ECO:0000269|PubMed:17905743, ECO:0000269|PubMed:18047834}.
Glycophorin-A	P02724	GYPA	Cell membrane {ECO:0000269|PubMed:11402026}; Single-pass type I membrane protein {ECO:0000269|PubMed:11402026}. Note=Appears to be colocalized with SLC4A1.
NLR family member X1	Q86UT6	NLRX1	Mitochondrion outer membrane {ECO:0000269|PubMed:18200010, ECO:0000269|PubMed:18219313, ECO:0000269|PubMed:27393910}.
3-hydroxyacyl-CoA dehydrogenase type-2	Q99714	HSD17B10	Mitochondrion {ECO:0000269|PubMed:12917011, ECO:0000269|PubMed:18984158}.
Succinate dehydrogenase [ubiquinone] iron-sulfur subunit, mitochondrial	P21912	SDHB	Mitochondrion inner membrane; Peripheral membrane protein; Matrix side.

Supplemental Table 1 Page 42. All proteins quantitated in study

Protein Name	Uniprot	Gene	Subcellular localization
Myosin regulatory light chain 2, skeletal muscle isoform	Q96A32	MYLPF	0
NADH-ubiquinone oxidoreductase chain 4	P03905	MT-ND4	Mitochondrion membrane {ECO:0000250}; Multi-pass membrane protein {ECO:0000250}.
Fermitin family homolog 2	Q96AC1	FERMT2	Cytoplasm. Cytoplasm, cell cortex. Cytoplasm, cytoskeleton. Cytoplasm, cytoskeleton, stress fiber. Cell junction, focal adhesion. Membrane; Peripheral membrane protein; Cytoplasmic side. Cell projection, lamellipodium membrane; Peripheral membrane protein; Cytoplasmic side. Nucleus. Cytoplasm, myofibril, sarcomere, I band {ECO:0000250}. Cell surface {ECO:0000250}. Note=Colocalizes with actin stress fibers at cell-ECM focal adhesion sites. Colocalizes with ITGB3 at lamellipodia at the leading edge of spreading cells. Binds to membranes that contain phosphatidylinositides.
Plasma membrane calcium-transporting ATPase 1	P20020	ATP2B1	Cell membrane {ECO:0000269|PubMed:18029012}; Multi-pass membrane protein {ECO:0000255}. Basolateral cell membrane {ECO:0000250|UniProtKB:G5E829}. Cell junction, synapse {ECO:0000250|UniProtKB:G5E829}. Note=Colocalizes with SV2A in photoreceptor synaptic terminals. Colocalizes with NPTN to the immunological synapse. Colocalizes with EPB41 to the basolateral membrane in enterocyte. {ECO:0000250|UniProtKB:G5E829}.
Sarcoplasmic reticulum histidine-rich calcium-binding protein	P23327	HRC	Sarcoplasmic reticulum lumen.
Guanine nucleotide-binding protein G(I)/G(S)/G(T) subunit beta-1	P62873	GNB1	0
Isoleucine--tRNA ligase, mitochondrial	Q9NSE4	IARS2	Mitochondrion matrix {ECO:0000250}.
Enoyl-CoA hydratase, mitochondrial	P30084	ECHS1	Mitochondrion matrix.
28S ribosomal protein S18b, mitochondrial	Q9Y676	MRPS18B	Mitochondrion {ECO:0000269|PubMed:25838379}.
Protein-cysteine N-palmitoyltransferase HHAT-like protein	Q9HCP6	HHATL	Endoplasmic reticulum membrane {ECO:0000250}; Multi-pass membrane protein {ECO:0000250}.
NADH dehydrogenase [ubiquinone] 1 alpha subcomplex subunit 12	Q9UI09	NDUFA12	Mitochondrion inner membrane {ECO:0000305|PubMed:12611891}; Peripheral membrane protein {ECO:0000255}; Matrix side {ECO:0000305}.
F-actin-capping protein subunit alpha-2	P47755	CAPZA2	0
Coiled-coil-helix-coiled-coil-helix domain-containing protein 2	Q9Y6H1	CHCHD2	Nucleus {ECO:0000269|PubMed:23303788}. Mitochondrion {ECO:0000269|PubMed:25662902}. Mitochondrion intermembrane space {ECO:0000269|PubMed:25662902}. Note=Mainly localised in the intermembrane space. {ECO:0000269|PubMed:25662902}.

Supplemental Table 1 Page 43. All proteins quantitated in study

Protein Name	Uniprot	Gene	Subcellular localization
40S ribosomal protein S4, X isoform	P62701	RPS4X	Cytoplasm {ECO:0000269|PubMed:17289661}. Note=Localized in cytoplasmic mRNP granules containing untranslated mRNAs.
Guanine nucleotide-binding protein G(s) subunit alpha isoforms XLas	Q5JWF2	GNAS	Cell membrane {ECO:0000269|PubMed:27120771}; Peripheral membrane protein {ECO:0000250|UniProtKB:Q63803}. Apical cell membrane {ECO:0000269|PubMed:27120771}.
Alpha-actinin-3	Q08043	ACTN3	0
Importin subunit alpha-4	O00505	KPNA3	Cytoplasm {ECO:0000250}. Nucleus {ECO:0000250}.
Heat shock protein HSP 90-beta	P08238	HSP90AB1	Cytoplasm {ECO:0000269|PubMed:16580629, ECO:0000269|PubMed:18239673, ECO:0000269|PubMed:24880080, ECO:0000269|PubMed:9482106}. Melanosome {ECO:0000269|PubMed:17081065}. Nucleus {ECO:0000269|PubMed:18239673}. Secreted {ECO:0000269|PubMed:20599762}. Cell membrane {ECO:0000269|PubMed:20599762}. Note=Identified by mass spectrometry in melanosome fractions from stage I to stage IV (PubMed:17081065). Translocates with BIRC2 from the nucleus to the cytoplasm during differentiation (PubMed:18239673). Secreted when associated with TGFB1 processed form (LAP) (PubMed:20599762). {ECO:0000269|PubMed:17081065, ECO:0000269|PubMed:18239673, ECO:0000269|PubMed:20599762}.
COP9 signalosome complex subunit 4	Q9BT78	COPS4	Cytoplasm. Nucleus. Cytoplasmic vesicle, secretory vesicle, synaptic vesicle.
ATPase family AAA domain-containing protein 3A	Q9NVI7	ATAD3A	Mitochondrion inner membrane {ECO:0000269|PubMed:18063578, ECO:0000269|PubMed:20154147, ECO:0000269|PubMed:20332122, ECO:0000269|PubMed:20349121}; Single-pass membrane protein {ECO:0000269|PubMed:20154147, ECO:0000269|PubMed:20349121}. Mitochondrion matrix, mitochondrion nucleoid {ECO:0000269|PubMed:17210950, ECO:0000269|PubMed:18063578}. Note=In the mitochondrial inner membrane, enriched in sites with the potential to form contacts with the outer membrane (PubMed:20349121, PubMed:20154147). The N-terminal domain interacts with the inner surface of the mitochondrial outer membrane and the C-terminal domain localizes in a specific matrix compartment, where it is associated with nucleoids (PubMed:18063578). {ECO:0000269|PubMed:18063578, ECO:0000269|PubMed:20154147, ECO:0000269|PubMed:20349121}.
NADH-ubiquinone oxidoreductase 75 kDa subunit, mitochondrial	P28331	NDUFS1	Mitochondrion inner membrane.
Pre-B-cell leukemia transcription factor-interacting protein 1	Q96AQ6	PBXIP1	Cytoplasm, cytoskeleton {ECO:0000269|PubMed:12360403, ECO:0000269|PubMed:17043237}. Nucleus {ECO:0000269|PubMed:10825160, ECO:0000269|PubMed:12360403}. Note=Shuttles between the nucleus and the cytosol (PubMed:12360403). Mainly localized in the cytoplasm, associated with microtubules (PubMed:10825160, PubMed:12360403). Detected in small amounts in the nucleus (PubMed:10825160).
L-xylulose reductase	Q7Z4W1	DCXR	Membrane {ECO:0000250}; Peripheral membrane protein {ECO:0000250}. Note=Probably recruited to membranes via an interaction with phosphatidylinositol. {ECO:0000250}.

Supplemental Table 1 Page 44. All proteins quantitated in study

Protein Name	Uniprot	Gene	Subcellular localization
Malate dehydrogenase, mitochondrial	P40926	MDH2	Mitochondrion matrix {ECO:0000250|UniProtKB:P04636}.
NADH dehydrogenase [ubiquinone] iron-sulfur protein 8, mitochondrial	O00217	NDUFS8	Mitochondrion {ECO:0000305}.
Isochorismatase domain-containing protein 2	Q96AB3	ISOC2	Cytoplasm {ECO:0000269|PubMed:17658461}. Nucleus {ECO:0000269|PubMed:17658461}. Note=Localizes to the nucleus in the presence of CDKN2A.
Hydroxymethylglutaryl-CoA lyase, mitochondrial	P35914	HMGCL	Mitochondrion matrix. Peroxisome. Note=Unprocessed form is peroxisomal.
NADH-ubiquinone oxidoreductase chain 2	P03891	MT-ND2	Mitochondrion inner membrane; Multi-pass membrane protein.
Prolactin regulatory element-binding protein	Q9HCU5	PREB	Endoplasmic reticulum membrane {ECO:0000250|UniProtKB:Q9WTV0}; Single-pass membrane protein {ECO:0000250|UniProtKB:Q9WTV0}. Nucleus {ECO:0000250|UniProtKB:Q9WTV0}. Note=Concentrates at endoplasmic reticulum exit sites (ERES), also known as transitional endoplasmic reticulum (tER). {ECO:0000269|PubMed:25202031, ECO:0000269|PubMed:28442536}.
Myoglobin	P02144	MB	0
Calcium uptake protein 1, mitochondrial	Q9BPX6	MICU1	Mitochondrion inner membrane {ECO:0000269|PubMed:20693986, ECO:0000269|PubMed:24332854, ECO:0000269|PubMed:26387864, ECO:0000269|PubMed:26774479, ECO:0000269|PubMed:27099988, ECO:0000305|PubMed:24231807}; Single-pass membrane protein {ECO:0000255}. Mitochondrion intermembrane space {ECO:0000269|PubMed:23747253, ECO:0000269|PubMed:24560927, ECO:0000269|PubMed:26387864}.
Electron transfer flavoprotein-ubiquinone oxidoreductase, mitochondrial	Q16134	ETFDH	Mitochondrion inner membrane.
Vinculin	P18206	VCL	Cell membrane {ECO:0000250|UniProtKB:P12003}; Peripheral membrane protein {ECO:0000250|UniProtKB:P12003}; Cytoplasmic side {ECO:0000250|UniProtKB:P12003}. Cell junction, adherens junction {ECO:0000250|UniProtKB:P12003}. Cell junction, focal adhesion {ECO:0000250|UniProtKB:P12003}. Cytoplasm, cytoskeleton {ECO:0000250|UniProtKB:P85972}. Cell membrane, sarcolemma {ECO:0000250|UniProtKB:Q64727}; Peripheral membrane protein {ECO:0000250|UniProtKB:Q64727}; Cytoplasmic side {ECO:0000250|UniProtKB:Q64727}. Note=Recruitment to cell-cell junctions occurs in a myosin II-dependent manner. Interaction with CTNNB1 is necessary for its localization to the cell-cell junctions. {ECO:0000250|UniProtKB:P12003}.

Supplemental Table 1 Page 45. All proteins quantitated in study

Protein Name	Uniprot	Gene	Subcellular localization
Mitochondrial antiviral-signaling protein	Q7Z434	MAVS	Mitochondrion outer membrane {ECO:0000269|PubMed:16125763}. Mitochondrion {ECO:0000269|PubMed:11780052, ECO:0000269|PubMed:17709747}. Peroxisome {ECO:0000269|PubMed:20451243}.
Glucosidase 2 subunit beta	P14314	PRKCSH	Endoplasmic reticulum {ECO:0000255|PROSITE-ProRule:PRU10138, ECO:0000305|PubMed:10929008}.
Keratin, type I cytoskeletal 9	P35527	KRT9	0
Myosin-7B	A7E2Y1	MYH7B	Membrane {ECO:0000269|PubMed:15755502}; Peripheral membrane protein {ECO:0000269|PubMed:15755502}.
Alanine aminotransferase 2	Q8TD30	GPT2	0
Isocitrate dehydrogenase [NAD] subunit gamma, mitochondrial	P51553	IDH3G	Mitochondrion {ECO:0000269|PubMed:11256614}.
Enoyl-CoA delta isomerase 2, mitochondrial	O75521	ECI2	Isoform 1: Mitochondrion {ECO:0000250}.; Isoform 2: Peroxisome matrix.
Enoyl-[acyl-carrier-protein] reductase, mitochondrial	Q9BV79	MECR	Isoform 1: Mitochondrion {ECO:0000269|PubMed:12654921}.; Isoform 2: Cytoplasm {ECO:0000269|PubMed:25031892}. Nucleus {ECO:0000269|PubMed:25031892}.
Ankyrin repeat domain-containing protein 2	Q9GZV1	ANKRD2	Cytoplasm, myofibril, sarcomere, I band {ECO:0000250}. Cytoplasm, cytosol. Nucleus. Nucleus, PML body. Note=In the sarcoplasm of differentiated striated muscle cells, where it is cytosolic and enriched in the I band. In nucleus and PML bodies of proliferating and undifferentiated myoblasts. Associates with the euchromatin in the nucleus of myocytes upon muscle stress.
Cytochrome c oxidase subunit NDUFA4	O00483	NDUFA4	Mitochondrion inner membrane {ECO:0000269|PubMed:23746447}; Peripheral membrane protein {ECO:0000269|PubMed:23746447}; Matrix side {ECO:0000269|PubMed:23746447}.
Trimeric intracellular cation channel type A	Q9H6F2	TMEM38A	Sarcoplasmic reticulum membrane {ECO:0000250|UniProtKB:A5A6S6}; Multi-pass membrane protein {ECO:0000250|UniProtKB:A5A6S6}. Nucleus membrane {ECO:0000250|UniProtKB:A5A6S6}.
Voltage-dependent L-type calcium channel subunit alpha-1S	Q13698	CACNA1S	Cell membrane, sarcolemma, T-tubule {ECO:0000250|UniProtKB:P07293}; Multi-pass membrane protein {ECO:0000250|UniProtKB:P07293}.
F-actin-capping protein subunit beta	P47756	CAPZB	Cytoplasm, cytoskeleton {ECO:0000250}. Cytoplasm, myofibril, sarcomere {ECO:0000250}.
Myozenin-3	Q8TDC0	MYOZ3	Cytoplasm, myofibril, sarcomere, Z line {ECO:0000250}. Note=Localized at the Z-line of skeletal muscle. {ECO:0000250}.
Cofilin-2	Q9Y281	CFL2	Nucleus matrix {ECO:0000250}. Cytoplasm, cytoskeleton {ECO:0000250}. Note=Colocalizes with CSPR3 in the Z line of sarcomeres. {ECO:0000269|PubMed:19752190}.
ATP-binding cassette sub-family B member 10, mitochondrial	Q9NRK6	ABCB10	Mitochondrion inner membrane; Multi-pass membrane protein.
Dolichyl-diphosphooligosaccharide--protein glycosyltransferase subunit 2	P04844	RPN2	Endoplasmic reticulum {ECO:0000250|UniProtKB:F1PCT7}. Endoplasmic reticulum membrane; Multi-pass membrane protein {ECO:0000305}.

Supplemental Table 1 Page 46. All proteins quantitated in study

Protein Name	Uniprot	Gene	Subcellular localization
Voltage-dependent calcium channel gamma-1 subunit	Q06432	CACNG1	Cell membrane, sarcolemma {ECO:0000250|UniProtKB:P19518}; Multi-pass membrane protein {ECO:0000250|UniProtKB:P19518}.
Elongation factor 2	P13639	EEF2	Cytoplasm {ECO:0000269|PubMed:25064856}. Nucleus {ECO:0000269|PubMed:24648518}. Note=Phosphorylation by CSK promotes cleavage and SUMOylation-dependent nuclear translocation of the C-terminal cleavage product. {ECO:0000269|PubMed:24648518}.
Lipoamide acyltransferase component of branched-chain alpha-keto acid dehydrogenase complex, mitochondrial	P11182	DBT	Mitochondrion matrix.
Annexin A6	P08133	ANXA6	Cytoplasm {ECO:0000250}. Melanosome {ECO:0000269|PubMed:17081065}. Note=Identified by mass spectrometry in melanosome fractions from stage I to stage IV.
Fat storage-inducing transmembrane protein 1	A5D6W6	FITM1	Endoplasmic reticulum membrane {ECO:0000269|PubMed:18160536}; Multi-pass membrane protein {ECO:0000269|PubMed:18160536}.
Sepiapterin reductase	P35270	SPR	Cytoplasm.
Phosphatidylethanolamine-binding protein 1	P30086	PEBP1	Cytoplasm {ECO:0000250}.
ATPase family AAA domain-containing protein 1	Q8NBU5	ATAD1	Peroxisome {ECO:0000250|UniProtKB:Q9D5T0}. Cell junction, synapse, postsynaptic cell membrane {ECO:0000250|UniProtKB:Q9D5T0}.
Protein NipSnap homolog 3B	Q9BS92	NIPSNAP3B	0
Prenylcysteine oxidase 1	Q9UHG3	PCYOX1	Lysosome.
Cytochrome c oxidase subunit 6A2, mitochondrial	Q02221	COX6A2	Mitochondrion inner membrane.
39S ribosomal protein L37, mitochondrial	Q9BZE1	MRPL37	Mitochondrion {ECO:0000269|PubMed:25278503, ECO:0000269|PubMed:25838379, ECO:0000269|PubMed:28892042}.
Histone H3.3	P84243	H3F3A; H3F3B	Nucleus. Chromosome.
40S ribosomal protein S2	P15880	RPS2	0
D-2-hydroxyglutarate dehydrogenase, mitochondrial	Q8N465	D2HGDH	Mitochondrion {ECO:0000305|PubMed:15070399}.
Histone H1.2	P16403	HIST1H1C	Nucleus. Chromosome. Note=Mainly localizes in euchromatin. Distribution goes in parallel with DNA concentration.
Sarcoplasmic/endoplasmic reticulum calcium ATPase 2	P16615	ATP2A2	Endoplasmic reticulum membrane {ECO:0000250|UniProtKB:O55143}; Multi-pass membrane protein {ECO:0000255}. Sarcoplasmic reticulum membrane {ECO:0000250|UniProtKB:O55143}; Multi-pass membrane protein {ECO:0000255}.
Glutaryl-CoA dehydrogenase, mitochondrial	Q92947	GCDH	Mitochondrion matrix.

Supplemental Table 1 Page 47. All proteins quantitated in study

Protein Name	Uniprot	Gene	Subcellular localization
Cytochrome b-c1 complex subunit 1, mitochondrial	P31930	UQCRC1	Mitochondrion inner membrane.
AFG3-like protein 2	Q9Y4W6	AFG3L2	Mitochondrion {ECO:0000269|PubMed:10395799}. Mitochondrion inner membrane {ECO:0000250|UniProtKB:Q8JZQ2}; Multi-pass membrane protein {ECO:0000255}.
Voltage-dependent anion-selective channel protein 2	P45880	VDAC2	Mitochondrion outer membrane {ECO:0000269|PubMed:7539795}.
Cytochrome c oxidase subunit 1	P00395	MT-CO1	Mitochondrion inner membrane; Multi-pass membrane protein.
Elongation factor Ts, mitochondrial	P43897	TSFM	Mitochondrion.
Saccharopine dehydrogenase-like oxidoreductase	Q8NBX0	SCCPDH	0
Amine oxidase [flavin-containing] B	P27338	MAOB	Mitochondrion outer membrane; Single-pass type IV membrane protein; Cytoplasmic side.
40S ribosomal protein S3	P23396	RPS3	Cytoplasm {ECO:0000269|PubMed:16314389, ECO:0000269|PubMed:17289661, ECO:0000269|PubMed:17560175, ECO:0000269|PubMed:18045535, ECO:0000269|PubMed:20217897, ECO:0000269|PubMed:21871177}. Nucleus {ECO:0000269|PubMed:17560175, ECO:0000269|PubMed:18045535, ECO:0000269|PubMed:19460357, ECO:0000269|PubMed:20217897, ECO:0000269|PubMed:21871177}. Nucleus, nucleolus {ECO:0000269|PubMed:16314389, ECO:0000269|PubMed:19460357}. Mitochondrion inner membrane {ECO:0000269|PubMed:23911537}; Peripheral membrane protein {ECO:0000269|PubMed:23911537}. Cytoplasm, cytoskeleton, spindle {ECO:0000269|PubMed:23131551}. Note=In normal cells, located mainly in the cytoplasm with small amounts in the nucleus but translocates to the nucleus in cells undergoing apoptosis (By similarity). Nuclear translocation is induced by DNA damaging agents such as hydrogen peroxide (PubMed:17560175). Accumulates in the mitochondrion in response to increased ROS levels (PubMed:23911537). Localizes to the spindle during mitosis (PubMed:23131551). Localized in cytoplasmic mRNP granules containing untranslated mRNAs (PubMed:17289661). {ECO:0000250|UniProtKB:P62908, ECO:0000269|PubMed:17289661, ECO:0000269|PubMed:17560175, ECO:0000269|PubMed:23131551, ECO:0000269|PubMed:23911537}.
Protein Niban	Q9BZQ8	FAM129A	Cytoplasm {ECO:0000269|PubMed:16949643}. Membrane {ECO:0000305}; Lipid-anchor {ECO:0000305}.
ATP synthase subunit g, mitochondrial	O75964	ATP5MG	Mitochondrion. Mitochondrion inner membrane.
Adenylate kinase isoenzyme 1	P00568	AK1	Cytoplasm.
Acyl-coenzyme A thioesterase 9, mitochondrial	Q9Y305	ACOT9	Mitochondrion {ECO:0000250}.

Supplemental Table 1 Page 48. All proteins quantitated in study

Protein Name	Uniprot	Gene	Subcellular localization
Haloacid dehalogenase-like hydrolase domain-containing 5	Q9BXW7	HDHD5	0
Integrin beta-1	P05556	ITGB1	Cell membrane {ECO:0000303|PubMed:10455171}; Single-pass type I membrane protein {ECO:0000255}. Cell projection, invadopodium membrane {ECO:0000269|PubMed:10455171}; Single-pass type I membrane protein {ECO:0000255}. Cell projection, ruffle membrane {ECO:0000303|PubMed:10455171}; Single-pass type I membrane protein {ECO:0000255}. Recycling endosome {ECO:0000269|PubMed:16256741}. Melanosome {ECO:0000269|PubMed:17081065}. Cleavage furrow {ECO:0000269|PubMed:17956333}. Cell projection, lamellipodium {ECO:0000269|PubMed:11919189}. Cell projection, ruffle {ECO:0000269|PubMed:11919189}. Cell junction, focal adhesion {ECO:0000269|PubMed:17158881}. Cell surface {ECO:0000269|PubMed:17158881}. Note=Isoform 2 does not localize to focal adhesions. Highly enriched in stage I melanosomes. Located on plasma membrane of neuroblastoma NMB7 cells. In a lung cancer cell line, in prometaphase and metaphase, localizes diffusely at the membrane and in a few intracellular vesicles. In early telophase, detected mainly on the matrix-facing side of the cells. By mid-telophase, concentrated to the ingressing cleavage furrow, mainly to the basal side of the furrow. In late telophase, concentrated to the extending protrusions formed at the opposite ends of the spreading daughter cells, in vesicles at the base of the lamellipodia formed by the separating daughter cells. Colocalizes with ITGB1BP1 and metastatic suppressor protein NME2 at the edge or peripheral ruffles and lamellipodia during the early stages of cell spreading on fibronectin or collagen. Translocates from peripheral focal adhesions sites to fibrillar adhesions in a ITGB1BP1-dependent manner. Enriched preferentially at invadopodia, cell membrane protrusions that correspond to sites of cell invasion, in a collagen-dependent manner. Localized at plasma and ruffle membranes in a collagen-independent manner. {ECO:0000269|PubMed:10455171, ECO:0000303|PubMed:10455171}.; Isoform 5: Cell membrane, sarcolemma {ECO:0000250}. Cell junction {ECO:0000250}. Note=In cardiac muscle, isoform 5 is found in costameres and intercalated disks. {ECO:0000250}.
Protein SCO2 homolog, mitochondrial	O43819	SCO2	Mitochondrion inner membrane {ECO:0000269|PubMed:15229189}; Single-pass membrane protein {ECO:0000255}.
Heat shock protein 75 kDa, mitochondrial	Q12931	TRAP1	Mitochondrion {ECO:0000269|PubMed:23564345}. Mitochondrion inner membrane {ECO:0000269|PubMed:23564345}. Mitochondrion matrix {ECO:0000269|PubMed:23564345}.
[Pyruvate dehydrogenase (acetyl-transferring)] kinase isozyme 2, mitochondrial	Q15119	PDK2	Mitochondrion matrix.
Citrate synthase, mitochondrial	O75390	CS	Mitochondrion matrix.
D-glutamate cyclase, mitochondrial	Q7Z3D6	DGLUCY	Mitochondrion matrix {ECO:0000250|UniProtKB:Q8BH86}.

Supplemental Table 1 Page 49. All proteins quantitated in study

Protein Name	Uniprot	Gene	Subcellular localization
Talin-2	Q9Y4G6	TLN2	Cytoplasm {ECO:0000269|PubMed:27223613}. Cell junction, focal adhesion {ECO:0000269|PubMed:12422219}. Cell junction, synapse {ECO:0000269|PubMed:12422219}. Cell membrane; Peripheral membrane protein; Cytoplasmic side {ECO:0000269|PubMed:12422219}. Cytoplasm, cytoskeleton {ECO:0000269|PubMed:12422219}. Note=Focal adhesion plaques and synapses (PubMed:12422219). {ECO:0000269|PubMed:12422219}.
Caveolae-associated protein 4	Q5BKX8	CAVIN4	Cytoplasm, myofibril, sarcomere {ECO:0000250|UniProtKB:A2AMM0}. Cytoplasm {ECO:0000250|UniProtKB:A2AMM0}. Cytoplasm, cytosol {ECO:0000250|UniProtKB:A2AMM0}. Cell membrane, sarcolemma {ECO:0000250|UniProtKB:A2AMM0}. Membrane, caveola {ECO:0000269|PubMed:19525939}. Cell membrane {ECO:0000269|PubMed:26497963}. Note=In cardiomyocytes, accumulates in the Z-line of the sarcomere. In vascular smooth muscle cells, detected diffusely throughout the cytoplasm. Localizes in the caveolae in a caveolin-dependent manner. {ECO:0000250|UniProtKB:A2AMM0}.
Coiled-coil domain-containing protein 127	Q96BQ5	CCDC127	0
Serine beta-lactamase-like protein LACTB, mitochondrial	P83111	LACTB	Mitochondrion {ECO:0000269|PubMed:11551941, ECO:0000269|PubMed:19858488, ECO:0000269|PubMed:28329758}.
Caveolae-associated protein 1	Q6NZI2	CAVIN1	Membrane, caveola {ECO:0000269|PubMed:15242332, ECO:0000269|PubMed:18056712, ECO:0000269|PubMed:19525939}. Cell membrane {ECO:0000269|PubMed:15242332, ECO:0000269|PubMed:17026959, ECO:0000269|PubMed:18056712}. Microsome {ECO:0000269|PubMed:15242332, ECO:0000269|PubMed:17026959}. Endoplasmic reticulum {ECO:0000250|UniProtKB:P85125}. Cytoplasm, cytosol {ECO:0000269|PubMed:15242332}. Mitochondrion {ECO:0000269|PubMed:15242332, ECO:0000269|PubMed:17026959}. Nucleus {ECO:0000269|PubMed:15242332, ECO:0000269|PubMed:17026959}. Note=Translocates to the cytoplasm from the caveolae upon insulin stimulation (PubMed:17026959). Colocalizes with CAV1 in lipid rafts in adipocytes. Localizes in the caveolae in a caveolin-dependent manner (By similarity). {ECO:0000250|UniProtKB:O54724, ECO:0000269|PubMed:17026959}.
Protein cordon-bleu	O75128	COBL	Cell membrane {ECO:0000250}; Peripheral membrane protein {ECO:0000250}; Cytoplasmic side {ECO:0000250}. Cytoplasm, cytoskeleton {ECO:0000250}. Cell projection, ruffle {ECO:0000250}. Cytoplasm {ECO:0000250}. Note=Recruited to the cell membrane via interaction with PACSIN1. Colocalizes with the actin cytoskeleton. Detected throughout the neuron cell body, as well as in axons and dendrites (By similarity). {ECO:0000250}.
Peroxiredoxin-1	Q06830	PRDX1	Cytoplasm {ECO:0000269|PubMed:17081065, ECO:0000269|PubMed:9497357}. Melanosome {ECO:0000269|PubMed:17081065}. Note=Identified by mass spectrometry in melanosome fractions from stage I to stage IV.

Supplemental Table 1 Page 50. All proteins quantitated in study

Protein Name	Uniprot	Gene	Subcellular localization
Dystrophin	P11532	DMD	Cell membrane, sarcolemma {ECO:0000250|UniProtKB:P11531}; Peripheral membrane protein {ECO:0000250|UniProtKB:P11531}; Cytoplasmic side {ECO:0000250|UniProtKB:P11531}. Cytoplasm, cytoskeleton {ECO:0000250|UniProtKB:P11531}. Cell junction, synapse, postsynaptic cell membrane {ECO:0000250|UniProtKB:P11531}. Note=In muscle cells, sarcolemma localization requires the presence of ANK2, while localization to costameres requires the presence of ANK3. Localizes to neuromuscular junctions (NMJs). In adult muscle, NMJ localization depends upon ANK2 presence, but not in newborn animals. {ECO:0000250|UniProtKB:P11531}.
39S ribosomal protein L28, mitochondrial	Q13084	MRPL28	Mitochondrion {ECO:0000269|PubMed:25278503, ECO:0000269|PubMed:28892042}.
Cytochrome c oxidase subunit 4 isoform 1, mitochondrial	P13073	COX4I1	Mitochondrion inner membrane {ECO:0000250|UniProtKB:P19783}.
Ankyrin-1	P16157	ANK1	Isoform Er1: Cytoplasm, cytoskeleton. Note=Probably the other erythrocyte (Er) isoforms, are located near the surface of erythrocytic plasma membrane.; Isoform Mu17: Membrane. Cytoplasm, myofibril, sarcomere, M line. Note=Colocalizes with OBSCN isoform 3/obscurin at the M line in differentiated skeletal muscle cells.; Isoform Mu18: Sarcoplasmic reticulum {ECO:0000305}.; Isoform Mu19: Sarcoplasmic reticulum {ECO:0000305}.; Isoform Mu20: Sarcoplasmic reticulum {ECO:0000305}.
Voltage-dependent anion-selective channel protein 3	Q9Y277	VDAC3	Mitochondrion outer membrane.
HIG1 domain family member 2A, mitochondrial	Q9BW72	HIGD2A	Mitochondrion membrane {ECO:0000255|PROSITE-ProRule:PRU00836, ECO:0000269|PubMed:22342701}; Multi-pass membrane protein {ECO:0000255|PROSITE-ProRule:PRU00836, ECO:0000269|PubMed:22342701}. Mitochondrion inner membrane {ECO:0000305|PubMed:22342701}.
Talin-1	Q9Y490	TLN1	Cell projection, ruffle membrane {ECO:0000250}; Peripheral membrane protein {ECO:0000250}; Cytoplasmic side {ECO:0000250}. Cytoplasm, cytoskeleton {ECO:0000250}. Cell surface {ECO:0000250|UniProtKB:P26039}. Cell junction, focal adhesion {ECO:0000250|UniProtKB:P26039}. Note=Colocalizes with LAYN at the membrane ruffles. Localized preferentially in focal adhesions than fibrillar adhesions (By similarity). {ECO:0000250}.
Aconitate hydratase, mitochondrial	Q99798	ACO2	Mitochondrion {ECO:0000250|UniProtKB:P16276}.
Endoplasmin	P14625	HSP90B1	Endoplasmic reticulum lumen {ECO:0000305|PubMed:12475965}. Sarcoplasmic reticulum lumen {ECO:0000250|UniProtKB:P41148}. Melanosome {ECO:0000269|PubMed:12643545, ECO:0000269|PubMed:17081065}. Note=Identified by mass spectrometry in melanosome fractions from stage I to stage IV. {ECO:0000269|PubMed:12643545, ECO:0000269|PubMed:17081065}.

Supplemental Table 1 Page 51. All proteins quantitated in study

Protein Name	Uniprot	Gene	Subcellular localization
Prostaglandin E synthase 2	Q9H7Z7	PTGES2	Golgi apparatus membrane {ECO:0000269|PubMed:12835322}; Single-pass membrane protein {ECO:0000255}.; Prostaglandin E synthase 2 truncated form: Cytoplasm, perinuclear region {ECO:0000269|PubMed:12835322}. Note=Synthesized as a Golgi membrane-bound protein, which is further cleaved into the predominant soluble truncated form. The truncated form is cytoplasmic and is enriched in the perinuclear region. {ECO:0000269|PubMed:12835322}.
Sarcolemmal membrane-associated protein	Q14BN4	SLMAP	Cell membrane, sarcolemma {ECO:0000250}; Single-pass type IV membrane protein {ECO:0000250}. Cytoplasm, cytoskeleton, microtubule organizing center, centrosome {ECO:0000250}. Note=Membrane-associated. Distributed in the transverse tubules and near the junctional sarcoplasmic reticulum. Detected along the Z- and M-lines in cardiomyocytes. Centrosome. Localizes to the centrosomes in a microtubule-dependent manner (By similarity). {ECO:0000250}.
Paraplegin	Q9UQ90	SPG7	Mitochondrion inner membrane {ECO:0000269|PubMed:9635427}; Multi-pass membrane protein {ECO:0000255}.
Myosin-2	Q9UKX2	MYH2	Cytoplasm, myofibril. Note=Thick filaments of the myofibrils.
Isocitrate dehydrogenase [NAD] subunit alpha, mitochondrial	P50213	IDH3A	Mitochondrion.
Calreticulin	P27797	CALR	Endoplasmic reticulum lumen {ECO:0000269|PubMed:10358038, ECO:0000269|PubMed:11149926}. Cytoplasm, cytosol {ECO:0000269|PubMed:11149926}. Secreted, extracellular space, extracellular matrix {ECO:0000305}. Cell surface {ECO:0000269|PubMed:10358038}. Sarcoplasmic reticulum lumen {ECO:0000250|UniProtKB:P28491}. Note=Also found in cell surface (T cells), cytosol and extracellular matrix (PubMed:10358038). Associated with the lytic granules in the cytolytic T-lymphocytes. {ECO:0000269|PubMed:10358038, ECO:0000269|PubMed:8418194}.
MICOS complex subunit MIC27	Q6UXV4	APOOL	Mitochondrion inner membrane {ECO:0000269|PubMed:23704930, ECO:0000269|PubMed:25764979}; Multi-pass membrane protein {ECO:0000269|PubMed:23704930}. Mitochondrion {ECO:0000269|PubMed:25781180, ECO:0000269|PubMed:25997101}.
Monocarboxylate transporter 1	P53985	SLC16A1	Cell membrane {ECO:0000269|PubMed:15505343, ECO:0000269|PubMed:17127621, ECO:0000269|PubMed:24390345}; Multi-pass membrane protein {ECO:0000269|PubMed:15505343, ECO:0000269|PubMed:17127621, ECO:0000269|PubMed:24390345}.
Ras-related protein Rab-11B	Q15907	RAB11B	Recycling endosome membrane {ECO:0000250}; Lipid-anchor {ECO:0000250}; Cytoplasmic side {ECO:0000250}. Cytoplasmic vesicle, secretory vesicle, synaptic vesicle membrane {ECO:0000250}; Lipid-anchor {ECO:0000250}; Cytoplasmic side {ECO:0000250}. Cytoplasmic vesicle, phagosome membrane {ECO:0000305|PubMed:21255211}; Lipid-anchor {ECO:0000305|PubMed:21255211}; Cytoplasmic side {ECO:0000305|PubMed:21255211}. Note=Recruited to phagosomes containing S.aureus.

Supplemental Table 1 Page 52. All proteins quantitated in study

Protein Name	Uniprot	Gene	Subcellular localization
Presequence protease, mitochondrial	Q5JRX3	PITRM1	Mitochondrion matrix {ECO:0000269|PubMed:16849325, ECO:0000269|PubMed:19196155}.
60S acidic ribosomal protein P0	P05388	RPLP0	Nucleus {ECO:0000269|PubMed:19188445}. Cytoplasm {ECO:0000269|PubMed:19188445}. Note=Localized in cytoplasmic mRNP granules containing untranslated mRNAs (PubMed:19188445, PubMed:17289661). {ECO:0000269|PubMed:17289661, ECO:0000269|PubMed:19188445}.
Succinate--CoA ligase [ADP/GDP-forming] subunit alpha, mitochondrial	P53597	SUCLG1	Mitochondrion {ECO:0000255|HAMAP-Rule:MF_03222}.
Myosin light chain 1/3, skeletal muscle isoform	P05976	MYL1	0
Nebulin	P20929	NEB	Cytoplasm, myofibril, sarcomere. Cytoplasm, cytoskeleton.
Calcium/calmodulin-dependent protein kinase type II subunit alpha	Q9UQM7	CAMK2A	Cell junction, synapse {ECO:0000250|UniProtKB:P11275}. Cell junction, synapse, postsynaptic cell membrane, postsynaptic density {ECO:0000250|UniProtKB:P11275}. Cell projection, dendritic spine {ECO:0000269|PubMed:28130356}. Cell projection, dendrite {ECO:0000269|PubMed:28130356}. Note=Postsynaptic lipid rafts. {ECO:0000250|UniProtKB:P11275}.
28S ribosomal protein S9, mitochondrial	P82933	MRPS9	Mitochondrion {ECO:0000269|PubMed:25838379}.
Protein SCO1 homolog, mitochondrial	O75880	SCO1	Mitochondrion {ECO:0000269|PubMed:9878253}. Mitochondrion inner membrane {ECO:0000269|PubMed:15229189}; Single-pass membrane protein {ECO:0000255}.
Alpha-sarcoglycan	Q16586	SGCA	Cell membrane, sarcolemma {ECO:0000250}; Single-pass type I membrane protein {ECO:0000250}. Cytoplasm, cytoskeleton {ECO:0000250}.
NADH dehydrogenase [ubiquinone] iron-sulfur protein 6, mitochondrial	O75380	NDUFS6	Mitochondrion inner membrane {ECO:0000305|PubMed:12611891}; Peripheral membrane protein {ECO:0000305}; Matrix side {ECO:0000305}.
Troponin C, slow skeletal and cardiac muscles	P63316	TNNC1	0
2-oxoglutarate dehydrogenase, mitochondrial	Q02218	OGDH	Mitochondrion matrix {ECO:0000305|PubMed:29211711}. Nucleus {ECO:0000269|PubMed:29211711}. Note=Mainly localizes in the mitochondrion. A small fraction localizes to the nucleus, where the 2-oxoglutarate dehydrogenase complex is required for histone succinylation. {ECO:0000269|PubMed:29211711}.
Agmatinase, mitochondrial	Q9BSE5	AGMAT	Mitochondrion.
Dynamin-like 120 kDa protein, mitochondrial	O60313	OPA1	Mitochondrion inner membrane {ECO:0000269|PubMed:11017079, ECO:0000269|PubMed:16778770, ECO:0000269|PubMed:20974897, ECO:0000269|PubMed:28746876}; Single-pass membrane protein {ECO:0000255}. Mitochondrion intermembrane space {ECO:0000250|UniProtKB:P58281}. Mitochondrion membrane {ECO:0000269|PubMed:24616225}. Note=Detected at contact sites between endoplamic reticulum and mitochondrion membranes. {ECO:0000269|PubMed:24616225}.

Supplemental Table 1 Page 53. All proteins quantitated in study

Protein Name	Uniprot	Gene	Subcellular localization
Acyl-coenzyme A thioesterase 1	Q86TX2	ACOT1	Cytoplasm, cytosol {ECO:0000269|PubMed:16940157}.
Succinyl-CoA:3-ketoacid coenzyme A transferase 1, mitochondrial	P55809	OXCT1	Mitochondrion matrix.
Protein-arginine deiminase type-2	Q9Y2J8	PADI2	Cytoplasm {ECO:0000269|PubMed:12392711}.
Plasma membrane calcium-transporting ATPase 2	Q01814	ATP2B2	Cell junction, synapse {ECO:0000250|UniProtKB:Q9R0K7}. Cell membrane {ECO:0000255}; Multi-pass membrane protein {ECO:0000255}.
SRA stem-loop-interacting RNA-binding protein, mitochondrial	Q9GZT3	SLIRP	Mitochondrion {ECO:0000269|PubMed:16762838}. Nucleus {ECO:0000269|PubMed:16762838}. Note=Predominantly mitochondrial. Some fraction is nuclear. In the nucleus, it is recruited to nuclear receptor target promoters.
Retinol dehydrogenase 13	Q8NBN7	RDH13	Mitochondrion inner membrane {ECO:0000269|PubMed:18039331}; Peripheral membrane protein {ECO:0000269|PubMed:18039331}. Note=Localized on the outer side of the inner mitochondrial membrane. {ECO:0000269|PubMed:18039331}.
Endonuclease domain-containing 1 protein	O94919	ENDOD1	Secreted {ECO:0000305}.
NADH dehydrogenase [ubiquinone] 1 beta subcomplex subunit 4	O95168	NDUFB4	Mitochondrion inner membrane {ECO:0000305|PubMed:12611891}; Single-pass membrane protein {ECO:0000255}; Matrix side {ECO:0000305}.
Mitochondrial import inner membrane translocase subunit Tim21	Q9BVV7	TIMM21	Mitochondrion membrane {ECO:0000305}; Single-pass membrane protein {ECO:0000305}.
Xin actin-binding repeat-containing protein 1	Q702N8	XIRP1	Cell junction {ECO:0000269|PubMed:15454575}. Note=Colocalizes with actin stress fibers.
NADH dehydrogenase [ubiquinone] iron-sulfur protein 2, mitochondrial	O75306	NDUFS2	Mitochondrion inner membrane {ECO:0000269|PubMed:12611891, ECO:0000269|PubMed:9585441}; Peripheral membrane protein {ECO:0000269|PubMed:12611891}; Matrix side {ECO:0000269|PubMed:12611891}.
Succinate dehydrogenase cytochrome b560 subunit, mitochondrial	Q99643	SDHC	Mitochondrion inner membrane; Multi-pass membrane protein.
Mitochondrial-processing peptidase subunit alpha	Q10713	PMPCA	Mitochondrion matrix {ECO:0000250|UniProtKB:P20069}. Mitochondrion inner membrane {ECO:0000269|PubMed:25808372}.
Serine protease HTRA2, mitochondrial	O43464	HTRA2	Mitochondrion intermembrane space. Mitochondrion membrane {ECO:0000305}; Single-pass membrane protein {ECO:0000305}. Note=Predominantly present in the intermembrane space. Released into the cytosol following apoptotic stimuli, such as UV treatment, and stimulation of mitochondria with caspase-8 truncated BID/tBID.
Gamma-sarcoglycan	Q13326	SGCG	Cell membrane, sarcolemma {ECO:0000250}; Single-pass type II membrane protein {ECO:0000250}. Cytoplasm, cytoskeleton {ECO:0000250}.

Supplemental Table 1 Page 54. All proteins quantitated in study

Protein Name	Uniprot	Gene	Subcellular localization
Metaxin-2	O75431	MTX2	Mitochondrion outer membrane {ECO:0000269|PubMed:10381257}. Mitochondrion {ECO:0000269|PubMed:25997101}.
NADH dehydrogenase [ubiquinone] 1 beta subcomplex subunit 7	P17568	NDUFB7	Mitochondrion inner membrane {ECO:0000269|PubMed:21310150}; Peripheral membrane protein {ECO:0000269|PubMed:21310150}. Mitochondrion intermembrane space {ECO:0000269|PubMed:21310150}.
Obscurin	Q5VST9	OBSCN	Isoform 3: Cytoplasm, myofibril, sarcomere, M line {ECO:0000269|PubMed:12527750}. Cytoplasm, myofibril, sarcomere, Z line {ECO:0000269|PubMed:12527750}. Note=In differentiating skeletal muscle cells, isoform 3 primarily localizes to the sarcomeric M-line and less frequently to the Z-disk (PubMed:12527750). Isoform 3 colocalizes with ANK1 isoform Mu17/ank1.5 at the M-line in differentiated skeletal muscle cells (PubMed:12527750).; Cytoplasm, myofibril, sarcomere, M line {ECO:0000250|UniProtKB:A2AAJ9}. Cytoplasm, myofibril, sarcomere, Z line {ECO:0000250|UniProtKB:A2AAJ9}. Cell membrane, sarcolemma {ECO:0000250|UniProtKB:A2AAJ9}. Nucleus {ECO:0000250|UniProtKB:A2AAJ9}. Note=Colocalizes with CDH2 and ATP1B1 to the sarcolemma and to intercalating disks in cardiac muscles. Colocalizes with ATP1B1 to M line and Z line in cardiac muscles. {ECO:0000250|UniProtKB:A2AAJ9}.
NADH-ubiquinone oxidoreductase chain 5	P03915	MT-ND5	Mitochondrion inner membrane {ECO:0000250}; Multi-pass membrane protein {ECO:0000250}.
Very-long-chain (3R)-3-hydroxyacyl-CoA dehydratase 1	B0YJ81	HACD1	Isoform 1: Endoplasmic reticulum membrane {ECO:0000269|PubMed:18554506}; Multi-pass membrane protein {ECO:0000269|PubMed:18554506}.
Transmembrane protein 126A	Q9H061	TMEM126A	Mitochondrion inner membrane {ECO:0000269|PubMed:19327736, ECO:0000269|PubMed:23500070}; Multi-pass membrane protein {ECO:0000269|PubMed:19327736, ECO:0000269|PubMed:23500070}.
Cullin-associated NEDD8-dissociated protein 1	Q86VP6	CAND1	Cytoplasm {ECO:0000269|PubMed:21249194}. Nucleus {ECO:0000269|PubMed:21249194}. Note=Predominantly cytoplasmic.
Hexokinase-1	P19367	HK1	Mitochondrion outer membrane. Note=Its hydrophobic N-terminal sequence may be involved in membrane binding.
60S ribosomal protein L6	Q02878	RPL6	Cytoplasm, cytosol {ECO:0000269|PubMed:25957688}. Cytoplasm {ECO:0000305|PubMed:23636399, ECO:0000305|PubMed:25901680}. Rough endoplasmic reticulum {ECO:0000250|UniProtKB:Q2YGT9}. Note=Detected on cytosolic polysomes (PubMed:25957688). Detected in ribosomes that are associated with the rough endoplasmic reticulum (By similarity). {ECO:0000250|UniProtKB:Q2YGT9, ECO:0000269|PubMed:25957688}.
ER membrane protein complex subunit 2	Q15006	EMC2	Nucleus {ECO:0000250}. Cytoplasm {ECO:0000269|PubMed:22119785}.
Succinate-semialdehyde dehydrogenase, mitochondrial	P51649	ALDH5A1	Mitochondrion.

Supplemental Table 1 Page 55. All proteins quantitated in study

Protein Name	Uniprot	Gene	Subcellular localization
NADH dehydrogenase [ubiquinone] 1 beta subcomplex subunit 5, mitochondrial	O43674	NDUFB5	Mitochondrion inner membrane {ECO:0000305|PubMed:12611891}; Single-pass membrane protein {ECO:0000305}; Matrix side {ECO:0000305}.
NADH dehydrogenase [ubiquinone] flavoprotein 1, mitochondrial	P49821	NDUFV1	Mitochondrion inner membrane; Peripheral membrane protein; Matrix side.
Deoxyribonuclease-1-like 1	P49184	DNASE1L1	Endoplasmic reticulum {ECO:0000269|PubMed:16107205}.
Glycerol-3-phosphate dehydrogenase 1-like protein	Q8N335	GPD1L	Cytoplasm {ECO:0000269|PubMed:17967977}. Note=Localized to the region of the plasma membrane.
Glyoxylate reductase/hydroxypyruvate reductase	Q9UBQ7	GRHPR	0
Aspartate aminotransferase, cytoplasmic	P17174	GOT1	Cytoplasm {ECO:0000269|PubMed:1974457}.
60S acidic ribosomal protein P2	P05387	RPLP2	0
Quinone oxidoreductase	Q08257	CRYZ	Cytoplasm {ECO:0000269|PubMed:20103721}.
Voltage-dependent calcium channel subunit alpha-2/delta-1	P54289	CACNA2D1	Membrane {ECO:0000305}; Single-pass type I membrane protein {ECO:0000305}.
14-3-3 protein zeta/delta	P63104	YWHAZ	Cytoplasm {ECO:0000269|PubMed:17081065}. Melanosome {ECO:0000269|PubMed:17081065}. Note=Located to stage I to stage IV melanosomes.
Dehydrogenase/reductase SDR family member 7C	A6NNS2	DHRS7C	Secreted {ECO:0000305}.
Cytochrome b-c1 complex subunit 7	P14927	UQCRB	Mitochondrion inner membrane.
Calcium uptake protein 2, mitochondrial	Q8IYU8	MICU2	Mitochondrion intermembrane space {ECO:0000269|PubMed:24231807, ECO:0000269|PubMed:24560927, ECO:0000269|PubMed:26774479, ECO:0000269|PubMed:27099988}.
Phosphatidate cytidylyltransferase 2	O95674	CDS2	Mitochondrion inner membrane {ECO:0000305}; Multi-pass membrane protein {ECO:0000305}; Matrix side {ECO:0000305}.
Malectin	Q14165	MLEC	Endoplasmic reticulum membrane {ECO:0000250}; Single-pass type I membrane protein {ECO:0000250}.
60 kDa heat shock protein, mitochondrial	P10809	HSPD1	Mitochondrion matrix.
Lon protease homolog, mitochondrial	P36776	LONP1	Mitochondrion matrix {ECO:0000255|HAMAP-Rule:MF_03120, ECO:0000269|PubMed:7961901}.
Keratin, type II cytoskeletal 1	P04264	KRT1	Cell membrane {ECO:0000269|PubMed:17956333}. Note=Located on plasma membrane of neuroblastoma NMB7 cells.

Supplemental Table 1 Page 56. All proteins quantitated in study

Protein Name	Uniprot	Gene	Subcellular localization
Aspartate aminotransferase, mitochondrial	P00505	GOT2	Mitochondrion matrix {ECO:0000269|PubMed:9537447}. Cell membrane {ECO:0000269|PubMed:9537447}. Note=Exposure to alcohol promotes translocation to the cell membrane.
Acetyl-coenzyme A synthetase 2-like, mitochondrial	Q9NUB1	ACSS1	Mitochondrion matrix {ECO:0000269|PubMed:16788062}.
Cytochrome c1, heme protein, mitochondrial	P08574	CYC1	Mitochondrion inner membrane; Single-pass membrane protein; Intermembrane side.
Barrier-to-autointegration factor	O75531	BANF1	Barrier-to-autointegration factor: Nucleus {ECO:0000269|PubMed:16495336, ECO:0000269|PubMed:24600006}. Cytoplasm {ECO:0000269|PubMed:16495336, ECO:0000269|PubMed:24600006}. Chromosome {ECO:0000269|PubMed:16495336}. Nucleus envelope {ECO:0000269|PubMed:24600006}. Note=Significantly enriched at the nuclear inner membrane, diffusely throughout the nucleus during interphase and concentrated at the chromosomes during the M-phase. The phosphorylated form (by VRK1 or vaccinia virus B1 kinase) shows a cytoplasmic localization whereas the unphosphorylated form locates almost exclusively in the nucleus (PubMed:24600006, PubMed:16495336). May be included in HIV-1 virions via its interaction with viral GAG polyprotein. {ECO:0000269|PubMed:16495336, ECO:0000269|PubMed:24600006}.
Sulfite oxidase, mitochondrial	P51687	SUOX	Mitochondrion intermembrane space.
NAD-dependent protein deacetylase sirtuin-3, mitochondrial	Q9NTG7	SIRT3	Mitochondrion matrix {ECO:0000269|PubMed:12186850, ECO:0000269|PubMed:12374852, ECO:0000269|PubMed:16079181, ECO:0000269|PubMed:18215119, ECO:0000269|PubMed:23283301, ECO:0000269|PubMed:29445193}.
ATP synthase subunit a	P00846	MT-ATP6	Mitochondrion inner membrane; Multi-pass membrane protein.
Hydroxyacyl-coenzyme A dehydrogenase, mitochondrial	Q16836	HADH	Mitochondrion matrix.
60S ribosomal protein L7	P18124	RPL7	0
Sodium/potassium-transporting ATPase subunit beta-1	P05026	ATP1B1	Cell membrane {ECO:0000255}; Single-pass type II membrane protein {ECO:0000255}. Cell membrane, sarcolemma {ECO:0000250|UniProtKB:P14094}. Note=Colocalizes with OBSCN at the intercalated disk and sarcolemma in cardiomyocytes. Localizes in long striations at the level of Z and M lines. {ECO:0000250|UniProtKB:P14094}.
ATP synthase F(0) complex subunit C2, mitochondrial	Q06055	ATP5MC2	Mitochondrion membrane; Multi-pass membrane protein.
Annexin A11	P50995	ANXA11	Cytoplasm {ECO:0000269|PubMed:28469040}. Melanosome. Nucleus envelope. Nucleus, nucleoplasm {ECO:0000269|PubMed:28469040}. Cytoplasm, cytoskeleton, spindle. Note=Found throughout the nucleoplasm at interphase and during mitosis concentrates around the mitotic apparatus (By similarity). Elevation of intracellular calcium causes relocalization from the nucleoplasm to the nuclear envelope, with little effect on the cytoplasmic pool. Localization to the nuclear envelope is cell-cycle dependent. {ECO:0000250}.

Supplemental Table 1 Page 57. All proteins quantitated in study

Protein Name	Uniprot	Gene	Subcellular localization
NADH dehydrogenase [ubiquinone] 1 alpha subcomplex subunit 5	Q16718	NDUFA5	Mitochondrion inner membrane {ECO:0000305|PubMed:12611891}; Peripheral membrane protein {ECO:0000305}; Matrix side {ECO:0000305}.
L-lactate dehydrogenase A chain	P00338	LDHA	Cytoplasm.
Electron transfer flavoprotein subunit beta	P38117	ETFB	Mitochondrion matrix {ECO:0000305|PubMed:25023281, ECO:0000305|PubMed:8504797}.
2-methoxy-6-polyprenyl-1,4-benzoquinol methylase, mitochondrial	Q5HYK3	COQ5	Mitochondrion inner membrane {ECO:0000255|HAMAP-Rule:MF_03191, ECO:0000269|PubMed:25152161}; Peripheral membrane protein {ECO:0000255|HAMAP-Rule:MF_03191, ECO:0000269|PubMed:25152161}; Matrix side {ECO:0000255|HAMAP-Rule:MF_03191, ECO:0000269|PubMed:25152161}.
Cytochrome c oxidase subunit 2	P00403	MT-CO2	Mitochondrion inner membrane; Multi-pass membrane protein.
Cell division control protein 42 homolog	P60953	CDC42	Cell membrane {ECO:0000305}; Lipid-anchor {ECO:0000305}; Cytoplasmic side {ECO:0000305}. Cytoplasm, cytoskeleton, microtubule organizing center, centrosome {ECO:0000269|PubMed:15642749}. Cytoplasm, cytoskeleton, spindle {ECO:0000269|PubMed:15642749}. Midbody {ECO:0000269|PubMed:15642749}. Note=Localizes to spindle during prometaphase cells. Moves to the central spindle as cells progressed through anaphase to telophase (PubMed:15642749). Localizes at the end of cytokinesis in the intercellular bridge formed between two daughter cells (PubMed:15642749). Its localization is regulated by the activities of guanine nucleotide exchange factor ECT2 and GTPase activating protein RACGAP1 (PubMed:15642749). Colocalizes with NEK6 in the centrosome (PubMed:20873783). In its active GTP-bound form localizes to the leading edge membrane of migrating dendritic cells (By similarity). {ECO:0000250|UniProtKB:P60766, ECO:0000269|PubMed:15642749, ECO:0000269|PubMed:20873783}.
Keratin, type II cytoskeletal 2 epidermal	P35908	KRT2	0
Cytochrome c oxidase subunit 6B1	P14854	COX6B1	Mitochondrion intermembrane space {ECO:0000250}.
NADH dehydrogenase [ubiquinone] 1 beta subcomplex subunit 3	O43676	NDUFB3	Mitochondrion inner membrane {ECO:0000305|PubMed:12611891}; Single-pass membrane protein {ECO:0000305}; Matrix side {ECO:0000305}.
5-demethoxyubiquinone hydroxylase, mitochondrial	Q99807	COQ7	Mitochondrion inner membrane {ECO:0000255|HAMAP-Rule:MF_03194}; Peripheral membrane protein {ECO:0000255|HAMAP-Rule:MF_03194}; Matrix side {ECO:0000255|HAMAP-Rule:MF_03194}.
ER membrane protein complex subunit 1	Q8N766	EMC1	Membrane {ECO:0000269|PubMed:22119785}; Single-pass type I membrane protein {ECO:0000269|PubMed:22119785}.

Supplemental Table 1 Page 58. All proteins quantitated in study

Protein Name	Uniprot	Gene	Subcellular localization
Histone H2AX	P16104	H2AFX	Nucleus {ECO:0000269|PubMed:12419185, ECO:0000269|PubMed:12660252, ECO:0000269|PubMed:12697768, ECO:0000269|PubMed:15613478, ECO:0000269|PubMed:24429368}. Chromosome {ECO:0000269|PubMed:10959836, ECO:0000269|PubMed:11673449, ECO:0000269|PubMed:12607005, ECO:0000269|PubMed:12660252, ECO:0000269|PubMed:15059890, ECO:0000269|PubMed:15201865, ECO:0000269|PubMed:15613478}.
Mitochondrial proton/calcium exchanger protein	O95202	LETM1	Mitochondrion inner membrane {ECO:0000269|PubMed:14706454, ECO:0000269|PubMed:15138253, ECO:0000269|PubMed:18628306, ECO:0000269|PubMed:19797662}; Single-pass membrane protein {ECO:0000269|PubMed:14706454, ECO:0000269|PubMed:15138253, ECO:0000269|PubMed:18628306}.
Transforming protein RhoA	P61586	RHOA	Cell membrane; Lipid-anchor; Cytoplasmic side. Cytoplasm, cytoskeleton. Cleavage furrow. Cytoplasm, cell cortex {ECO:0000269|PubMed:9635436}. Midbody. Cell projection, lamellipodium {ECO:0000250}. Note=Localized to cell-cell contacts in calcium-treated keratinocytes (By similarity). Translocates to the equatorial region before furrow formation in a ECT2-dependent manner. Localizes to the equatorial cell cortex (at the site of the presumptive furrow) in early anaphase in an activated form and in a myosin- and actin-independent manner. {ECO:0000250}.
Calcium/calmodulin-dependent protein kinase type II subunit delta	Q13557	CAMK2D	Cell membrane, sarcolemma {ECO:0000305}; Peripheral membrane protein {ECO:0000305}; Cytoplasmic side {ECO:0000305}. Sarcoplasmic reticulum membrane {ECO:0000305}; Peripheral membrane protein {ECO:0000305}; Cytoplasmic side {ECO:0000305}.
NAD(P) transhydrogenase, mitochondrial	Q13423	NNT	Mitochondrion inner membrane {ECO:0000305}; Multi-pass membrane protein {ECO:0000305}; Matrix side {ECO:0000305}.
Isocitrate dehydrogenase [NADP], mitochondrial	P48735	IDH2	Mitochondrion.
Hydroxyacid-oxoacid transhydrogenase, mitochondrial	Q8IWW8	ADHFE1	Mitochondrion {ECO:0000250}.
Nucleolin	P19338	NCL	Nucleus, nucleolus. Cytoplasm. Note=Localized in cytoplasmic mRNP granules containing untranslated mRNAs.
Calnexin	P27824	CANX	Endoplasmic reticulum membrane {ECO:0000269|PubMed:22314232}; Single-pass type I membrane protein {ECO:0000255}. Endoplasmic reticulum {ECO:0000269|PubMed:22314232}. Melanosome {ECO:0000269|PubMed:12643545, ECO:0000269|PubMed:17081065}. Note=Identified by mass spectrometry in melanosome fractions from stage I to stage IV (PubMed:12643545, PubMed:17081065). The palmitoylated form preferentially localizes to the perinuclear rough ER (PubMed:22314232). {ECO:0000269|PubMed:12643545, ECO:0000269|PubMed:17081065, ECO:0000269|PubMed:22314232}.

Supplemental Table 1 Page 59. All proteins quantitated in study

Protein Name	Uniprot	Gene	Subcellular localization
Histone H1.0	P07305	H1F0	Nucleus {ECO:0000255|PROSITE-ProRule:PRU00837, ECO:0000269|PubMed:18993075}. Chromosome {ECO:0000255|PROSITE-ProRule:PRU00837, ECO:0000269|PubMed:18993075}. Note=The RNA edited version has been localized to nuclear speckles. During mitosis, it appears in the vicinity of condensed chromosomes.
High mobility group protein B1	P09429	HMGB1	Nucleus {ECO:0000269|PubMed:12231511, ECO:0000269|PubMed:17114460, ECO:0000269|PubMed:20819940, ECO:0000269|PubMed:22869893, ECO:0000269|PubMed:27362237}. Chromosome {ECO:0000250|UniProtKB:P10103, ECO:0000250|UniProtKB:P63159, ECO:0000305}. Cytoplasm {ECO:0000269|PubMed:11154118, ECO:0000269|PubMed:12231511, ECO:0000269|PubMed:17114460, ECO:0000269|PubMed:20819940, ECO:0000269|PubMed:22869893}. Secreted {ECO:0000250|UniProtKB:P63158, ECO:0000269|PubMed:12231511, ECO:0000269|PubMed:14532127, ECO:0000269|PubMed:15944249, ECO:0000269|PubMed:19811284, ECO:0000269|PubMed:22869893}. Cell membrane {ECO:0000250|UniProtKB:P63158, ECO:0000250|UniProtKB:P63159, ECO:0000269|PubMed:11154118}; Peripheral membrane protein {ECO:0000250|UniProtKB:P63158, ECO:0000250|UniProtKB:P63159, ECO:0000269|PubMed:11154118}; Extracellular side {ECO:0000250|UniProtKB:P63158, ECO:0000250|UniProtKB:P63159, ECO:0000269|PubMed:11154118}. Endosome {ECO:0000250|UniProtKB:P63158}. Endoplasmic reticulum-Golgi intermediate compartment {ECO:0000250|UniProtKB:P63158}. Note=In basal state predominantly nuclear. Shuttles between the cytoplasm and the nucleus (PubMed:12231511, PubMed:17114460). Translocates from the nucleus to the cytoplasm upon autophagy stimulation (PubMed:20819940). Release from macrophages in the extracellular milieu requires the activation of NLRC4 or NLRP3 inflammasomes (By similarity). Passively released to the extracellular milieu from necrotic cells by diffusion, involving the fully reduced HGMB1 which subsequently gets oxidized (PubMed:19811284). Also released from apoptotic cells (PubMed:16855214, PubMed:18631454). Active secretion from a variety of immune and non-immune cells such as macrophages, monocytes, neutrophils, dendritic cells and natural killer cells in response to various stimuli such as LPS and cytokines involves a nonconventional secretory process via secretory lysosomes (PubMed:12231511, PubMed:14532127, PubMed:15944249). Secreted by plasma cells in response to LPS (By similarity). Found on the surface of activated platelets (PubMed:11154118). An increased chromatin association is observed when associated with the adenovirus protein pVII (PubMed:27362237). {ECO:0000250|UniProtKB:P63158, ECO:0000269|PubMed:11154118, ECO:0000269|PubMed:12231511, ECO:0000269|PubMed:14532127, ECO:0000269|PubMed:15944249, ECO:0000269|PubMed:16855214, ECO:0000269|PubMed:17114460, ECO:0000269|PubMed:18631454, ECO:0000269|PubMed:19811284, ECO:0000269|PubMed:20819940, ECO:0000269|PubMed:27362237, ECO:0000305|PubMed:20123072}.

Supplemental Table 1 Page 60. All proteins quantitated in study

Protein Name	Uniprot	Gene	Subcellular localization
Voltage-dependent anion-selective channel protein 1	P21796	VDAC1	Mitochondrion outer membrane {ECO:0000269|PubMed:7539795}; Multi-pass membrane protein {ECO:0000269|PubMed:18755977, ECO:0000269|PubMed:18832158}. Cell membrane {ECO:0000269|PubMed:25168729, ECO:0000269|PubMed:25296756}; Multi-pass membrane protein {ECO:0000269|PubMed:18755977, ECO:0000269|PubMed:18832158}. Membrane raft {ECO:0000269|PubMed:25168729}; Multi-pass membrane protein {ECO:0000269|PubMed:18755977, ECO:0000269|PubMed:18832158}.
3-hydroxyisobutyrate dehydrogenase, mitochondrial	P31937	HIBADH	Mitochondrion.
39S ribosomal protein L12, mitochondrial	P52815	MRPL12	Mitochondrion {ECO:0000269|PubMed:8626705}.
ATP-dependent RNA helicase DDX3X	O00571	DDX3X	Nucleus speckle. Cytoplasm. Mitochondrion outer membrane. Note=Located predominantly in nuclear speckles and, at low levels, throughout the cytoplasm. Located to the outer side of nuclear pore complexes (NPC). Shuttles between the nucleus and the cytoplasm in a XPO1 and may be also in a NFX1-dependent manner. Associated with polyadenylated mRNAs in the cytoplasm and the nucleus. Predominantly located in nucleus during G(0) phase and in the cytoplasm during G1/S phase.
40S ribosomal protein S5	P46782	RPS5	0
Beta-actin-like protein 2	Q562R1	ACTBL2	Cytoplasm, cytoskeleton {ECO:0000250}.
NADH dehydrogenase [ubiquinone] 1 alpha subcomplex subunit 13	Q9P0J0	NDUFA13	Mitochondrion inner membrane {ECO:0000269|PubMed:12628925, ECO:0000269|PubMed:15059901, ECO:0000269|PubMed:15367666}; Single-pass membrane protein {ECO:0000255}; Matrix side. Nucleus {ECO:0000269|PubMed:12628925}. Note=Localizes mainly in the mitochondrion (PubMed:12628925). May be translocated into the nucleus upon IFN/RA treatment. {ECO:0000269|PubMed:12628925, ECO:0000269|PubMed:15059901}.
4-trimethylaminobutyraldehyde dehydrogenase	P49189	ALDH9A1	Cytoplasm {ECO:0000250}.
Cytoskeleton-associated protein 4	Q07065	CKAP4	Endoplasmic reticulum membrane; Single-pass type II membrane protein. Cell membrane; Single-pass type II membrane protein. Cytoplasm, cytoskeleton. Cytoplasm, perinuclear region. Note=Translocates to the perinuclear region upon APF-stimulation.
Cytochrome b-c1 complex subunit 2, mitochondrial	P22695	UQCRC2	Mitochondrion inner membrane; Peripheral membrane protein; Matrix side.
Succinate dehydrogenase [ubiquinone] flavoprotein subunit, mitochondrial	P31040	SDHA	Mitochondrion inner membrane {ECO:0000250|UniProtKB:Q0QF01}; Peripheral membrane protein {ECO:0000250|UniProtKB:Q0QF01}; Matrix side {ECO:0000250|UniProtKB:Q0QF01}.
Spectrin beta chain, erythrocytic	P11277	SPTB	Cytoplasm, cytoskeleton. Cytoplasm, cell cortex.

Supplemental Table 1 Page 61. All proteins quantitated in study

Protein Name	Uniprot	Gene	Subcellular localization
Transitional endoplasmic reticulum ATPase	P55072	VCP	Cytoplasm, cytosol {ECO:0000269|PubMed:15456787}. Endoplasmic reticulum {ECO:0000269|PubMed:15215856}. Nucleus {ECO:0000269|PubMed:23042605, ECO:0000269|PubMed:26842564}. Cytoplasm, Stress granule {ECO:0000269|PubMed:29804830}. Note=Present in the neuronal hyaline inclusion bodies specifically found in motor neurons from amyotrophic lateral sclerosis patients (PubMed:15456787). Present in the Lewy bodies specifically found in neurons from Parkinson disease patients (PubMed:15456787). Recruited to the cytoplasmic surface of the endoplasmic reticulum via interaction with AMFR/gp78 (PubMed:16168377). Following DNA double-strand breaks, recruited to the sites of damage (PubMed:22120668). Recruited to stalled replication forks via interaction with SPRTN (PubMed:23042605). Recruited to damaged lysosomes decorated with K48-linked ubiquitin chains (PubMed:27753622). Colocalizes with TIA1, ZFAND1 and G3BP1 in cytoplasmic stress granules (SGs) in response to arsenite-induced stress treatment (PubMed:29804830). {ECO:0000269|PubMed:15456787, ECO:0000269|PubMed:16168377, ECO:0000269|PubMed:22120668, ECO:0000269|PubMed:23042605, ECO:0000269|PubMed:27753622, ECO:0000269|PubMed:29804830}.
Mitochondrial import inner membrane translocase subunit TIM50	Q3ZCQ8	TIMM50	Mitochondrion inner membrane {ECO:0000269|PubMed:15044455}; Single-pass membrane protein {ECO:0000269|PubMed:15044455}.; Isoform 2: Nucleus speckle {ECO:0000269|PubMed:16008839}. Note=Nuclear and enriched in speckles with snRNPs. {ECO:0000269|PubMed:16008839}.
Dystrobrevin alpha	Q9Y4J8	DTNA	Cytoplasm. Cell junction, synapse. Cell membrane {ECO:0000250}. Note=In peripheral nerves, colocalizes with MAGEE1 in the Schwann cell membrane. {ECO:0000250}.
Fatty acid-binding protein, heart	P05413	FABP3	Cytoplasm.
Acyl-CoA dehydrogenase family member 9, mitochondrial	Q9H845	ACAD9	Mitochondrion {ECO:0000269|PubMed:20816094}.
Leucine-rich PPR motif-containing protein, mitochondrial	P42704	LRPPRC	Mitochondrion. Nucleus, nucleoplasm. Nucleus inner membrane. Nucleus outer membrane. Note=Seems to be predominantly mitochondrial.
Prostaglandin reductase 3	Q8N4Q0	ZADH2	Peroxisome {ECO:0000250|UniProtKB:Q8BGC4}.
Transmembrane protein 205	Q6UW68	TMEM205	Membrane {ECO:0000269|PubMed:20589834}; Multi-pass membrane protein {ECO:0000269|PubMed:20589834}. Note=Located on cell surface microvilli. In cancer cells, transition in subcellular location from cell surface to intracellular regions correlates the progression of cisplatin resistance.
Aldehyde dehydrogenase, mitochondrial	P05091	ALDH2	Mitochondrion matrix.

Supplemental Table 1 Page 62. All proteins quantitated in study

Protein Name	Uniprot	Gene	Subcellular localization
Flotillin-1	O75955	FLOT1	Cell membrane; Peripheral membrane protein {ECO:0000269|PubMed:20682791}. Endosome {ECO:0000269|PubMed:20682791}. Membrane, caveola {ECO:0000250|UniProtKB:O08917}; Peripheral membrane protein {ECO:0000250|UniProtKB:O08917}. Melanosome {ECO:0000269|PubMed:17081065}. Membrane raft {ECO:0000269|PubMed:25893292}. Note=Identified by mass spectrometry in melanosome fractions from stage I to stage IV (PubMed:17081065). Membrane-associated protein of caveola (By similarity). {ECO:0000250|UniProtKB:O08917, ECO:0000269|PubMed:17081065}.
Methylmalonyl-CoA mutase, mitochondrial	P22033	MMUT	Mitochondrion matrix.
Propionyl-CoA carboxylase beta chain, mitochondrial	P05166	PCCB	Mitochondrion matrix {ECO:0000269|PubMed:16023992}.
Platelet glycoprotein 4	P16671	CD36	Cell membrane {ECO:0000269|PubMed:16880211}; Multi-pass membrane protein {ECO:0000255}. Membrane raft {ECO:0000269|PubMed:16880211}. Golgi apparatus {ECO:0000269|PubMed:16880211}. Apical cell membrane {ECO:0000250|UniProtKB:Q08857}. Note=Upon ligand-binding, internalized through dynamin-dependent endocytosis. {ECO:0000269|PubMed:20037584}.
Band 3 anion transport protein	P02730	SLC4A1	Cell membrane {ECO:0000269|PubMed:10926824, ECO:0000269|PubMed:24121512, ECO:0000269|PubMed:26542571, ECO:0000269|PubMed:7506871}; Multi-pass membrane protein {ECO:0000269|PubMed:26542571}. Basolateral cell membrane {ECO:0000269|PubMed:7506871}; Multi-pass membrane protein {ECO:0000269|PubMed:7506871}. Note=Detected in the erythrocyte cell membrane and on the basolateral membrane of alpha-intercalated cells in the collecting duct in the kidney. {ECO:0000269|PubMed:7506871}.
Membrane-associated progesterone receptor component 2	O15173	PGRMC2	Membrane {ECO:0000305}; Single-pass membrane protein {ECO:0000305}.
Erythrocyte membrane protein band 4.2	P16452	EPB42	Cell membrane; Lipid-anchor; Cytoplasmic side. Cytoplasm, cytoskeleton. Note=Cytoplasmic surface of erythrocyte membranes.

Supplemental Table 1 Page 63. All proteins quantitated in study

Protein Name	Uniprot	Gene	Subcellular localization
Ras-related protein Rab-10	P61026	RAB10	Cytoplasmic vesicle membrane {ECO:0000305}; Lipid-anchor {ECO:0000305}; Cytoplasmic side {ECO:0000305}. Golgi apparatus membrane {ECO:0000269|PubMed:23263280}. Golgi apparatus, trans-Golgi network membrane {ECO:0000250|UniProtKB:P24409}. Endosome membrane {ECO:0000269|PubMed:16641372}. Recycling endosome membrane {ECO:0000250|UniProtKB:P24409}. Cytoplasmic vesicle, phagosome membrane {ECO:0000250|UniProtKB:P24409}. Cell projection, cilium {ECO:0000269|PubMed:20576682}. Endoplasmic reticulum membrane {ECO:0000269|PubMed:23263280}. Cytoplasm, perinuclear region {ECO:0000250|UniProtKB:P61027}. Note=Associates with SLC2A4/GLUT4 storage vesicles (PubMed:22908308). Localizes to the base of the cilium (PubMed:20576682). Transiently associates with phagosomes (By similarity). Localizes to the endoplasmic reticulum at domains of new tubule growth (PubMed:23263280). {ECO:0000250|UniProtKB:P24409, ECO:0000269|PubMed:20576682, ECO:0000269|PubMed:22908308, ECO:0000269|PubMed:23263280}.
Dolichyl-diphosphooligosaccharide--protein glycosyltransferase subunit 1	P04843	RPN1	Endoplasmic reticulum {ECO:0000250|UniProtKB:E2RQ08, ECO:0000250|UniProtKB:Q9GMB0}. Endoplasmic reticulum membrane; Single-pass type I membrane protein {ECO:0000305}. Melanosome. Note=Identified by mass spectrometry in melanosome fractions from stage I to stage IV.
Enoyl-CoA delta isomerase 1, mitochondrial	P42126	ECI1	Mitochondrion matrix.
Estradiol 17-beta-dehydrogenase 8	Q92506	HSD17B8	Mitochondrion matrix {ECO:0000269|PubMed:19571038}.
Delta(3,5)-Delta(2,4)-dienoyl-CoA isomerase, mitochondrial	Q13011	ECH1	Mitochondrion {ECO:0000250}. Peroxisome {ECO:0000250}.
Sodium/potassium-transporting ATPase subunit alpha-2	P50993	ATP1A2	Membrane {ECO:0000269|PubMed:7711835}; Multi-pass membrane protein {ECO:0000269|PubMed:7711835}. Cell membrane {ECO:0000269|PubMed:7711835}; Multi-pass membrane protein {ECO:0000269|PubMed:7711835}.
Catechol O-methyltransferase domain-containing protein 1	Q86VU5	COMTD1	Membrane {ECO:0000305}; Single-pass type II membrane protein {ECO:0000305}.
Keratin, type I cytoskeletal 10	P13645	KRT10	Secreted, extracellular space {ECO:0000269|PubMed:12427098}. Cell surface {ECO:0000269|PubMed:19627498}. Note=Localized on the surface of desquamated nasal epithelial cells (PubMed:12427098). Localized on the surface of lung cell lines (PubMed:19627498). {ECO:0000269|PubMed:12427098, ECO:0000269|PubMed:19627498}.
Alpha-crystallin B chain	P02511	CRYAB	Cytoplasm {ECO:0000269|PubMed:19464326, ECO:0000269|PubMed:28493373}. Nucleus {ECO:0000269|PubMed:19464326}. Note=Translocates to the nucleus during heat shock and resides in sub-nuclear structures known as SC35 speckles or nuclear splicing speckles (PubMed:19464326). Localizes at the Z-bands and the intercalated disk in cardiomyocytes (PubMed:28493373). {ECO:0000269|PubMed:19464326, ECO:0000269|PubMed:28493373}.

Supplemental Table 1 Page 64. All proteins quantitated in study

Protein Name	Uniprot	Gene	Subcellular localization
Inorganic pyrophosphatase 2, mitochondrial	Q9H2U2	PPA2	Mitochondrion {ECO:0000305}.
NADH dehydrogenase [ubiquinone] iron-sulfur protein 7, mitochondrial	O75251	NDUFS7	Mitochondrion {ECO:0000305|PubMed:12611891}.
Cytosol aminopeptidase	P28838	LAP3	Cytoplasm.
NADH dehydrogenase [ubiquinone] 1 beta subcomplex subunit 1	O75438	NDUFB1	Mitochondrion inner membrane {ECO:0000305|PubMed:12611891}; Single-pass membrane protein {ECO:0000255}; Matrix side {ECO:0000305}.
Iron-sulfur protein NUBPL	Q8TB37	NUBPL	Mitochondrion {ECO:0000269|PubMed:19752196}.
Very long-chain specific acyl-CoA dehydrogenase, mitochondrial	P49748	ACADVL	Mitochondrion inner membrane.
Complement component 1 Q subcomponent-binding protein, mitochondrial	Q07021	C1QBP	Mitochondrion matrix {ECO:0000269|PubMed:15031724, ECO:0000269|PubMed:17486078, ECO:0000269|PubMed:19164550, ECO:0000269|PubMed:9305894}. Nucleus {ECO:0000269|PubMed:18676636}. Cell membrane {ECO:0000269|PubMed:10747014, ECO:0000269|PubMed:11493647, ECO:0000269|PubMed:12574814, ECO:0000269|PubMed:8195709, ECO:0000269|PubMed:8662673, ECO:0000269|PubMed:9191880, ECO:0000269|PubMed:9233640}; Peripheral membrane protein {ECO:0000269|PubMed:12574814, ECO:0000269|PubMed:8662673, ECO:0000269|PubMed:9191880}; Extracellular side. Secreted. Cytoplasm {ECO:0000269|PubMed:11493647}. Nucleus, nucleolus {ECO:0000269|PubMed:21536856}. Note=Seems to be predominantly localized to mitochondria. Secreted by activated lymphocytes.
Dihydroorotate dehydrogenase (quinone), mitochondrial	Q02127	DHODH	Mitochondrion inner membrane {ECO:0000269|PubMed:10727948}; Single-pass membrane protein {ECO:0000269|PubMed:10727948}.
Reticulon-4-interacting protein 1, mitochondrial	Q8WWV3	RTN4IP1	Mitochondrion outer membrane {ECO:0000269|PubMed:12067236, ECO:0000269|PubMed:26593267}. Note=Colocalizes with the endoplasmic reticulum HSPA5 at spots corresponding to contacts with mitochondria. {ECO:0000269|PubMed:26593267}.
Fatty aldehyde dehydrogenase	P51648	ALDH3A2	Microsome membrane {ECO:0000269|PubMed:9133646}; Single-pass membrane protein {ECO:0000305|PubMed:25047030, ECO:0000305|PubMed:9133646}. Endoplasmic reticulum membrane {ECO:0000305|PubMed:9133646}; Single-pass membrane protein {ECO:0000305|PubMed:25047030, ECO:0000305|PubMed:9133646}; Cytoplasmic side {ECO:0000250|UniProtKB:P30839}.
Sorting and assembly machinery component 50 homolog	Q9Y512	SAMM50	Mitochondrion outer membrane {ECO:0000269|PubMed:15644312}; Multi-pass membrane protein. Cytoplasm {ECO:0000250|UniProtKB:Q6AXV4}. Mitochondrion {ECO:0000269|PubMed:25781180}.
Methylcrotonoyl-CoA carboxylase beta chain, mitochondrial	Q9HCC0	MCCC2	Mitochondrion matrix {ECO:0000269|PubMed:11170888, ECO:0000269|PubMed:16023992}.
Fibrinogen alpha chain	P02671	FGA	Secreted {ECO:0000269|PubMed:19296670, ECO:0000269|PubMed:9628725}.

Supplemental Table 1 Page 65. All proteins quantitated in study

Protein Name	Uniprot	Gene	Subcellular localization
Methylcrotonoyl-CoA carboxylase subunit alpha, mitochondrial	Q96RQ3	MCCC1	Mitochondrion matrix {ECO:0000269|PubMed:16023992}.
Short-chain specific acyl-CoA dehydrogenase, mitochondrial	P16219	ACADS	Mitochondrion matrix.
40S ribosomal protein S10	P46783	RPS10	Cytoplasm {ECO:0000269|PubMed:20159986}. Nucleus, nucleolus {ECO:0000269|PubMed:20159986}. Note=Localized in the granular component (GC) region of the nucleolus. Methylation is required for its localization in the GC region. Colocalizes with NPS1 in the GC region of the nucleolus.
Protein/nucleic acid deglycase DJ-1	Q99497	PARK7	Cell membrane {ECO:0000250|UniProtKB:Q99LX0}; Lipid-anchor {ECO:0000250|UniProtKB:Q99LX0}. Cytoplasm {ECO:0000269|PubMed:12851414, ECO:0000269|PubMed:14579415, ECO:0000269|PubMed:15976810, ECO:0000269|PubMed:19229105, ECO:0000269|PubMed:28596309}. Nucleus {ECO:0000269|PubMed:12851414, ECO:0000269|PubMed:14579415, ECO:0000269|PubMed:15976810, ECO:0000269|PubMed:16390825, ECO:0000269|PubMed:28596309}. Membrane raft {ECO:0000250|UniProtKB:O88767}. Mitochondrion {ECO:0000269|PubMed:15181200, ECO:0000269|PubMed:18711745, ECO:0000269|PubMed:19229105}. Note=Under normal conditions, located predominantly in the cytoplasm and, to a lesser extent, in the nucleus and mitochondrion. Translocates to the mitochondrion and subsequently to the nucleus in response to oxidative stress and exerts an increased cytoprotective effect against oxidative damage (PubMed:18711745). Detected in tau inclusions in brains from neurodegenerative disease patients (PubMed:14705119). Membrane raft localization in astrocytes and neuronal cells requires palmitoylation. {ECO:0000269|PubMed:14705119, ECO:0000269|PubMed:18711745}.
NADH dehydrogenase [ubiquinone] iron-sulfur protein 4, mitochondrial	O43181	NDUFS4	Mitochondrion inner membrane {ECO:0000269|PubMed:12611891}; Peripheral membrane protein {ECO:0000269|PubMed:12611891}; Matrix side {ECO:0000269|PubMed:12611891}.
Erlin-2	O94905	ERLIN2	Endoplasmic reticulum membrane {ECO:0000269|PubMed:16835267, ECO:0000269|PubMed:17502376, ECO:0000269|PubMed:19240031}; Single-pass type II membrane protein {ECO:0000269|PubMed:16835267, ECO:0000269|PubMed:17502376, ECO:0000269|PubMed:19240031}. Note=Associated with lipid raft-like domains of the endoplasmic reticulum membrane.
Triosephosphate isomerase	P60174	TPI1	Cytoplasm {ECO:0000255|PROSITE-ProRule:PRU10127}.
Acyl-CoA synthetase family member 2, mitochondrial	Q96CM8	ACSF2	Mitochondrion {ECO:0000305}.

Supplemental Table 1 Page 66. All proteins quantitated in study

Protein Name	Uniprot	Gene	Subcellular localization
PRA1 family protein 3	O75915	ARL6IP5	Endoplasmic reticulum membrane {ECO:0000250|UniProtKB:Q9ES40}; Multi-pass membrane protein {ECO:0000255}. Cell membrane {ECO:0000250|UniProtKB:Q9ES40}; Multi-pass membrane protein {ECO:0000255}. Cytoplasm {ECO:0000250|UniProtKB:Q9ES40}. Cytoplasm, cytoskeleton {ECO:0000250|UniProtKB:Q9ES40}. Note=Also exists as a soluble form in the cytoplasm. Associated with microtubules. {ECO:0000250|UniProtKB:Q9ES40}.
Stress-70 protein, mitochondrial	P38646	HSPA9	Mitochondrion {ECO:0000269|PubMed:22002106, ECO:0000269|PubMed:26702583}. Nucleus, nucleolus {ECO:0000269|PubMed:22002106}.
Endoplasmic reticulum aminopeptidase 1	Q9NZ08	ERAP1	Endoplasmic reticulum membrane {ECO:0000305|PubMed:15908954}; Single-pass type II membrane protein {ECO:0000305|PubMed:15908954}.
Neutral alpha-glucosidase AB	Q14697	GANAB	Endoplasmic reticulum {ECO:0000305|PubMed:10929008}. Golgi apparatus {ECO:0000250|UniProtKB:P79403}. Melanosome {ECO:0000269|PubMed:17081065}. Note=Identified by mass spectrometry in melanosome fractions from stage I to stage IV. {ECO:0000269|PubMed:17081065}.
Serine--tRNA ligase, mitochondrial	Q9NP81	SARS2	Mitochondrion matrix {ECO:0000250|UniProtKB:Q9N0F3}.
Ubiquinone biosynthesis protein COQ9, mitochondrial	O75208	COQ9	Mitochondrion {ECO:0000250|UniProtKB:Q8K1Z0}.
Transmembrane protein 11, mitochondrial	P17152	TMEM11	Mitochondrion inner membrane {ECO:0000269|PubMed:21274005}; Multi-pass membrane protein {ECO:0000269|PubMed:21274005}.
Cytosolic 5'-nucleotidase 1A	Q9BXI3	NT5C1A	Cytoplasm.
Inactive hydroxysteroid dehydrogenase-like protein 1	Q3SXM5	HSDL1	Mitochondrion {ECO:0000269|PubMed:19026618}.
DnaJ homolog subfamily C member 11	Q9NVH1	DNAJC11	Mitochondrion {ECO:0000269|PubMed:25997101}. Note=Isoforms show differential submitochondrial localization. A 57 kDa form (potentially isoform 3) shows either mitochondrial matrix or innermembrane (IM) localization, possibly anchored to the IM facing the matrix. A 35 kDa form behaved either as an inner membrane space (IMS) or an IM protein exposed to the IMS. {ECO:0000269|PubMed:25111180}.; Isoform 1: Mitochondrion outer membrane {ECO:0000269|PubMed:25111180}; Peripheral membrane protein {ECO:0000269|PubMed:25111180}.
78 kDa glucose-regulated protein	P11021	HSPA5	Endoplasmic reticulum lumen {ECO:0000269|PubMed:21080038, ECO:0000269|PubMed:21289099, ECO:0000269|PubMed:23990668, ECO:0000269|PubMed:29497057}. Melanosome {ECO:0000269|PubMed:12643545}. Cytoplasm {ECO:0000250|UniProtKB:P20029}. Note=Identified by mass spectrometry in melanosome fractions from stage I to stage IV. {ECO:0000269|PubMed:12643545}.
3-hydroxyisobutyryl-CoA hydrolase, mitochondrial	Q6NVY1	HIBCH	Mitochondrion {ECO:0000250}.
OCIA domain-containing protein 1	Q9NX40	OCIAD1	Endosome {ECO:0000250|UniProtKB:Q9CRD0}.

Supplemental Table 1 Page 67. All proteins quantitated in study

Protein Name	Uniprot	Gene	Subcellular localization
Stomatin-like protein 2, mitochondrial	Q9UJZ1	STOML2	Cell membrane {ECO:0000269|PubMed:10713127, ECO:0000269|PubMed:18641330, ECO:0000269|PubMed:21746876, ECO:0000269|PubMed:22623988}; Peripheral membrane protein {ECO:0000269|PubMed:10713127}. Mitochondrion {ECO:0000269|PubMed:18641330, ECO:0000269|PubMed:19597348, ECO:0000269|PubMed:22623988}. Mitochondrion inner membrane {ECO:0000269|PubMed:17121834, ECO:0000269|PubMed:18339324, ECO:0000269|PubMed:21746876}; Lipid-anchor {ECO:0000269|PubMed:21746876}. Mitochondrion intermembrane space {ECO:0000269|PubMed:17121834}. Membrane raft {ECO:0000269|PubMed:18641330}. Cytoplasm, cytoskeleton {ECO:0000269|PubMed:10713127, ECO:0000269|PubMed:18641330}. Note=Behaves as an integral membrane protein of the mitochondrion despite the absence of a detectable transmembrane domain (PubMed:21746876). Also associates with the actin cytoskeleton and membrane rafts in activated T-cells (PubMed:18641330, PubMed:10713127). A minor pool is associated with the plasma membrane and is enriched at the immunological synapse in activated T-cells (PubMed:22623988). {ECO:0000269|PubMed:10713127, ECO:0000269|PubMed:18641330, ECO:0000269|PubMed:21746876, ECO:0000269|PubMed:22623988}.
MICOS complex subunit MIC26	Q9BUR5	APOO	Mitochondrion inner membrane {ECO:0000269|PubMed:25764979}; Single-pass membrane protein {ECO:0000255}. Secreted {ECO:0000269|PubMed:16956892, ECO:0000269|PubMed:25764979}. Mitochondrion {ECO:0000269|PubMed:25781180}. Golgi apparatus membrane {ECO:0000269|PubMed:25764979}. Endoplasmic reticulum membrane {ECO:0000269|PubMed:25764979}. Note=Exists in three distinct forms: a glycosylated and secreted form, an ER/Golgi-resident form and a non-glycosylated mitochondrial form. {ECO:0000269|PubMed:25764979}.
Short/branched chain specific acyl-CoA dehydrogenase, mitochondrial	P45954	ACADSB	Mitochondrion matrix.
Methylmalonate-semialdehyde dehydrogenase [acylating], mitochondrial	Q02252	ALDH6A1	Mitochondrion.
60S acidic ribosomal protein P1	P05386	RPLP1	0
NADH dehydrogenase [ubiquinone] 1 alpha subcomplex subunit 9, mitochondrial	Q16795	NDUFA9	Mitochondrion matrix {ECO:0000305|PubMed:12611891}.
Probable 2-oxoglutarate dehydrogenase E1 component DHKTD1, mitochondrial	Q96HY7	DHTKD1	Mitochondrion {ECO:0000269|PubMed:23141294}.

Supplemental Table 1 Page 68. All proteins quantitated in study

Protein Name	Uniprot	Gene	Subcellular localization
Fumarate hydratase, mitochondrial	P07954	FH	Isoform Mitochondrial: Mitochondrion {ECO:0000269|PubMed:27037871}.; Isoform Cytoplasmic: Cytoplasm, cytosol {ECO:0000269|PubMed:20231875, ECO:0000269|PubMed:22509282, ECO:0000269|PubMed:26237645, ECO:0000269|PubMed:27037871}. Nucleus {ECO:0000269|PubMed:20231875, ECO:0000269|PubMed:26237645}. Chromosome {ECO:0000269|PubMed:26237645}. Note=Translocates to the nucleus in response to DNA damage: localizes to DNA double-strand breaks (DSBs) following phosphorylation by PRKDC. {ECO:0000269|PubMed:26237645}.
Calsequestrin-2	O14958	CASQ2	Sarcoplasmic reticulum lumen {ECO:0000250|UniProtKB:O09161}. Note=This isoform of calsequestrin occurs in the sarcoplasmic reticulum's terminal cisternae luminal spaces of cardiac and slow skeletal muscle cells. {ECO:0000250|UniProtKB:O09161}.
Mitochondrial import receptor subunit TOM22 homolog	Q9NS69	TOMM22	Mitochondrion outer membrane {ECO:0000269|PubMed:10982837}; Single-pass membrane protein {ECO:0000269|PubMed:10982837}.
Cytochrome c	P99999	CYCS	Mitochondrion intermembrane space. Note=Loosely associated with the inner membrane.
Extended synaptotagmin-1	Q9BSJ8	ESYT1	Endoplasmic reticulum membrane {ECO:0000269|PubMed:29469807}; Multi-pass membrane protein. Cell membrane {ECO:0000269|PubMed:29469807}; Peripheral membrane protein. Note=Localizes primarily to the endoplasmic reticulum. Recruited to sites of contact between the endoplasmic reticulum and the cell membrane in response to increased cytosolic calcium levels. {ECO:0000269|PubMed:29469807}.
NADH-ubiquinone oxidoreductase chain 1	P03886	MT-ND1	Mitochondrion inner membrane {ECO:0000250}; Multi-pass membrane protein {ECO:0000250}.
Carnitine O-acetyltransferase	P43155	CRAT	Endoplasmic reticulum {ECO:0000305}. Peroxisome {ECO:0000305}. Mitochondrion inner membrane {ECO:0000305}; Peripheral membrane protein {ECO:0000305}; Matrix side {ECO:0000305}.; Isoform 1: Mitochondrion {ECO:0000305}.; Isoform 2: Peroxisome {ECO:0000305}.
Beta-sarcoglycan	Q16585	SGCB	Cell membrane, sarcolemma {ECO:0000250}; Single-pass type II membrane protein {ECO:0000250}. Cytoplasm, cytoskeleton {ECO:0000250}.
Rab GDP dissociation inhibitor beta	P50395	GDI2	Cytoplasm {ECO:0000250}. Membrane {ECO:0000250}; Peripheral membrane protein {ECO:0000250}.
Transcription factor A, mitochondrial	Q00059	TFAM	Mitochondrion {ECO:0000269|PubMed:1737790, ECO:0000269|PubMed:18063578, ECO:0000269|PubMed:22841477, ECO:0000269|PubMed:29445193}. Mitochondrion matrix, mitochondrion nucleoid {ECO:0000269|PubMed:18063578}.
T-complex protein 1 subunit epsilon	P48643	CCT5	Cytoplasm {ECO:0000269|PubMed:20080638}. Cytoplasm, cytoskeleton, microtubule organizing center, centrosome {ECO:0000269|PubMed:14654843, ECO:0000269|PubMed:20080638}.
Histidine triad nucleotide-binding protein 2, mitochondrial	Q9BX68	HINT2	Mitochondrion {ECO:0000269|PubMed:16762638, ECO:0000269|PubMed:18653718}.
Trimethyllysine dioxygenase, mitochondrial	Q9NVH6	TMLHE	Mitochondrion matrix {ECO:0000269|PubMed:15754339}.

Supplemental Table 1 Page 69. All proteins quantitated in study

Protein Name	Uniprot	Gene	Subcellular localization
Probable D-lactate dehydrogenase, mitochondrial	Q86WU2	LDHD	Mitochondrion {ECO:0000250|UniProtKB:Q7TNG8}.
Histone H2A.V	Q71UI9	H2AFV	Nucleus {ECO:0000250}. Chromosome {ECO:0000250}.
Dihydrolipoyl dehydrogenase, mitochondrial	P09622	DLD	Mitochondrion matrix {ECO:0000305|PubMed:29211711, ECO:0000305|PubMed:3693355}. Nucleus {ECO:0000269|PubMed:29211711}. Cell projection, cilium, flagellum {ECO:0000250|UniProtKB:Q811C4}. Cytoplasmic vesicle, secretory vesicle, acrosome {ECO:0000269|PubMed:15888450}. Note=Mainly localizes in the mitochondrion. A small fraction localizes to the nucleus, where the 2-oxoglutarate dehydrogenase complex is required for histone succinylation. {ECO:0000269|PubMed:29211711}.
NADH dehydrogenase [ubiquinone] 1 alpha subcomplex subunit 2	O43678	NDUFA2	Mitochondrion inner membrane {ECO:0000305|PubMed:12611891}; Peripheral membrane protein {ECO:0000305}; Matrix side {ECO:0000305}.
Diablo homolog, mitochondrial	Q9NR28	DIABLO	Mitochondrion {ECO:0000269|PubMed:14523016}. Note=Released into the cytosol when cells undergo apoptosis.
Elongation factor 1-gamma	P26641	EEF1G	0
Actin, alpha skeletal muscle	P68133	ACTA1	Cytoplasm, cytoskeleton.
Branched-chain-amino-acid aminotransferase, mitochondrial	O15382	BCAT2	Isoform A: Mitochondrion.; Isoform B: Cytoplasm.
Propionyl-CoA carboxylase alpha chain, mitochondrial	P05165	PCCA	Mitochondrion matrix {ECO:0000269|PubMed:16023992}.
Carnitine O-palmitoyltransferase 1, muscle isoform	Q92523	CPT1B	Mitochondrion outer membrane; Multi-pass membrane protein.
NADH dehydrogenase [ubiquinone] 1 beta subcomplex subunit 8, mitochondrial	O95169	NDUFB8	Mitochondrion inner membrane {ECO:0000305|PubMed:12611891}; Single-pass membrane protein {ECO:0000255}; Matrix side {ECO:0000305}.
Succinate--CoA ligase [GDP-forming] subunit beta, mitochondrial	Q96I99	SUCLG2	Mitochondrion {ECO:0000255|HAMAP-Rule:MF_03221}.
3-ketoacyl-CoA thiolase, mitochondrial	P42765	ACAA2	Mitochondrion {ECO:0000269|PubMed:18371312}. Note=Colocalizes with BNIP3 in the mitochondria.
Filamin-A	P21333	FLNA	Cytoplasm, cell cortex. Cytoplasm, cytoskeleton.

Supplemental Table 1 Page 70. All proteins quantitated in study

Protein Name	Uniprot	Gene	Subcellular localization
Clusterin	P10909	CLU	Isoform 1: Secreted {ECO:0000269|PubMed:11123922, ECO:0000269|PubMed:17260971, ECO:0000269|PubMed:17412999, ECO:0000269|PubMed:17451556, ECO:0000269|PubMed:2387851, ECO:0000269|PubMed:24073260, ECO:0000269|PubMed:2780565, ECO:0000269|PubMed:3154963, ECO:0000269|PubMed:8292612, ECO:0000269|PubMed:8328966}. Note=Can retrotranslocate from the secretory compartments to the cytosol upon cellular stress. {ECO:0000269|PubMed:17451556}.; Isoform 4: Cytoplasm {ECO:0000269|PubMed:24073260}. Note=Keeps cytoplasmic localization in stressed and unstressed cell. {ECO:0000269|PubMed:24073260}.; Isoform 6: Cytoplasm {ECO:0000269|PubMed:24073260}. Note=Keeps cytoplasmic localization in stressed and unstressed cell. {ECO:0000269|PubMed:24073260}.; Nucleus {ECO:0000269|PubMed:12551933, ECO:0000269|PubMed:19137541}. Cytoplasm {ECO:0000269|PubMed:12551933, ECO:0000269|PubMed:17689225, ECO:0000269|PubMed:19137541, ECO:0000269|PubMed:20068069, ECO:0000269|PubMed:22689054, ECO:0000269|PubMed:24073260}. Mitochondrion membrane; Peripheral membrane protein; Cytoplasmic side {ECO:0000269|PubMed:17689225}. Cytoplasm, cytosol {ECO:0000269|PubMed:17451556, ECO:0000269|PubMed:22689054, ECO:0000269|PubMed:24073260}. Microsome {ECO:0000269|PubMed:22689054}. Endoplasmic reticulum {ECO:0000269|PubMed:16113678, ECO:0000269|PubMed:22689054}. Mitochondrion {ECO:0000269|PubMed:16113678, ECO:0000269|PubMed:22689054}. Mitochondrion membrane {ECO:0000269|PubMed:16113678, ECO:0000269|PubMed:17689225}. Cytoplasm, perinuclear region {ECO:0000250|UniProtKB:P05371}. Cytoplasmic vesicle, secretory vesicle, chromaffin granule {ECO:0000250}. Note=Secreted isoforms can retrotranslocate from the secretory compartments to the cytosol upon cellular stress (PubMed:17451556). Detected in perinuclear foci that may be aggresomes containing misfolded, ubiquitinated proteins (PubMed:20068069). Detected at the mitochondrion membrane upon induction of apoptosis (PubMed:17689225). Under ER stress, a immaturely glycosylated pre-secreted form retrotranslocates from the endoplasmic reticulum (ER)-Golgi network to the cytoplasm to localize in the mitochondria through HSPA5 interaction (PubMed:22689054). ER stress reduces secretion (PubMed:22689054). Under the stress, minor amounts of non-secreted forms accumulate in cytoplasm (PubMed:24073260, PubMed:22689054, PubMed:17451556). Non-secreted forms emerge mainly from failed translocation, alternative splicing or non-canonical initiation start codon (PubMed:24073260, PubMed:12551933). {ECO:0000269|PubMed:12551933, ECO:0000269|PubMed:17451556, ECO:0000269|PubMed:17689225, ECO:0000269|PubMed:20068069, ECO:0000269|PubMed:22689054, ECO:0000269|PubMed:24073260}.

Supplemental Table 1 Page 71. All proteins quantitated in study

Protein Name	Uniprot	Gene	Subcellular localization
Dystroglycan	Q14118	DAG1	Alpha-dystroglycan: Secreted, extracellular space.; Beta-dystroglycan: Cell membrane; Single-pass type I membrane protein. Cytoplasm, cytoskeleton. Nucleus, nucleoplasm. Cell membrane, sarcolemma {ECO:0000250}. Cell junction, synapse, postsynaptic cell membrane {ECO:0000250}. Note=The monomeric form translocates to the nucleus via the action of importins and depends on RAN. Nuclear transport is inhibited by Tyr-892 phosphorylation. In skeletal muscle, this phosphorylated form locates to a vesicular internal membrane compartment. In muscle cells, sarcolemma localization requires the presence of ANK2, while localization to costameres requires the presence of ANK3. Localizes to neuromuscular junctions (NMJs) in the presence of ANK2 (By similarity). In peripheral nerves, localizes to the Schwann cell membrane. Colocalizes with ERM proteins in Schwann-cell microvilli. {ECO:0000250}.
40S ribosomal protein S7	P62081	RPS7	Cytoplasm, cytoskeleton, microtubule organizing center, centrosome {ECO:0000269|PubMed:14654843, ECO:0000269|PubMed:20873783}. Note=Colocalizes with NEK6 in the centrosome. {ECO:0000269|PubMed:20873783}.
Mannose-P-dolichol utilization defect 1 protein	O75352	MPDU1	Membrane {ECO:0000305}; Multi-pass membrane protein {ECO:0000305}.
Myeloperoxidase	P05164	MPO	Lysosome.

Supplemental Table 2. Differentially expressed proteins from heatmap separated by cluster (p<0.05, fold change > 3-fold).

-80		-83		-81		-67		-77		no cluster	
**Gene**	**Uniprot**	**Gene**	**Uniprot**	**Gene**	**Uniprot**	**Gene**	**Uniprot**	**Gene**	**Uniprot**	**Gene**	**Uniprot**
CMA1	P23946	TUBB	P07437	CARS2	Q9HA77	BCKDHA	P12694	HSPB6	O14558	RALA	P11233
CPA3	P15088	BSG	P35613	MRPS35	P82673	S100A1	P23297	FECH	P22830	PRXL2A	Q9BRX8
LDHB	P07195	TUBA4A	P68366	ECHDC2	Q86YB7	MRPL49	Q13405	PGM1	P36871	MYL6B	P14649
SHMT2	P34897	BIN1	O00499	OXSM	Q9NWU1	MAIP1	Q8WWC4				
ATP1A1	P05023	HSPA8	P11142	TMOD4	Q9NZQ9						
TPP1	O14773	PRKACA	P17612	LDB3	O75112						
APOA1	P02647	HSP90AA1	P07900	FKBP3	Q00688						
COL1A2	P08123	PPP2R1A	P30153								
ACAA1	P09110	PRDX4	Q13162								
MPC2	O95563	COPS7A	Q9UBW8								
MCU	Q8NE86	PDLIM3	Q53GG5								
TAP1	Q03518	HSPA1A	P0DMV8								
HLA-A	P01892	CAPNS1	P04632								
HLA-B	Q04826	KPNA4	O00629								
HLA-B	Q95365	CCT2	P78371								
HLA-H	P01893	EIF3F	O00303								
HLA-A	P05534	EIF4A1	P60842								
HLA-B	P30487	PRKAG1	P54619								
HLA-B	P30479	TUBB4B	P68371								
HLA-A	P04439	UNC45B	Q8IWX7								
HLA-C	P30508	ART3	Q13508								
HLA-DRB1	P04229	LRRC47	Q8N1G4								
HLA-B	P30460	DDX1	Q92499								
HLA-DRA	P01903	SAR1B	Q9Y6B6								
B2M	P61769	FHOD1	Q9Y613								
FGG	P02679	KPNB1	Q14974								
C3	P01024	VAPB	O95292								
ANXA1	P04083	PARVB	Q9HBI1								
Immunoglobulin gamma-1 heavy chain	P0DOX5	BZW2	Q9Y6E2								
Immunoglobulin kappa light chain	P0DOX7	MYOZ2	Q9NPC6								
GGT5	P36269	HSPA2	P54652								
ACTN4	O43707	PLIN4	Q96Q06								
HLA-A	P30443	HNRNPK	P61978								
HLA-B	P01889	KLHL41	O60662								
COL4A1	P02462	MYOT	Q9UBF9								
COL6A2	P12110	PLEC	Q15149								
MYH9	P35579	DES	P17661								

Supplemental Table 3 Page 1. Differentially expressed proteins using the broader criteria of log fold change > 1.5-fold (fold change > 2.83).

Gene	Uniprot	log2 fc	p-value	Known dysregulation in IIM
A8MU46	SMTNL1	-4.0	0.110332757	No
B0YJ81	HACD1	-2.4	0.529825676	No
O00159	MYO1C	1.6	0.022892984	No
O00186	STXBP3	-3.0	0.199438589	No
O00303	EIF3F	-3.2	0.049268968	No
O00410	IPO5	-2.3	0.103927534	No
O00429	DNM1L	-3.9	0.119716348	Yes
O00499	BIN1	-3.1	0.014403684	No
O00505	KPNA3	-2.3	0.237298829	No
O00629	KPNA4	-2.7	0.004549505	Yes
O00757	FBP2	-5.1	0.076525315	No
O14548	COX7A2L	2.5	0.047600486	No
O14558	HSPB6	-2.5	0.001114992	No
O14732	IMPA2	-3.1	0.110393885	No
O14773	TPP1	2.7	0.006169777	No
O15061	SYNM	-2.5	0.169150061	No
O43615	TIMM44	1.7	0.030451795	No
O43674	NDUFB5	-2.5	0.55814032	No
O43676	NDUFB3	-3.2	0.646221363	No
O43681	ASNA1	-2.8	0.124546765	No
O43707	ACTN4	2.4	0.008497462	No
O43819	SCO2	-2.4	0.368418507	No
O60662	KLHL41	-2.0	0.046842744	No
O60664	PLIN3	-4.2	0.137325454	No
O60763	USO1	-2.2	0.103090829	No
O75112	LDB3	-4.1	2.37334E-05	Yes
O75128	COBL	-1.6	0.385041151	No
O75155	CAND2	-2.6	0.10645561	No
O94826	TOMM70	-2.0	0.078405329	No
O95139	NDUFB6	-2.1	0.051415212	No
O95210	STBD1	-3.6	0.059585958	No
O95292	VAPB	-3.0	0.032663565	No
O95479	H6PD	-2.3	0.167493417	No
O95563	MPC2	2.2	0.003215939	No
O95674	CDS2	-1.7	0.60471683	No
P00918	CA2	1.7	0.07902955	No
P01024	C3	2.3	0.003413826	Yes
P01876	IGHA1	2.2	0.030254584	No
P01892	HLA-A	2.9	0.000666857	Yes
P01893	HLA-H	4.0	4.63886E-05	Yes
P01903	HLA-DRA	6.3	0.007047072	Yes
P02452	COL1A1	3.9	0.065427372	No

Supplemental Table 3 Page 2. Differentially expressed proteins using the broader criteria of log fold change > 1.5-fold (fold change > 2.83).

Gene	Uniprot	log2 fc	p-value	Known dysregulation in IIM
P02461	COL3A1	2.9	0.047099047	No
P02462	COL4A1	2.8	0.032135937	No
P02545	LMNA	-4.9	0.057328831	Yes
P02585	TNNC2	-6.6	0.05288353	No
P02647	APOA1	2.4	0.003212939	Yes
P02675	FGB	1.8	0.055195803	Yes
P02679	FGG	3.4	0.024774286	Yes
P02724	GYPA	-3.8	0.217449886	No
P03891	MT-ND2	-2.4	0.271892303	No
P04083	ANXA1	1.9	0.017857248	Yes
P04217	A1BG	3.4	0.033000545	No
P04229	HLA-DRB1	4.4	0.002128458	Yes
P04632	CAPNS1	-3.8	0.021361808	No
P04899	GNAI2	1.7	0.020138201	No
P05023	ATP1A1	2.2	0.039251248	No
P05386	RPLP1	-1.5	0.923403269	No
P05387	RPLP2	-4.2	0.571664666	No
P07195	LDHB	1.5	0.009917201	No
P07437	TUBB	-1.7	0.00374033	No
P07585	DCN	1.5	0.064394588	No
P07900	HSP90AA1	-1.7	0.042759368	No
P08123	COL1A2	2.8	0.020907725	No
P08237	PFKM	-1.5	0.035190235	No
P08670	VIM	-4.3	0.063017473	No
P09110	ACAA1	1.9	0.014039676	No
P09429	HMGB1	-3.1	0.682724408	Yes
P0DMV8	HSPA1A	-1.7	0.006011475	Yes
P0DOX7	IGKC	1.8	0.003926573	No
P11055	MYH3	2.3	0.1005255	No
P11142	HSPA8	-1.9	2.32817E-05	No
P11233	RALA	-3.6	0.033299778	No
P12109	COL6A1	2.3	0.029671758	Yes
P12110	COL6A2	1.9	0.046041013	No
P12694	BCKDHA	2.2	0.007839783	No
P13639	EEF2	-2.0	0.309572037	No
P13807	GYS1	-3.8	0.09616956	No
P13861	PRKAR2A	-5.2	0.07257047	No
P14314	PRKCSH	-2.2	0.284074797	No
P14649	MYL6B	2.0	0.01768471	No
P15088	CPA3	2.2	0.009761953	No
P15311	EZR	1.7	0.022947391	No

Supplemental Table 3 Page 3. Differentially expressed proteins using the broader criteria of log fold change > 1.5-fold (fold change > 2.83).

Gene	Uniprot	log2 fc	p-value	Known dysregulation in IIM
P15954	COX7C	-2.2	1.38599E-05	No
P16403	HIST1H1C	-1.9	0.343437748	No
P16989	YBX3	-4.0	0.137399545	No
P17661	DES	-2.6	0.013997002	No
P18206	VCL	-2.2	0.281973684	No
P19105	MYL12A	5.5	0.046173046	No
P19338	NCL	-1.8	0.667579647	No
P23946	CMA1	2.5	0.015226731	No
P26038	MSN	1.5	0.059437366	No
P30153	PPP2R1A	-2.6	0.014759111	No
P30508	HLA-C	5.3	0.00236009	Yes
P34897	SHMT2	3.7	0.034570236	No
P35573	AGL	-2.6	0.163155831	No
P35579	MYH9	2.2	0.029344361	No
P35613	BSG	-1.7	0.003381245	No
P36269	GGT5	2.2	0.008435173	No
P36871	PGM1	-1.7	9.1305E-06	No
P40123	CAP2	-3.3	0.051069721	No
P43897	TSFM	-2.3	0.348781219	No
P47897	QARS	-1.5	0.068227846	No
P48788	TNNI2	-5.7	0.073071929	No
P50336	PPOX	-1.5	0.152333791	No
P50579	METAP2	-2.8	0.10586647	No
P51149	RAB7A	-4.4	0.170797665	Yes
P51553	IDH3G	-2.9	0.29045966	No
P54619	PRKAG1	-4.2	0.047841139	No
P54652	HSPA2	-4.4	0.02877411	No
P55060	CSE1L	-2.1	0.102248102	No
P55290	CDH13	-2.8	0.082793228	No
P60842	EIF4A1	-3.6	0.037211148	No
P61019	RAB2A	-4.2	0.209408207	No
P61088	UBE2N	-3.7	0.134615443	No
P61769	B2M	5.9	0.00074605	Yes
P61978	HNRNPK	-3.3	0.052426279	No
P62070	RRAS2	-3.0	0.110872842	No
P62942	FKBP1A	-4.6	0.200638706	No
P68032	ACTC1	1.8	0.045434215	No
P68366	TUBA4A	-1.9	0.001271591	No
P68371	TUBB4B	-3.5	0.007894288	No
P78371	CCT2	-3.5	4.89118E-06	No
P82673	MRPS35	-3.0	0.035970959	No
P82933	MRPS9	-1.8	0.443850291	No

Supplemental Table 3 Page 4. Differentially expressed proteins using the broader criteria of log fold change > 1.5-fold (fold change > 2.83).

Gene	Uniprot	log2 fc	p-value	Known dysregulation in IIM
Q00688	FKBP3	-3.5	0.023600796	No
Q02161	RHD	2.1	0.025319125	No
Q02221	COX6A2	-4.5	0.334599498	No
Q02641	CACNB1	-4.3	0.064832485	No
Q02750	MAP2K1	-2.4	0.070836237	No
Q03252	LMNB2	-2.5	0.216188134	No
Q03518	TAP1	5.2	0.000880626	Yes
Q04826	HLA-B	4.1	0.000123047	Yes
Q06432	CACNG1	-3.2	0.307107644	No
Q08043	ACTN3	-2.2	0.235839727	Yes
Q13162	PRDX4	-2.5	0.027086199	No
Q13405	MRPL49	2.1	0.003199723	No
Q13424	SNTA1	-4.2	0.079134944	No
Q13425	SNTB2	-1.7	0.063679353	No
P21810	BGN	3.6	0.035730819	Yes
P21964	COMT	-1.8	0.187479561	No
P22061	PCMT1	-4.7	0.082583282	No
P22830	FECH	-2.3	0.014739743	No
P23109	AMPD1	-4.7	0.125239441	Yes
P23297	S100A1	3.7	0.000184071	No
P23327	HRC	-1.8	0.226632159	No
P23368	ME2	1.6	0.013008018	No
Q13508	ART3	-3.3	0.029593722	No
Q13554	CAMK2B	-3.7	0.187213621	No
Q13557	CAMK2D	-2.8	0.659340342	No
Q13586	STIM1	-1.5	0.170970366	No
Q13698	CACNA1S	-1.5	0.299346271	No
Q14165	MLEC	-2.7	0.605025057	No
Q14324	MYBPC2	-2.1	0.113212678	No
Q14974	KPNB1	-2.7	6.06202E-06	No
Q15006	EMC2	-2.0	0.554314968	No
Q15149	PLEC	-2.0	0.006290887	No
Q15365	PCBP1	-4.7	0.126117826	No
Q16851	UGP2	-2.9	0.127953396	No
Q2TBA0	KLHL40	-3.1	0.188191946	No
Q53GG5	PDLIM3	-5.6	0.009061839	No
Q5SV97	PERM1	-3.3	0.176454789	No
Q6IAN0	DHRS7B	-1.9	0.213416979	No
Q6PCB7	SLC27A1	-2.1	0.211144198	No
Q6QEF8	CORO6	-2.1	0.060782306	No
Q7Z434	MAVS	-3.0	0.283769218	Yes
Q86YB7	ECHDC2	-3.1	0.048635827	No

Supplemental Table 3 Page 5. Differentially expressed proteins using the broader criteria of log fold change > 1.5-fold (fold change > 2.83).

Gene	Uniprot	log2 fc	p-value	Known dysregulation in IIM
Q8IWW8	ADHFE1	-2.5	0.667469667	No
Q8IWX7	UNC45B	-3.6	0.002199043	Yes
Q8IXM3	MRPL41	1.5	0.117231499	No
Q8N142	ADSSL1	-1.5	5.81733E-05	No
Q8N1G4	LRRC47	-2.5	0.000175629	No
Q8N465	D2HGDH	-2.2	0.340975853	No
Q8N4Q0	ZADH2	-1.5	0.758921091	No
Q8N5K1	CISD2	5.1	0.011510716	No
Q8NB12	SMYD1	-3.1	0.20804061	No
Q8NE86	MCU	1.7	0.003239405	No
Q8TB37	NUBPL	-2.1	0.841369012	No
Q8TD30	GPT2	-2.0	0.288771877	No
Q8TDC0	MYOZ3	-4.5	0.30404495	No
Q8WUM4	PDCD6IP	-2.0	0.157142915	No
Q8WWC4	MAIP1	4.1	0.001503921	No
Q8WWV3	RTN4IP1	-1.5	0.854047671	No
Q92499	DDX1	-2.0	0.005716228	No
Q96A26	FAM162A	-3.5	0.148420518	No
Q96BQ5	CCDC127	-3.1	0.379752133	No
Q96EY1	DNAJA3	-2.3	0.089756144	No
Q96HC4	PDLIM5	-4.0	0.168123525	No
Q96Q06	PLIN4	-5.2	0.01580523	No
Q9BR39	JPH2	-4.2	0.17766303	No
Q9BRX8	PRXL2A	1.7	0.024702621	No
Q9BT78	COPS4	-3.0	0.239503881	No
Q9BWM7	SFXN3	3.5	0.029193685	No
Q9BX68	HINT2	-3.3	0.956139796	No
Q9BXI3	NT5C1A	-2.4	0.902769609	Yes
Q9BXK5	BCL2L13	-3.7	0.107263944	No
Q9BZE1	MRPL37	-2.3	0.335748258	No
Q9H1R3	MYLK2	-4.1	0.110999691	No
Q9H3N1	TMX1	-2.3	0.100131082	No
Q9H987	SYNPO2L	-2.1	0.089540587	No
Q9HA77	CARS2	-1.8	0.004268332	No
Q9HBI1	PARVB	-3.3	0.00061543	No
Q9HCU5	PREB	-2.7	0.274492313	No
Q9HDC5	JPH1	-4.4	0.063868612	No
Q9NP72	RAB18	-2.9	0.105847238	No
Q9NP98	MYOZ1	-6.6	0.062037062	No
Q9NPC6	MYOZ2	-3.7	0.036817196	No
Q9NWU1	OXSM	-2.0	0.032848914	No

Supplemental Table 3 Page 6. Differentially expressed proteins using the broader criteria of log fold change > 1.5-fold (fold change > 2.83).

Gene	Uniprot	log2 fc	p-value	Known dysregulation in IIM
Q9Y6E2	BZW2	-3.8	0.032837967	No
Q9Y6H1	CHCHD2	-4.4	0.233974482	No
Q9Y6N5	SQOR	1.8	0.024479227	No
Q9NZN4	EHD2	-1.9	0.134662671	No
Q9NZQ9	TMOD4	-5.0	0.041083885	No
Q9NZU5	LMCD1	-4.8	0.112576741	No
Q9P0J1	PDP1	-1.6	0.103082553	No
Q9P0L0	VAPA	-3.8	0.098939163	No
Q9UBF9	MYOT	-4.7	0.019899696	No
Q9UBW8	COPS7A	-4.3	0.001635387	No
Q9UL25	RAB21	-4.8	0.056158292	No
Q9UMS6	SYNPO2	-3.0	0.215698101	No
Q9UN36	NDRG2	-3.7	0.198342781	No
Q9UNS2	COPS3	-2.4	0.095398394	No
Q9UQM7	CAMK2A	-1.6	0.443784634	No
Q9Y235	APOBEC2	-2.5	0.066459185	No
Q9Y2Q3	GSTK1	1.6	0.00877361	No
Q9Y5U8	MPC1	1.5	0.017911838	Yes
Q9Y613	FHOD1	-2.6	0.042156051	No
Q9Y6B6	SAR1B	-3.3	0.04539695	No

Supplemental Table 4 Page 1. Significantly underrepresented cellular pathways in IIM

Pathway name	*p* value	FDR	Submitted entities found
Cellular response to stress			
HSF1-dependent transactivation	2.84E-12	2.28E-09	CAMK2B; HSPA8; CAMK2D; HSP90AA1; CAMK2A; HSPA2; HSPA1A
Attenuation phase	1.35E-09	5.42E-07	HSPA8; HSP90AA1; HSPA2; HSPA1A
Cellular response to heat stress	5.49E-08	1.10E-05	CAMK2B; HSPA8; CAMK2D; HSP90AA1; CAMK2A; HSPA2; HSPA1A
HSP90 chaperone cycle for steroid hormone receptors (SHR) in the presence of ligand	7.20E-08	1.15E-05	HSPA8; HSP90AA1; TUBB; HSPA2; TUBB4B; TUBA4A; HSPA1A; CAP2
Regulation of HSF1-mediated heat shock response	2.80E-05	0.0014	HSPA8; HSPA2; HSPA1A
HSF1 activation	3.06E-05	0.0014	HSP90AA1; HSPA1A
Cellular responses to stress	9.06E-05	0.0036	CAMK2B; HSPA8; CAMK2D; HSP90AA1; TUBB; RPLP1; CAMK2A; HSPA2; TUBB4B; COX7C; TUBA4A; VAPA; SCO2; LMNA; RPLP2; PREB; HSPA1A; CAP2; HIST1H1C
Cellular responses to stimuli	1.23E-04	0.0042	CAMK2B; HSPA8; CAMK2D; HSP90AA1; TUBB; RPLP1; CAMK2A; HSPA2; TUBB4B; COX7C; TUBA4A; VAPA; SCO2; LMNA; RPLP2; PREB; HSPA1A; CAP2; HIST1H1C
Autophagy			
Selective autophagy	5.09E-08	1.10E-05	TOMM70; HSPA8; HSP90AA1; TUBB; PLIN4; UBE2N; PRKAG1; PLIN3; VIM; TUBB4B; TUBA4A
Aggrephagy	4.35E-06	4.35E-04	HSP90AA1; TUBB; UBE2N; VIM; TUBB4B; TUBA4A
Macroautophagy	7.78E-06	5.45E-04	TOMM70; HSPA8; HSP90AA1; TUBB; PLIN4; UBE2N; PRKAG1; PLIN3; VIM; TUBB4B; TUBA4A
Autophagy	1.97E-05	0.0012	TOMM70; HSPA8; HSP90AA1; TUBB; PLIN4; UBE2N; PRKAG1; PLIN3; VIM; TUBB4B; TUBA4A
Chaperone Mediated Autophagy	2.96E-04	0.0080	HSPA8; HSP90AA1; PLIN3; VIM
Programmed cell death			
Apoptotic execution phase	9.28E-07	1.23E-04	LMNA; VIM; HMGB1; DNM1L; KPNB1; PLEC; HIST1H1C
Apoptosis induced DNA fragmentation	3.35E-05	0.0014	HMGB1; KPNB1; HIST1H1C
Programmed Cell Death	0.0016	0.021	HSP90AA1; PDCD6IP; LMNA; VIM; HMGB1; DNM1L; KPNB1; PLEC; HIST1H1C
Apoptosis	0.0049	0.044	LMNA; VIM; HMGB1; DNM1L; KPNB1; PLEC; HIST1H1C

Supplemental Table 4 Page 2. Significantly underrepresented cellular pathways in IIM

Pathway name	*p* value	FDR	Submitted entities found
Muscle contraction			
Striated Muscle Contraction	1.52E-06	1.74E-04	MYBPC2; DES; ACTN3; TMOD4; TNNC2; TNNI2; VIM
Muscle contraction	8.25E-06	5.45E-04	CAMK2B; CAMK2D; MYBPC2; ACTN3; TMOD4; CAMK2A; TNNC2; CACNB1; DES; STIM1; TNNI2; VIM; VCL
Phase 0 - rapid depolarisation	0.0013	0.018	CAMK2B; CACNB1; CAMK2D; CAMK2A
Synaptic communication			
Assembly and cell surface presentation of NMDA receptors	5.69E-06	5.07E-04	CAMK2B; CAMK2D; TUBB; CAMK2A; TUBB4B; TUBA4A
Post NMDA receptor activation events	7.85E-06	5.45E-04	CAMK2B; CAMK2D; TUBB; PRKAR2A; CAMK2A; PRKAG1; TUBB4B; TUBA4A
Activation of NMDA receptors and postsynaptic events	2.80E-05	0.0014	CAMK2B; CAMK2D; TUBB; PRKAR2A; CAMK2A; PRKAG1; TUBB4B; TUBA4A
Activation of AMPK downstream of NMDARs	1.12E-04	0.0041	TUBB; PRKAG1; TUBB4B; TUBA4A
Membrane trafficking			
Translocation of SLC2A4 (GLUT4) to the plasma membrane	1.34E-04	0.0044	RALA; STXBP3; TUBB; PRKAG1; TUBB4B; TUBA4A
COPI-independent Golgi-to-ER retrograde traffic	2.42E-04	0.0075	TUBB; RAB18; TUBB4B; TUBA4A; CAP2
Microtubule-dependent trafficking of connexons from Golgi to the plasma membrane	2.50E-04	0.0075	TUBB; TUBB4B; TUBA4A
Transport of connexons to the plasma membrane	2.96E-04	0.0080	TUBB; TUBB4B; TUBA4A
Membrane Trafficking	3.16E-04	0.0082	HSPA8; RALA; STXBP3; SAR1B; TUBB; USO1; PRKAG1; COPS7A; TUBB4B; TUBA4A; COPS4; RAB21; COPS3; BIN1; RAB18; PLIN4; PLIN3; PREB; CAP2; RAB7A
Anchoring of the basal body to the plasma membrane	0.0024	0.027	HSP90AA1; PPP2R1A; TUBB; TUBB4B; TUBA4A
MHC class II antigen presentation	0.0044	0.040	SAR1B; TUBB; TUBB4B; TUBA4A; CAP2; RAB7A
Citric acid cycle and respiratory electron transport			
The citric acid (TCA) cycle and respiratory electron transport	1.13E-04	0.0041	PDP1; NDUFB6; NDUFB5; IDH3G; NUBPL; SCO2; ADHFE1; NDUFB3; BSG; D2HGDH; MT-ND2; COX7C
Respiratory electron transport	0.0012	0.017	NDUFB6; NDUFB5; NUBPL; SCO2; NDUFB3; MT-ND2; COX7C
Complex I biogenesis	0.0013	0.019	NDUFB6; NDUFB5; NUBPL; NDUFB3; MT-ND2
Respiratory electron transport, ATP synthesis by chemiosmotic coupling, and heat production by uncoupling proteins.	0.0051	0.046	NDUFB6; NDUFB5; NUBPL; SCO2; NDUFB3; MT-ND2; COX7C

Supplemental Table 4 Page 3. Significantly underrepresented cellular pathways in IIM

Pathway name	*p* value	FDR	Submitted entities found
Cell cycle, mitotic			
Nuclear Envelope (NE) Reassembly	3.13E-05	0.0014	PPP2R1A; TUBB; LMNA; TUBB4B; TUBA4A; KPNB1
Recruitment of NuMA to mitotic centrosomes	3.94E-04	0.010	HSP90AA1; PPP2R1A; TUBB; TUBB4B; TUBA4A
Loss of proteins required for interphase microtubule organization from the centrosome	4.54E-04	0.011	HSP90AA1; PPP2R1A; TUBB; TUBB4B; TUBA4A
Loss of Nlp from mitotic centrosomes	4.54E-04	0.011	HSP90AA1; PPP2R1A; TUBB; TUBB4B; TUBA4A
Initiation of Nuclear Envelope (NE) Reformation	5.38E-04	0.012	PPP2R1A; LMNA; KPNB1
AURKA Activation by TPX2	5.63E-04	0.012	HSP90AA1; PPP2R1A; TUBB; TUBB4B; TUBA4A
Recruitment of mitotic centrosome proteins and complexes	8.96E-04	0.015	HSP90AA1; PPP2R1A; TUBB; TUBB4B; TUBA4A
Centrosome maturation	0.00101471	0.015	HSP90AA1; PPP2R1A; TUBB; TUBB4B; TUBA4A
Regulation of PLK1 Activity at G2/M Transition	0.0017	0.022	HSP90AA1; PPP2R1A; TUBB; TUBB4B; TUBA4A
Sealing of the nuclear envelope (NE) by ESCRT-III	0.0021	0.025	TUBB; TUBB4B; TUBA4A
The role of GTSE1 in G2/M progression after G2 checkpoint	0.0057	0.046	HSP90AA1; TUBB; TUBB4B; TUBA4A
Oncogenic MAPK signaling			
RAF activation	2.64E-04	0.0077	CAMK2B; MAP2K1; CAMK2D; PPP2R1A; CAMK2A
Signaling by BRAF and RAF1 fusions	5.25E-04	0.012	CAMK2B; MAP2K1; CAMK2D; CAMK2A; LMNA; VCL
Signaling by RAF1 mutants	5.90E-04	0.012	CAMK2B; MAP2K1; CAMK2D; CAMK2A; VCL
Signaling downstream of RAS mutants	9.09E-04	0.015	CAMK2B; MAP2K1; CAMK2D; CAMK2A; VCL
Signaling by moderate kinase activity BRAF mutants	9.09E-04	0.015	CAMK2B; MAP2K1; CAMK2D; CAMK2A; VCL
Paradoxical activation of RAF signaling by kinase inactive BRAF	9.09E-04	0.015	CAMK2B; MAP2K1; CAMK2D; CAMK2A; VCL
Signaling by RAS mutants	9.09E-04	0.015	CAMK2B; MAP2K1; CAMK2D; CAMK2A; VCL
Oncogenic MAPK signaling	0.0018	0.022	CAMK2B; MAP2K1; CAMK2D; CAMK2A; LMNA; VCL
Diseases of programmed cell death			
Neurodegenerative Diseases	7.95E-04	0.014	PRDX4; CAPNS1; LMNA
Deregulated CDK5 triggers multiple neurodegenerative pathways in Alzheimer's disease models	7.95E-04	0.014	PRDX4; CAPNS1; LMNA
Defective Intrinsic Pathway for Apoptosis	0.00112805	0.017	PRDX4; CAPNS1; LMNA

Supplemental Table 4 Page 4. Significantly underrepresented cellular pathways in IIM

Pathway name	*p* value	FDR	Submitted entities found
Protein folding			
Prefoldin mediated transfer of substrate to CCT/TriC	7.02E-04	0.014	CCT2; TUBB; TUBB4B; TUBA4A
Formation of tubulin folding intermediates by CCT/TriC	7.95E-04	0.014	CCT2; TUBB; TUBB4B; TUBA4A
Cooperation of Prefoldin and TriC/CCT in actin and tubulin folding	0.0017	0.022	CCT2; TUBB; TUBB4B; TUBA4A
Post-chaperonin tubulin folding pathway	0.0049	0.044	TUBB; TUBB4B; TUBA4A
Metabolism of proteins			
Translation	7.95E-04	0.014	EIF4A1; TSFM; MRPS9; MRPS35; QARS; RPLP1; RPLP2; EIF3F; MRPL37; EEF2; CARS2
Protein methylation	0.0030	0.030	HSPA8; EEF2; ART3
Metabolism of proteins	0.0050	0.045	EIF4A1; MRPS35; SAR1B; PRKCSH; RPLP1; USO1; COPS7A; MRPL37; ART3; RAB21; PCMT1; MLEC; RPLP2; LMCD1; CAP2; RAB2A; TOMM70; HSPA8; CCT2; TSFM; TUBB; GPT2; TUBB4B; EEF2; TUBA4A; KLHL41; COPS4; COPS3; MRPS9; MAVS; HNRNPK; QARS; HRC; RAB18; UBE2N; PREB; EIF3F; CARS2; RAB7A
Alanine metabolism	0.0031	0.031	GPT2
Calcium-dependent events			
CaMK IV-mediated phosphorylation of CREB	7.81E-04	0.014	CAMK2B; CAMK2D; CAMK2A
Calmodulin induced events	0.0029	0.029	CAMK2B; CAMK2D; PRKAR2A; CAMK2A
CaM pathway	0.0029	0.029	CAMK2B; CAMK2D; PRKAR2A; CAMK2A
Ca-dependent events	0.0043	0.040	CAMK2B; CAMK2D; PRKAR2A; CAMK2A
DAG and IP3 signaling	0.0061	0.049	CAMK2B; CAMK2D; PRKAR2A; CAMK2A
Signaling by Rho GTPases, Miro GTPases and RHOBTB3			
RHOD GTPase cycle	0.00115279	0.017	HINT2; VAPB; STBD1; CAP2; RAB7A
RHOBTB GTPase Cycle	0.00154837	0.020	COPS4; CCT2; HSP90AA1; VIM
RHO GTPases activate IQGAPs	0.00154837	0.020	TUBB; TUBB4B; TUBA4A
RHOBTB1 GTPase cycle	0.0044	0.040	COPS4; CCT2; VIM

Supplemental Table 4 Page 5. Significantly underrepresented cellular pathways in IIM

Pathway name	*p* value	FDR	Submitted entities found
Vesicle-mediated transport			
ER to Golgi Anterograde Transport	0.0019	0.023	SAR1B; TUBB; USO1; PREB; TUBB4B; TUBA4A; CAP2
Vesicle-mediated transport	0.0020	0.024	HSPA8; RALA; HSP90AA1; STXBP3; SAR1B; TUBB; USO1; PRKAG1; COPS7A; TUBB4B; TUBA4A; COPS4; RAB21; COPS3; BIN1; RAB18; PLIN4; PLIN3; PREB; CAP2; RAB7A
Gap junction assembly	0.0025	0.027	TUBB; TUBB4B; TUBA4A
COPI-mediated anterograde transport	0.0036	0.036	TUBB; USO1; TUBB4B; TUBA4A; CAP2
Gap junction trafficking	0.0057	0.046	TUBB; TUBB4B; TUBA4A
Transmission across chemical synapses			
Transmission across Chemical Synapses	0.0027	0.029	CAMK2B; CACNB1; HSPA8; CAMK2D; TUBB; PRKAR2A; CAMK2A; PRKAG1; TUBB4B; COMT; TUBA4A
Neurotransmitter receptors and postsynaptic signal transmission	0.0046	0.042	CAMK2B; CAMK2D; TUBB; PRKAR2A; CAMK2A; PRKAG1; TUBB4B; TUBA4A
Unblocking of NMDA receptors, glutamate binding and activation	0.0061	0.049	CAMK2B; CAMK2D; CAMK2A
Ras activation upon Ca2+ influx through NMDA receptor	0.0061	0.049	CAMK2B; CAMK2D; CAMK2A
Negative regulation of NMDA receptor-mediated neuronal transmission	0.0061	0.049	CAMK2B; CAMK2D; CAMK2A
Metabolism of carbohydrates			
Glycogen metabolism	0.0029	0.029	GYS1; UGP2; AGL; PGM1
Glycogen synthesis	0.0055	0.046	GYS1; UGP2; PGM1
Other			
Neutrophil degranulation	3.32E-05	0.0014	HSPA8; CCT2; HSP90AA1; TUBB; AGL; HMGB1; TUBB4B; EEF2; PRDX4; VAPA; STBD1; RAB18; MLEC; PGM1; VCL; KPNB1; HSPA1A; RAB7A
Lipophagy	6.21E-04	0.013	HSPA8; PLIN4; PRKAG1; PLIN3
DNA Damage Recognition in GG-NER	0.0023	0.025	COPS4; COPS3; COPS7A
Factors involved in megakaryocyte development and platelet production	0.0052	0.046	EHD2; TUBB; PRKAR2A; TUBB4B; TUBA4A; CAP2
ISG15 antiviral mechanism	0.0057	0.046	EIF4A1; UBE2N; KPNA4; KPNA3; KPNB1
Influenza Infection	0.0059	0.047	HSP90AA1; RPLP1; RPLP2; KPNA4; KPNA3; KPNB1; HSPA1A; IPO5

Supplemental Table 5 Page 1. Significantly overrepresented cellular pathways in IIM

Pathway name	*p* value	FDR	Submitted entities found
Adaptive immune system			
Translocation of ZAP-70 to Immunological synapse	1.11E-16	1.78E-15	HLA-DRA; HLA-DRB1
Antigen Presentation: Folding, assembly and peptide loading of class I MHC	1.11E-16	1.78E-15	HLA-H; HLA-B; HLA-C; TAP1; HLA-A; B2M
ER-Phagosome pathway	1.11E-16	1.78E-15	FGB; HLA-H; S100A1; FGG; HLA-B; HLA-C; TAP1; HLA-A; B2M
Endosomal/Vacuolar pathway	1.11E-16	1.78E-15	HLA-H; HLA-B; HLA-C; HLA-A; B2M
Phosphorylation of CD3 and TCR zeta chains	1.11E-16	1.78E-15	HLA-DRA; HLA-DRB1
Class I MHC mediated antigen processing & presentation	1.11E-16	1.78E-15	FGB; HLA-H; S100A1; FGG; HLA-B; HLA-C; TAP1; HLA-A; B2M
Antigen processing-Cross presentation	1.11E-16	1.78E-15	FGB; HLA-H; S100A1; FGG; HLA-B; HLA-C; TAP1; HLA-A; B2M
Immunoregulatory interactions between a Lymphoid and a non-Lymphoid cell	1.11E-16	1.78E-15	COL1A1; C3; HLA-H; COL3A1; COL1A2; HLA-B; HLA-C; HLA-A; B2M
Adaptive Immune System	1.11E-16	1.78E-15	FGB; HLA-H; S100A1; FGG; HLA-B; HLA-C; TAP1; HLA-A; COL1A1; C3; COL3A1; COL1A2; HLA-DRA; B2M; HLA-DRB1
PD-1 signaling	1.11E-16	1.78E-15	HLA-DRA; HLA-DRB1
Interferon gamma signaling	1.11E-16	1.78E-15	HLA-H; HLA-B; HLA-C; HLA-DRA; HLA-A; B2M; HLA-DRB1
Interferon alpha/beta signaling	1.11E-16	1.78E-15	HLA-H; HLA-B; HLA-C; HLA-A
Generation of second messenger molecules	6.22E-15	9.95E-14	HLA-DRA; HLA-DRB1
Costimulation by the CD28 family	6.02E-12	9.03E-11	HLA-DRA; HLA-DRB1
Downstream TCR signaling	1.12E-10	1.57E-09	HLA-DRA; HLA-DRB1
TCR signaling	9.82E-10	1.38E-08	HLA-DRA; HLA-DRB1
MHC class II antigen presentation	1.17E-09	1.51E-08	HLA-DRA; HLA-DRB1
Nef mediated downregulation of MHC class I complex cell surface expression	0.0064	0.0384	HLA-A; B2M
Cytokine signaling in immune system			
Interferon Signaling	1.11E-16	1.78E-15	HLA-H; HLA-B; HLA-C; HLA-DRA; HLA-A; B2M; HLA-DRB1
Cytokine Signaling in Immune system	1.11E-16	1.78E-15	HLA-H; ANXA1; COL1A2; CA2; HLA-B; HLA-C; MSN; HLA-DRA; HLA-A; B2M; HLA-DRB1

Supplemental Table 5 Page 2. Significantly overrepresented cellular pathways in IIM

Pathway name	*p* value	FDR	Submitted entities found
Infectious Disease			
SARS-CoV-2 activates/modulates innate and adaptive immune responses	1.11E-16	1.78E-15	HLA-H; HLA-B; HLA-C; HLA-A; B2M
SARS-CoV-2-host interactions	1.11E-16	1.78E-15	HLA-H; HLA-B; HLA-C; HLA-A; B2M
Disease	1.11E-16	1.78E-15	FGB; GGT5; HLA-H; S100A1; FGG; HLA-B; BGN; HLA-C; APOA1; MSN; HLA-A; ATP1A1; DCN; GNAI2; C3; MYO1C; MYH9; B2M
Infectious disease	1.11E-16	1.78E-15	C3; GGT5; HLA-H; MYO1C; HLA-B; MYH9; HLA-C; HLA-A; ATP1A1; B2M; GNAI2
SARS-CoV-2 Infection	1.11E-16	1.78E-15	HLA-H; HLA-B; HLA-C; HLA-A; B2M
SARS-CoV Infections	1.11E-16	1.78E-15	HLA-H; HLA-B; HLA-C; HLA-A; ATP1A1; B2M
Extracellular matrix organization			
ECM proteoglycans	2.71E-06	3.52E-05	COL1A1; COL3A1; COL1A2; COL4A1; COL6A2; COL6A1; BGN; DCN
Integrin cell surface interactions	5.01E-06	6.01E-05	COL1A1; FGB; COL3A1; COL1A2; COL4A1; COL6A2; COL6A1; FGG
Collagen chain trimerization	9.47E-06	1.14E-04	COL1A1; COL3A1; COL1A2; COL4A1; COL6A2; COL6A1
Assembly of collagen fibrils and other multimeric structures	9.65E-05	0.0011	COL1A1; COL3A1; COL1A2; COL4A1; COL6A2; COL6A1
Collagen degradation	1.13E-04	0.0012	COL1A1; COL3A1; COL1A2; COL4A1; COL6A2; COL6A1
Collagen biosynthesis and modifying enzymes	1.90E-04	0.0021	COL1A1; COL3A1; COL1A2; COL4A1; COL6A2; COL6A1
Degradation of the extracellular matrix	2.21E-04	0.0022	COL1A1; COL3A1; COL1A2; CMA1; COL4A1; COL6A2; COL6A1; DCN
Anchoring fibril formation	6.05E-04	0.0061	COL1A1; COL1A2; COL4A1
Extracellular matrix organization	9.24E-04	0.0083	COL1A1; FGB; COL3A1; COL1A2; CMA1; COL4A1; COL6A2; COL6A1; FGG; BGN; DCN
Collagen formation	9.77E-04	0.0088	COL1A1; COL3A1; COL1A2; COL4A1; COL6A2; COL6A1
Crosslinking of collagen fibrils	0.0023	0.0185	COL1A1; COL1A2; COL4A1
Syndecan interactions	0.0039	0.0275	COL1A1; COL3A1; COL1A2
Non-integrin membrane-ECM interactions	0.0044	0.0311	COL1A1; COL3A1; COL1A2; COL4A1
Vesicle-mediated transport			
Scavenging by Class A Receptors	2.06E-04	0.0021	COL1A1; COL3A1; COL1A2; COL4A1; APOA1
Binding and Uptake of Ligands by Scavenger Receptors	0.0100	0.0498	COL1A1; COL3A1; COL1A2; COL4A1; APOA1; IGHA1
Metabolism			
Reversible hydration of carbon dioxide	8.68E-04	0.0078	CA2
Rhesus blood group biosynthesis	8.89E-04	0.0080	RHD
Dermatan sulfate biosynthesis	0.0088	0.0473	BGN; DCN

Supplemental Table 5 Page 3. Significantly overrepresented cellular pathways in IIM

Pathway name	*p* value	FDR	Submitted entities found
Nervous system development			
NCAM1 interactions	0.0014	0.0110	COL3A1; COL4A1; COL6A2; COL6A1
NCAM signaling for neurite out-growth	0.0071	0.0429	COL3A1; COL4A1; COL6A2; COL6A1
Innate immune system			
DAP12 interactions	0.0027	0.0216	HLA-B; HLA-C; B2M
Regulation of TLR by endogenous ligand	0.0071	0.0426	FGB; S100A1; FGG
Hemostasis			
Platelet Aggregation (Plug Formation)	0.0027	0.0216	COL1A1; FGB; COL1A2; FGG
GP1b-IX-V activation signalling	0.0076	0.0454	COL1A1; COL1A2
Diseases associated with the TLR signaling cascade			
MyD88 deficiency (TLR2/4)	0.0029	0.0225	FGB; S100A1; FGG
IRAK4 deficiency (TLR2/4)	0.0032	0.0225	FGB; S100A1; FGG
Diseases associated with glycosaminoglycan metabolism			
Defective CHST3 causes SEDCJD	0.0043	0.0304	BGN; DCN
Defective CHST14 causes EDS, musculocontractural type	0.0043	0.0304	BGN; DCN
Defective CHSY1 causes TPBS	0.0053	0.0320	BGN; DCN
Signal transduction			
MET activates PTK2 signaling	0.0051	0.0315	COL1A1; COL3A1; COL1A2
Signaling by PDGF	0.0071	0.0429	COL3A1; COL4A1; COL6A2; COL6A1
Sensory perception			
Sensory processing of sound by outer hair cells of the cochlea	0.0052	0.0315	MYO1C; MYH9; MSN; EZR
Sensory processing of sound by inner hair cells of the cochlea	0.0095	0.0473	MYO1C; MYH9; MSN; EZR
Other			
Immune System	1.11E-16	1.78E-15	FGB; HLA-H; ANXA1; S100A1; FGG; HLA-B; HLA-C; MSN; TAP1; HLA-A; A1BG; COL1A1; C3; COL3A1; COL1A2; MYO1C; CA2; HLA-DRA; MYH9; ACAA1; B2M; HLA-DRB1

Supplementary Table 6. Subject characteristics for immunofluorescence protein localization

Subjects	Age	Sex	Current diagnosis	Autoantibodies	Muscle biopsied	Notes	Treatment at biopsy
sIBM	52	M	sIBM	Anti-NT5c1A	Quadriceps		None
sIBM	61	M		None	Triceps		None
sIBM	69	M		None	Biceps brachii		IVIg
sIBM	68	M		Anti-NT5c1A	Quadriceps		None
DM	18	F	DM	Anti-TIF1g	Deltoid		None
DM	50	F		Anti-Mi2	Deltoid		None
DM	42	M		Anti-Mi2	Deltoid	Polyarthritis, ILD	MMF
PM	70	M	PM	None	Deltoid	Severe respiratory muscle weakness	Prednisone 50mg/day, IVIg
OM	79	F	OM	Anti-sm, anti-dsDNA, anti-RNP, anti-Ro52	Deltoid	Sicca, Raynaud, calcinosis	Prednisone 10mg/day, IVIg, MMF

Abbreviations: sIBM, sporadic inclusion body myositis; DM, dermatomyositis; PM, polymyositis; OM, overlap myositis; ILD, interstitial lung disease; IVIg, intravenous immunoglobulins; MMF, mycophenolate mofetil.
